# Whole transcriptome profiling reveals a *lncMDP1* that regulates myogenesis by adsorbing miR-301a-5p targeting *CHAC1*

**DOI:** 10.1038/s42003-024-06226-1

**Published:** 2024-05-02

**Authors:** Bingjie Chen, Hanfang Cai, Yufang Niu, Yushi Zhang, Yanxing Wang, Yang Liu, Ruili Han, Xiaojun Liu, Xiangtao Kang, Zhuanjian Li

**Affiliations:** 1 College of Animal Science and Technology, Henan Agricultural University, Zhengzhou, 450046 China; 2 Henan Key Laboratory for Innovation and Utilization of Chicken Germplasm Resources, Zhengzhou, 450046 China

**Keywords:** Long non-coding RNAs, Transcriptomics, Bone development, Cell proliferation, Differentiation

## Abstract

Myoblast proliferation and differentiation are essential for skeletal muscle development. In this study, we generated the expression profiles of mRNAs, long noncoding RNAs (lncRNAs), and microRNAs (miRNAs) in different developmental stages of chicken primary myoblasts (CPMs) using RNA sequencing (RNA-seq) technology. The dual luciferase reporter system was performed using chicken embryonic fibroblast cells (DF-1), and functional studies quantitative real-time polymerase chain reaction (qPCR), cell counting kit-8 (CCK-8), 5-Ethynyl-2′-deoxyuridine (EdU), flow cytometry cycle, RNA fluorescence in situ hybridization (RNA-FISH), immunofluorescence, and western blotting assay. Our research demonstrated that miR-301a-5p had a targeted binding ability to *lncMDP1* and ChaC glutathione-specific gamma-glutamylcyclotransferase 1 (*CHAC1*). The results revealed that *lncMDP1* regulated the proliferation and differentiation of myoblasts via regulating the miR-301a-5p/*CHAC1* axis, and *CHAC1* promotes muscle regeneration. This study fulfilled the molecular regulatory network of skeletal muscle development and providing an important theoretical reference for the future improvement of chicken meat performance and meat quality.

## all

“A transcriptome study of chicken primary myoblast proliferation and differentiation shows that the lncMDP1/miRNA-301a-5p/CHAC1 axis plays an important role in skeletal muscle development.“

## Introduction

Skeletal muscle is the main component of meat. Both of muscle growth rate and meat quality are important economic traits in the broiler industry, so investigation of the molecular mechanisms underlying skeletal muscle growth and development can help us to improve the meat output rate and meat quality in the livestock industry^1–3^. The growth and development of skeletal muscle is a long and complex multistage process^4^. Initially, somites differentiate firstly into muscle precursor cells and then into myogenic precursors that proliferate and differentiate into myoblasts, which further differentiate into myotubes and then fuse into multinucleated myotubes, and myotubes then aggregate into bundles to form muscle fibers, and eventually mature into skeletal muscle tissue^5–7^. The proliferation and differentiation of myoblasts can simulate the growth and development of muscles, and this process is regulated by multiple molecular, including coding genes, transcription factors, signaling pathways, noncoding RNAs, and the interaction networks among them. Therefore, extensive in vitro studies performed to explore their synergistic effects provide insight into the molecular regulatory mechanisms involved in skeletal muscle development^8^.

Recent studies have shown that noncoding RNAs play a key role in the regulation of skeletal muscle growth and development. MicroRNAs (miRNAs) are a class of endogenous noncoding small noncoding RNAs that can silence RNAs and regulate gene expression at the posttranscriptional level^9–11^. LncRNAs and miRNAs can interact with each other through direct binding or competitive endogenous RNA (ceRNA) mechanisms, thereby exerting important functions such as regulating cell proliferation and differentiation, and individual development^12–14^. Recent studies have found that *lncIRS1* can act as a ceRNA of the miR-15 family, which in turn regulates the expression of the *IRS1* gene, thereby promoting skeletal muscle myogenesis for the treatment of muscle atrophy^15^. *Lnc-ORA* suppresses skeletal muscle production in mice by acting as a sponge of miR-532-3p and regulating *IGF2BP2* expression^16^. However, the biological function played by ceRNA mechanisms in the skeletal muscle development of chickens is rarely reported.

The Arbor Acres broiler (AA broiler) is a modern commercial broiler with a fast growth rate, high feed conversion rate, full development of breast and leg muscles, and good carcass quality. Chickens are often used for muscle growth and development research, in order to reveal the key genes and molecular regulatory mechanisms of chicken skeletal muscle growth and development. Therefore, in this study, chicken primary myoblasts (CPMs) at key developmental stages of proliferation and differentiation were collected for whole transcriptome sequencing, including CPMs proliferating to 50% (G1) and 100% (G2), differentiation to day 1 (D1), day 2 (D2), day 4 (D4), day 6 (D6) and day 8 (D8), which covers the pre, middle and late stages of proliferation and differentiation, which is helpful for a comprehensive understanding of the myoblast growth process. As a result, a lncRNA-miRNA-mRNA interaction network associated with chicken skeletal muscle development was drawn. These findings enrich our understanding of the regulatory network and molecular mechanisms of chicken muscle development.

## Results

### Construction of CPMs at different developmental stages

CPMs were isolated and cultured from the leg muscles of normal AA broilers at the 11 embryonic (E11). The freshly isolated myoblasts were round, fully adherent and extended into a spindle shape, and then proliferated rapidly. We collected cells when the confluence of myoblasts reached 50% and 100%. Myoblasts were spindle-shaped monocytes before the induction of differentiation. A large number of proliferating myoblasts became elongated on 1^st^ day after differentiation and presented a parallel arrangement, and the cells aggregated into bundles and became fibrillar. On the 2nd day of induction, the cells fused to form long multinucleated tubular primary myotubes, which continuously increased in size. On the 4th day, the number of myotubes further increased, and on 6th day, the myotubes became thinner and began to detach. On the 8th day, the myotubes showed a large area of detachment (Supplementary Fig. 1). The cells at different developmental times (proliferation to 50 and 100%, differentiation to D1, D2, D4, D6, and D8) were collected for RNA sequencing (RNA-seq).

### Identification of differentially expressed mRNAs, miRNAs, lncRNAs (DEmRNAs, DEmiRNAs, DElncRNAs), and construction of interactive network

**Fig. 1:**
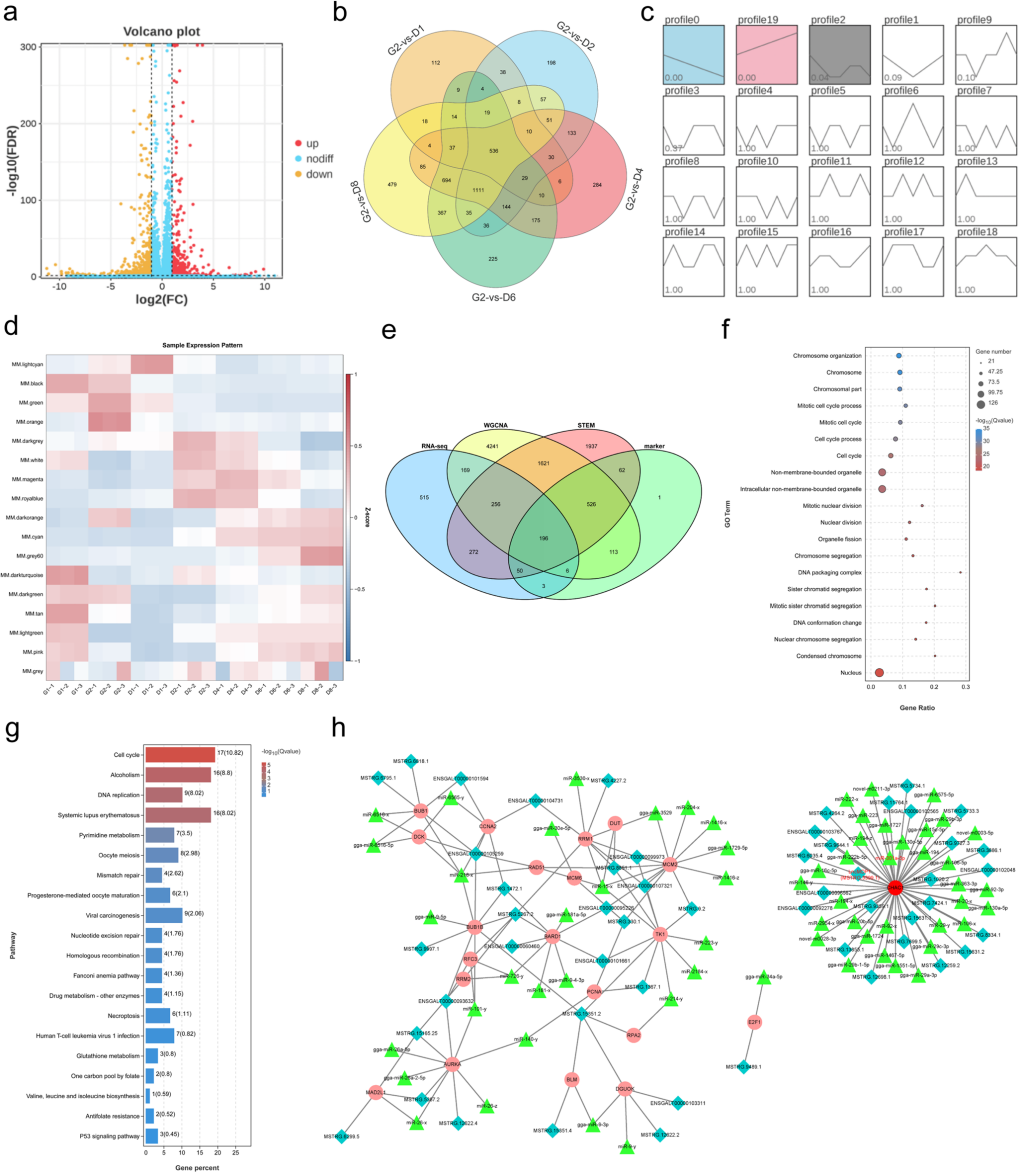
Overview of DEmRNAs, and lncRNA‒miRNA‒mRNA ceRNA network of target genes, *n* = 3 biologically independent samples. **a** Volcano plots of differentially expressed genes in the G1 and G2 groups. **b** Venn diagram for the comparison of G2 with D1, D2, D4, D6, and D8. Orange represents G2-vs.-D1, blue represents G2-vs.-D2, pink represents G2-vs.-D4, green represents G2-vs.-D6, yellow represents G2-vs.-D6. **c** Trend analysis graph of all differentially expressed mRNAs. Blue represents profile0, pink represents profile19, gray represents profile2. **d** WGCNA analyzed the gene expression profiles of CPMs at different developmental stages, and the expression patterns of the module genes in each sample were shown by module eigenvalues. The module eigenvalues in each sample reflect the combined expression levels of all genes in each sample. Pink represents high expression, blue represents low expression. **e** Venn diagrams of the four analytical methods. RNA-seq (blue) indicates the sum of differential genes for the intersection of the six different groups compared; WGCNA (yellow) indicates all genes in the six modules MM.black, MM.green, MM.magenta, MM.cyan, MM.grey60 and MM.lightgreen; STEM (pink) indicates the sum of differential genes with profile0, profile19 differential genes; maker (green) indicates the differential genes with Pearson correlation coefficient ≥ 0.95 with the expression of 14 marker genes among 6937 differential genes. **f**, **g** GO and KEGG annotations of the target gene cluster. Bubble size represents the number of differential genes enriched in GO term. Bubble color represents the significance of enrichment in GO term. The larger the value, the more significant the enrichment. Red indicates the larger the value, and blue indicates the lower the value. The color of the column indicates the enrichment significance of the Pathway. The red indicates the higher the significance, and the blue indicates the lower the significance. **h** The pink ellipses represent mRNA (*CHAC1* is represented by red ellipse), the green triangles represent miRNA (miRNA-301a-5p is represented by red font), and the blue diamonds represent lncRNA (*lncMDP1* is represented by red font).

We performed a whole-transcriptome analysis of CPMs at different differential stages. We demonstrated the sources of variance in our data by PCA analysis of two principal components (PC1 and 2). As shown in Supplementary Fig. 2, PC1, and PC2 contributed 59.7% and 24.3%, respectively. It can be seen from Supplementary Fig. 2 that the three samples we collected at each time point clustered closely together and also separated from others at different differential stages, indicating low variance in this analysis and showing good data repeatability for the following analysis. A total of 6937 DEmRNAs were obtained when the samples from every two of differential time points were compared (Supplementary Tab. 1). 1048 DEmRNAs (393 upregulated and 655 downregulated) were identified in the proliferative G1-vs.-G2 group (Fig. 1a), while the differentiation (G2-vs.-D1, G2-vs.-D2, G2-vs.-D4, G2-vs.-D6, and G2-vs.-D8) groups showed 536 possible key genes that regulated differentiation (Fig. 1b). To further explore the dynamic expression patterns of genes during the developmentally expressed stages of myoblasts, we clustered the expression patterns of all differential genes throughout the developmental stages of CPMs differentiation through trend analysis, which showed 2 highly significantly enriched gene sets, (*P* < 0.00, Fig. 1c), profile 0 and profile 19, with a total of 4920 differentially expressed genes.

In this study, genes were divided into 17 modules (color coded) with similar expression patterns via weighted gene correlation network analysis (WGCNA), as shown in the tree diagram (Supplementary Fig. 3),each branch formed a module and each leaf in the branch represents a gene. The number of genes in each module was shown in Supplementary Fig. 4. Next, we performed correlation analysis on these 17 modules (Supplementary Fig. 5). According to current researches, we selected 14 marker genes related to myoblast proliferation (*CDK1*, *CCNB1*, *PCNA*), differentiation (*MYH1C*, *MYOD1*, *MyoG*, *MCF2*, *MYH1F*, *MYH15*), fusion (*SMAD3*, *TGFB3*, *ACVR1B*), and myogenesis (*MEF2C*, *MYF6*), and their gene expression patterns were used to indicate the differential stages of CPMs (Supplementary Fig. 6)^17^. As shown in Fig. 1d we found that six modules, MM.black, MM.green, MM.magenta, MM.cyan, MM.grey60, and MM.lightgreen were significantly associated with the 14 genes representing specific developmental stages mentioned above, including 7,433 genes, so that the corresponding modules could be selected for further study. The expression levels of 6,937 differentially expressed genes were further investigated by Pearson correlation coefficient analysis with 14 marker genes, and thehighly correlated differentially expressed genes might present synergistic effect with them, and a total of 957 differential genes were obtained according to a Pearson correlation coefficient ≥0.95.

The Venn diagram results showed that 196 DEmRNAs may play critical roles throughout all developmental stages after a combined analysis based on the four methods (Fig. 1e). Gene ontology (GO) term enrichment and kyoto encyclopedia of genes and genomes (KEGG) pathway analysis of these 196 DEmRNAs were performed to investigate the important pathways and related biological functions of DEGs. GO analysis revealed that the differentially expressed mRNAs were mainly associated with cell cycle terms, including cell cycle, mitosis and DNA conformational changes (Fig. 1f). Additionally, KEGG pathway analysis showed that these mRNAs were highly enriched in the cell cycle, DNA replication, glutathione metabolism, valine, leucine, and isoleucine biosynthesis and P53 signaling pathway (Fig. 1g).

Meanwhile, we found 890 DEmiRNAs, and 942 DElncRNAs from sequencing results. The volcano plot showed that there were 145 DEmiRNAs and 117 DElncRNAs in the proliferative phase G1-vs.-G2 group (Supplementary Fig. 7a, d). The Venn diagram of the five groups (G2-vs.-D1, G2-vs.-D2, G2-vs.-D4, G2-vs.-D6, and G2-vs.-D8) in the differentiation phase showed that 50 DEmiRNA and 65 DElncRNAs might be essential in regulating differentiation (Supplementary Fig. 7b, e). To further explore the dynamic expression patterns of miRNAs and lncRNAs during chicken muscle development, we analyzed the expression patterns of all differential miRNAs and lncRNAs throughout the differentiation stages of CPMs by trend analysis. The clustering results of differentially expressed miRNAs showed 2 highly significantly enriched gene sets, profile0, and profile19, with a total of 567 differential miRNAs (Supplementary Fig. 7c). The clustering results of DElncRNAs showed 4 highly significantly enriched gene sets, profile0, profile19 with a total of 368 differential lncRNAs (Supplementary Fig. 7f).

Subsequently, we constructed the lncRNA‒miRNA‒mRNA interaction network based on the expression profiles. The interactions among lncRNAs, miRNAs, and mRNAs may control how CPMs develop. To reduce the number of possible key genes affecting the development of CPMs, we focused on the 196 key DEmRNAs described above. A total of 41 differentially expressed genes which were enriched in KEGG iterms cell growth and death, replication and repair, nucleotide metabolism and amino acid metabolism pathways were selected. Only 21 genes were included in a ceRNA network with related differential lncRNAs and miRNAs. We used Cytoscape (version 3.6.0) to construct a lncRNA‒miRNA-mRNA coexpression visualization network (Fig. 1h). Interestingly, we found that ChaC glutathione-specific gamma-glutamylcyclotransferase 1 (*CHAC1*) had the largest number of ceRNAs network, which indicated that this gene may play an important role in skeletal muscle development. There were 33 miRNAs and 24 lncRNAs in this ceRNA network produced by *CHAC1*. Among them, lncRNA-MSTRG5399.1 had a high expression level and was significantly correlaed to *CHAC1* (*r* = 0.943, *P* = 0.927), named *lncMDP1* (myoblast proliferation and differentiation 1). miR-301a-5p had a significantly negative correlation with the expression of *lncMDP1* (*P* = -0.746). Moreover, the results of the quantitative real-time polymerase chain reaction (qPCR) assay verified the corrlelation of expression among *lncMDP1*, miR-301a-5p, and *CHAC1* in RNA-seq (Supplementary Fig. 8).

### LncMDP1 promotes myoblast proliferation and differentiation

**Fig. 2:**
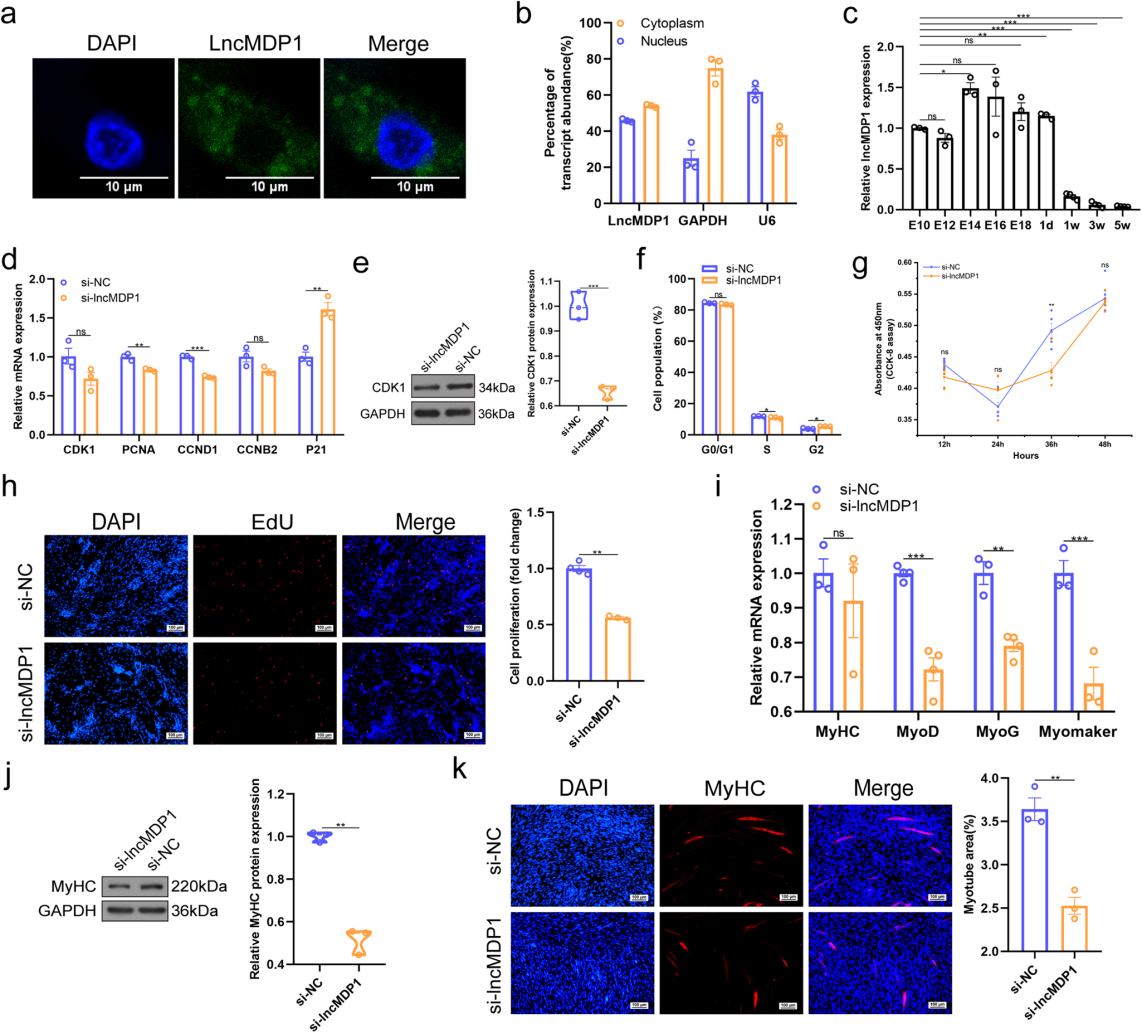
*LncMDP1* promotes the proliferation and differentiation of myoblasts. **a** RNA-FISH of *lncMDP1*. The scale is 10 μm. **b** *LncMDP1* is located in the cytoplasm and nucleus, *n* = 3 biologically independent samples. **c** RNA expression levels of *lncMDP1* in leg muscle at different times. Among them, E10, E12, E14, E16, E18 and 1d are *n* = 3 biologically independent samples, and 1w, 3w and 5w are *n* = 4 biologically independent samples. **d** Relative expression of *CDK1*, *PCNA*, *CCND1*, *CCNB2*, and *P21* 48 h after the transfection of si-*lncMDP1* in proliferating myoblasts, *n* = 3 biologically independent samples. **e** *LncMDP1* significantly reduced the protein expression level of CDK1 after interference, *n* = 3 biologically independent samples. The error bars are equivalent throughout the Figure. In the box line, a line in the middle of the box represents the median, and the upper and lower bottoms of the box are the upper quartile and the lower quartile, respectively. The blue circle represents si-NC, and the orange circle represents si-*lncMDP1*. Western blot was performed on different membranes for proteins with a molecular weight difference of fewer than 5 kDa (similar protein size, difficult to distinguish), and the sample size was consistent under the premise of detecting protein concentration. In this case, the sample and the loading control ran on different gels and thus transferred to different membranes. **f** Cell cycle analysis after the transfection of si-*lncMDP1* in myoblasts, *n* = 3 biologically independent samples. **g** CCK-8 was used to detect the effect of *lncMDP1* interference on myoblast proliferation, *n* = 5 biologically independent samples, among them, si-NC at 12 h and 24 h *n* = 6. **h** EdU staining was performed to analyze the effect on the proliferation of myoblasts after transfection with si-*lncMDP1* and si-NC at a rate of 100 μm (si-NC is *n* = 4 biologically independent samples, and si-*lncMDP1* is *n* = 3). **i** *LncMDP1* interference significantly reduced RNA expression levels of myoblast differentiation-associated marker genes, including *MyoD* (*n* = 4 biologically independent samples), *MyoG* (si-NC is *n* = 3 biologically independent samples, and si-*lncMDP1* is *n* = 4), *MyHC* (*n* = 3 biologically independent samples), and *Myomarker* (*n* = 3 biologically independent samples). **j** Knockdown of *lncMDP1* reduced the protein expression levels of MyHC, *n* = 3 biologically independent samples. The error bars are equivalent throughout the Figure. In the box line, a line in the middle of the box represents the median, and the upper and lower bottoms of the box are the upper quartile and the lower quartile, respectively. The blue circle represents si-NC, and the orange circle represents si-*lncMDP1*. **k** Immunofluorescence of myoblasts differentiated with si-*lncMDP1* and si-NC for 48 h, followed by staining with an MyHC antibody, *n* = 3 biologically independent samples. The scale is 100 μm. The results are shown as the mean ± SEM of three independent experiments. (*P < 0.05; **P < 0.01, ***P < 0.001).

We first detected the subcellular distribution of *lncMDP1* by RNA fluorescence in situ hybridization (RNA-FISH) and qPCR. The results showed that *lncMDP1* was mostly located in the cytoplasm (Fig. 2a, b). From the temporal expression profile of the leg muscles of AA broilers, it was found that *lncMDP1* was continuously highly expressed during the embryonic period, and its expression level decreased sharply 1 day after hatching. On 1st day, *lncMDP1* was relatively high in the spleen, muscular stomach, and glandular stomach, followed by the leg and breast muscles (Fig. 2c and Supplementary Fig. 9). After interfering with *lncMDP1*, the expression levels of myoblast proliferation-promoting (*CDK1*, *PCNA*, *CCND1*, *CCNB2*) and proliferation-inhibiting marker genes (*P21*) showed opposite trends (Fig. 2d) and interfering with *lncMDP1* significantly promoted CDK1 (*P* < 0.001) protein expression (Fig. 2e and Supplementary Fig. 10a, unedited original blots in Supplementary Fig. 19, Supplementary Fig. 25). *LncMDP1* interference results in a significant reduction of cells entering S phase (*P* < 0.05) in the cell cycle (Fig. 2f and Supplementary Fig. 11a). Cell counting kit-8 (CCK-8) and 5-Ethy nyl-2′-deoxyuridine (EdU) staining also showed that proliferation was significantly (*P* < 0.05) inhibited upon interference compared to control cells (Fig. 2g, h). According to qPCR analysis, interference with *lncMDP1* significantly (*P* < 0.01) inhibited myoblast differentiation, presented down-regulated expression of differentiation marker genes (*MyHC*, *MyoD*, *MyoG*, and *Myomarker*) (Fig. 2i), and significantly (*P* < 0.01) inhibited protein expression of MyHC (Fig. 2j and Supplementary Fig. 10b, unedited original blots in Supplementary Fig. 20, Supplementary Fig. 26) compared with control cells. By immunofluorescence staining, we found that interference with *lncMDP1* significantly (*P* < 0.01) reduced total myotube area and inhibited myoblast differentiation (Fig. 2k). In conclusion, the data suggest that *lncMDP1* promoted myoblast development.

### LncMDP1 might function as a sponge for miR-301a-5p

**Fig. 3:**
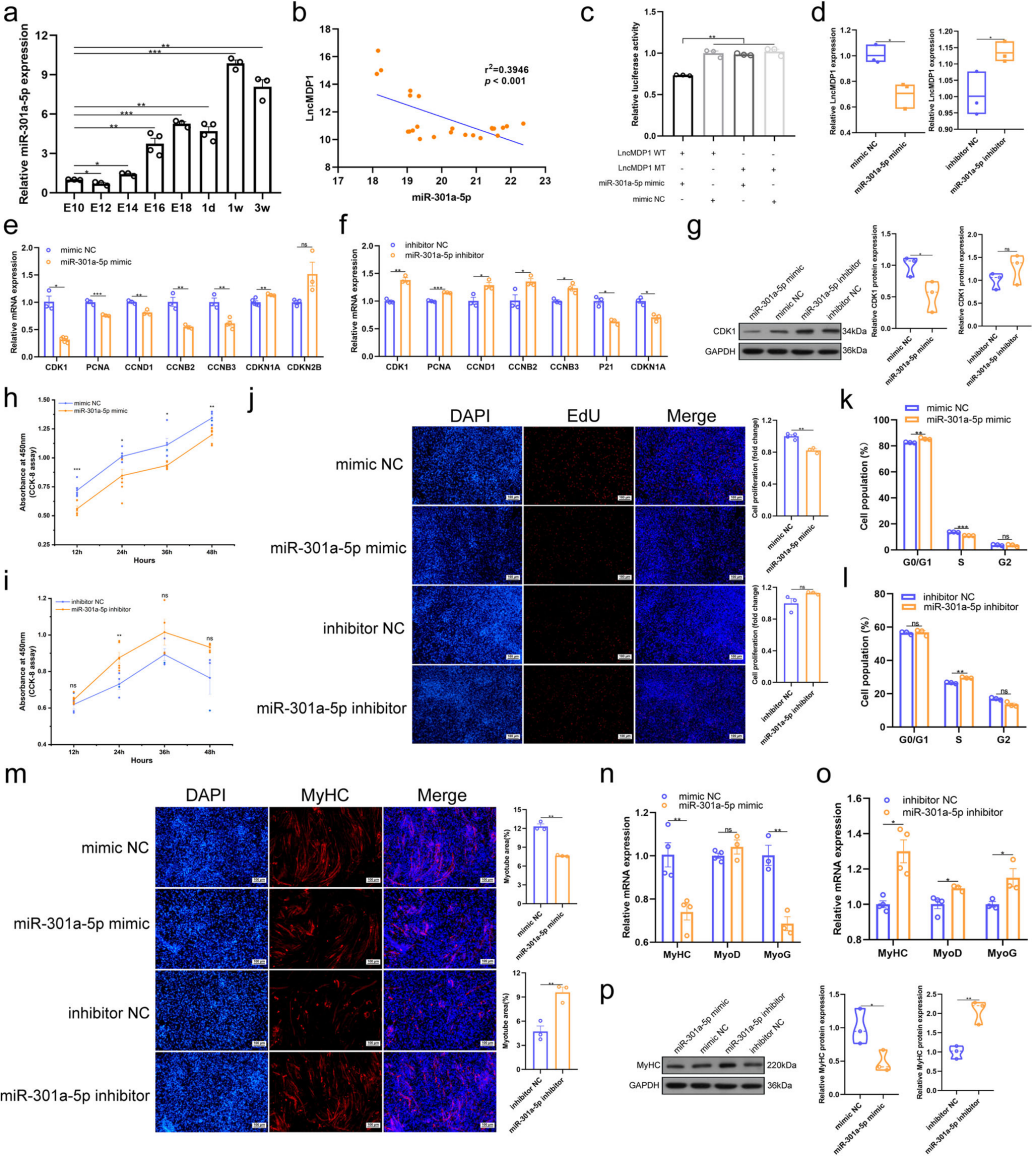
*LncMDP1* might function as a sponge for miR-301a-5p. **a** RNA expression levels of miR-301a-5p in AA broilers leg muscle at different times. Among them, E10, E12, E14, E18, 1w and 3w are *n* = 3 biologically independent samples, E16 and 1d are *n* = 4 biologically independent samples. **b** Pearson correlation between *lncMDP1* and miR-301a-5p expression profiles in the leg muscle of AA broilers was detected by qPCR (It contains the following eight periods, E10, E12, E14, E16, E18,1d, 1w and 3w). **c** The dual luciferase reporter system was used to evaluate the binding of *lncMDP1* and miR-301a-5p in DF-1 cells, *n* = 3 biologically independent samples. **d** Overexpression of miR-301a-5p decreased the RNA expression level of *lncMDP1*, and interference with miR-301a-5p had the opposite effect, *n* = 3 biologically independent samples. The error bars are equivalent throughout the Figure. In the box line, a line in the middle of the box represents the median, and the upper and lower bottoms of the box are the upper quartile and the lower quartile, respectively. The blue circle of the left box line diagram represents mimic NC, and the orange square represents miR-301a-5p mimic. The blue circle of the right box line diagram represents inhibitor NC, and the orange square represents miR-301a-5p inhibitor. **e**, **f** Relative expression levels of myoblast proliferation related marker genes *CDK1* (mimic NC, inhibitor NC and miR-301a-5p inhibitor are *n* = 3 biologically independent samples, miR-301a-5p mimic is *n* = 5), *PCNA* (*n* = 3 biologically independent samples), *CCND1* (*n* = 3 biologically independent samples), *CCNB2* (*n* = 3 biologically independent samples), *CCNB3* (mimic NC, inhibitor NC and miR-301a-5p inhibitor are *n* = 3 biologically independent samples, miR-301a-5p mimic is *n* = 4), *CDKN1A* (mimic NC is *n* = 5 biologically independent samples, miR-301a-5p mimic, inhibitor NC and miR-301a-5p inhibitor are *n* = 3), *P21* (inhibitor NC and miR-301a-5p inhibitor are *n* = 3 biologically independent samples) and *CDKN2B* (*n* = 3 biologically independent samples) after transfection with miR-181a-5p mimics and inhibitors. **g** The protein expression level of CDK1 was decreased after transfection with miR-301a-5p mimic, and transfection of miR-301a-5p inhibitors increased the protein expression level of CDK1, *n* = 3 biologically independent samples. The error bars are equivalent throughout the Figure. In the box line, a line in the middle of the box represents the median, and the upper and lower bottoms of the box are the upper quartile and the lower quartile, respectively. The blue circle of the left box line diagram represents mimic NC, and the orange circle represents miR-301a-5p mimic. The blue circle of the right box line diagram represents inhibitor NC, and the orange circle represents miR-301a-5p inhibitor. Western blot was performed on different membranes for proteins with a molecular weight difference of fewer than 5 kDa (similar protein size, difficult to distinguish), and the sample size was consistent under the premise of detecting protein concentration. In this case, the sample and the loading control ran on different gels and thus transferred to different membranes. **h**, **i** CCK-8 assays were performed to assess the effect of transfection with miR-301a-5p mimics (mimic NC is *n* = 7 biologically independent samples at 12 h and 48 h, *n* = 6 at 24 h, *n* = 5 at 36 h, miR-301a-5p mimic is *n* = 6 at 12 h and 48 h, *n* = 7 at 24 h, *n* = 5 at 36 h) and inhibitors (mimic NC is *n* = 4 biologically independent samples at 12 h, 24 h and 36 h, *n* = 6 at 48 h, miR-301a-5p mimic is *n* = 6 at 12 h and 24 h, *n* = 4 at 36 h, *n* = 5 at 36 h) on myoblast proliferation. **j** Proliferation of myoblasts transfected with miR-301a-5p mimic and inhibitor was analyzed by EdU staining at a scale of 100 μm (miR-301a-5p mimic, inhibitor NC and miR-301a-5p inhibitor are *n* = 3 biologically independent samples, mimic NC is *n* = 4). **k**, **l** Cell cycle analysis after transfection of miR-301a-5p mimics and inhibitors in myoblasts, *n* = 3 biologically independent samples. **m** MyHC immunofluorescence staining of myoblasts 48 h after transfection with miR-301a-5p mimic and inhibitor, *n* = 3 biologically independent samples. The scale is 100 μm. **n–p** Transfection with miR-301a-5p mimic significantly decreased RNA expression levels of markers associated with myoblast differentiation, *MyoD* (*n* = 4 biologically independent samples), *MyoG* (*n* = 3 biologically independent samples), and *MyHC* (*n* = 4 biologically independent samples), and decreased protein expression levels of MyHC (*n* = 3 biologically independent samples, and the error bars are equivalent throughout the Figure.) In the box line, a line in the middle of the box represents the median, and the upper and lower bottoms of the box are the upper quartile and the lower quartile, respectively. The blue circle of the left box line diagram represents mimic NC, and the orange circle represents miR-301a-5p mimic. The blue circle of the right box line diagram represents inhibitor NC, and the orange circle represents miR-301a-5p inhibitor. The results of transfection of miR-301a-5p inhibitor were the opposite. Figure 3g, p was from different blots and since the molecular weight difference between CDK1 and GAPDH was only 2 kDa, it was not easy to distinguish between them, so the same internal control (GAPDH) was used to ensure that the sample load is consistent under the premise of detecting the protein concentration. The findings are presented as the mean ± SEM of three separate experiments. (* *P* < 0.05; ** *P* < 0.01, *** P < 0.001).

We first examined the biological expression characteristics of miR-301a-5p in chickens. The temporal expression profile of miR-301a-5p in leg muscles showeds a gradual increase. Its expression after birth was significantly higher than the embryonic period. Moreover, we also found that miR-301a-5p was significantly negatively correlated with *lncMDP1* expression in the leg muscles of AA broilers (Fig. 3a, b, and Supplementary Fig. 12). Subsequently, we predicted the binding site of *lncMDP1* to miR-301a-5p by bioinformatics analysis (Supplementary Fig. 13a). Their relationship was validation by dual luciferase reporter gene analysis and qPCR showed that *lncMDP1* and miR-301a-5p could interact directly, and miR-301a-5p could influence the mRNA expression of *lncMDP1* (Fig. 3c, d). To further observe the effect of miR-301a-5p on the proliferation and differentiation of myoblasts, we transfected myoblasts with miRNA mimics or negative control miRNA (miR-NC). It was found that the change of expression of marker genes were reversed (Fig. 3e, f). Also, miR-301a-5p significantly (*P* < 0.05) decreased the protein expression level of CDK1 (Fig. 3g and Supplementary Fig. 14a, unedited original blots in Supplementary Fig. 21, Supplementary Fig. 28). Cell proliferation were then measured by flow cytometry analysis, the CCK-8 assay, and EdU staining. The CCK-8 and EdU results showed that transfection with miR-301a-5p mimic significantly (*P* < 0.05) decreased myoblast proliferation compared with transfection with miR-NC (Fig. 3h, j), and the results of treatment with miR-301a-5p inhibitors were opposite (Fig. 3i, j). Flow cytometry analysis of the cell cycle showed that myoblasts transfected with a miR-301a-5p mimic were arrested in S phase (Fig. 3k and Supplementary Fig. 11b), and the opposite result was observed after transfection with a miR-301a-5p inhibitor (Fig. 3l and Supplementary Fig. 11c). After immunofluorescence staining, we found that miR-301a-5p overexpression inhibited myoblast differentiation and significantly reduced the total myotubes area, whereas the opposite result was obtained after the interference of miR-301a-5p (Fig. 3m). The expression of myoblast differentiation marker genes (*MyoD*, *MyHC*, *MyoG*) was downregulated after transfection with miR-301a-5p mimics (Fig. 3n), and the protein expression level of MyHC was significantly (*P* < 0.05) reduced (Fig. 3p and Supplementary Fig. 14b, unedited original blots in Supplementary Fig. 21, Supplementary Fig. 29); the results were reversed with the treatment of miR-301a-5p interference (Fig. 3o, p and Supplementary Fig. 14b, unedited original blots in Supplementary Fig. 21, Supplementary Fig. 29). In a conclusion, the data suggested that *lncMDP1* act as a sponge for miR-301a-5p, and miR-301a-5p inhibits the proliferation and differentiation of myoblasts.

### LncMDP1 regulateed myoblast proliferation and differentiation through the miR-301a-5p/CHAC1 axis

**Fig. 4:**
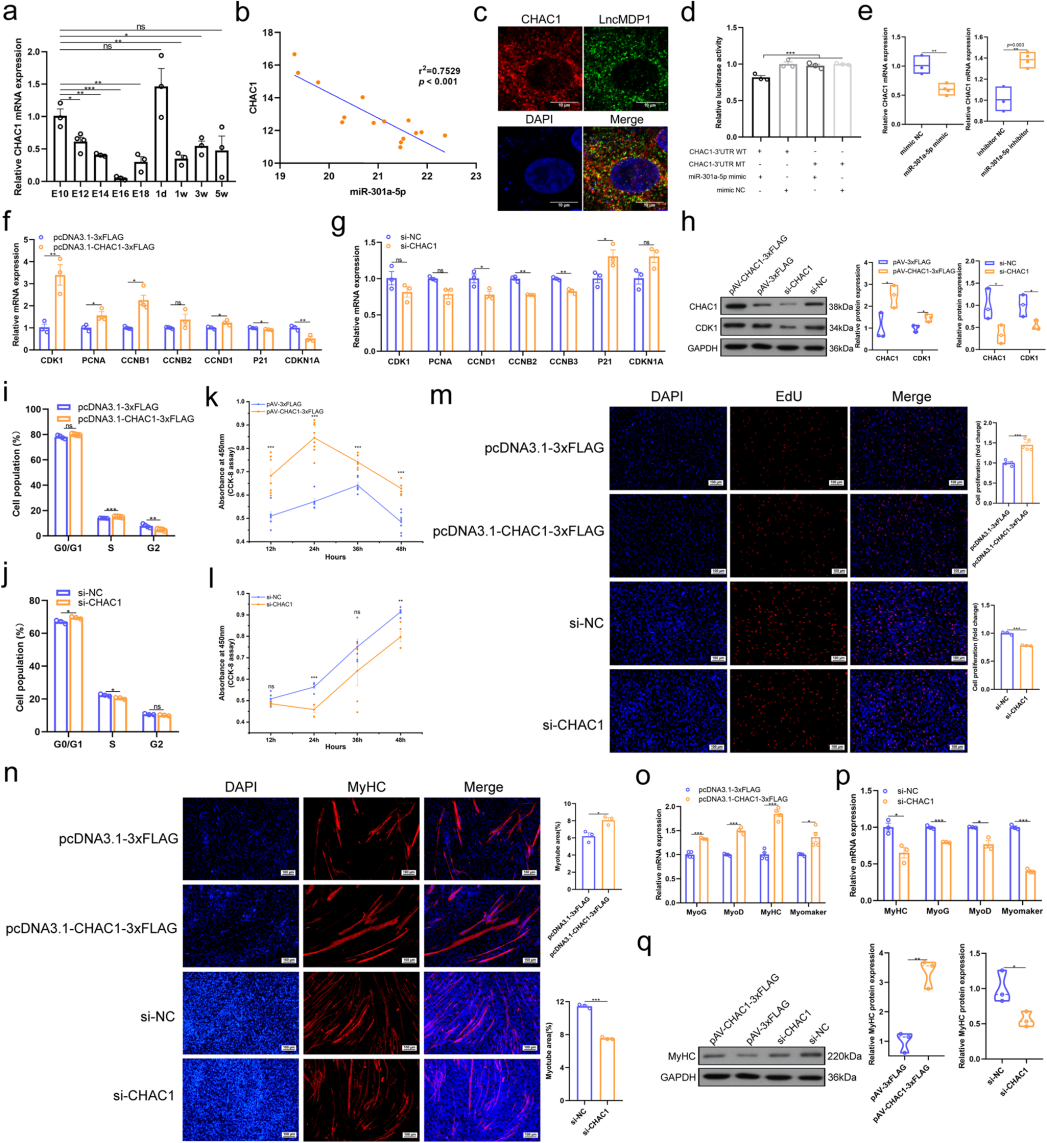
*LncMDP1* regulates *CHAC1* expression through the adsorption of miR-301a-5p. **a** RNA expression levels of miR-301a-5p in AA broilers leg muscle at different times, *n* = 3 biologically independent samples. **b** Pearson correlation between *lncMDP1* and miR-301a-5p expression at the embryonic stage of AA broilers leg muscle using qPCR (It contains the following five periods, E10, E12, E14, E16 and E18). **c** RNA in situ hybridization was used to detect the co-localization between *lncMDP1* and *CHAC1* in myoblasts. The scale is 10 μm. **d** The dual luciferase reporter system was used to assess the binding of *CHAC1* and miR-301a-5p in DF-1 cells, *n* = 3 biologically independent samples. **e** Overexpression of miR-301a-5p decreased the RNA expression level of *CHAC1*, and knockdown of miR-301a-5p had the opposite effect, *n* = 3 biologically independent samples. The error bars are equivalent throughout the Figure. In the box line, a line in the middle of the box represents the median, and the upper and lower bottoms of the box are the upper quartile and the lower quartile, respectively. The blue circle of the left box line diagram represents mimic NC, and the orange square represents miR-301a-5p mimic. The blue circle of the right box line diagram represents inhibitor NC, and the orange square represents miR-301a-5p inhibitor. **f**, **g** Relative expression of *CDK1* (*n* = 3 biologically independent samples), *PCNA* (*n* = 3 biologically independent samples), *CCND1* (*n* = 3 biologically independent samples), *CCNB1* (*n* = 4 biologically independent samples), *CCNB2* (pcDNA3.1-3xFLAG is *n* = 4 biologically independent samples, pcDNA3.1-*CHAC1*-3xFLAG, si-NC and si-*CHAC1* are *n* = 4), *P21* (*n* = 3 biologically independent samples), *CDKN1A* (*n* = 3 biologically independent samples) and other genes after the transfection of pcDNA3.1-*CHAC1*-3xFLAG or si-*CHAC1* in myoblasts. **h** Protein expression levels of CDK1 and CHAC1 after *CHAC1* overexpression or interference, *n* = 3 biologically independent samples. The error bars are equivalent throughout the Figure. In the box line, a line in the middle of the box represents the median, and the upper and lower bottoms of the box are the upper quartile and the lower quartile, respectively. The blue circle of the left box line diagram represents pAV-3xFLAG, and the orange circle represents pAV-*CHAC1*-3xFLAG. The blue circle of the right box line diagram represents si-NC, and the orange circle represents si-*CHAC1*. Western blot was performed on different membranes for proteins with a molecular weight difference of fewer than 5 kDa (similar protein size, difficult to distinguish), and the sample size was consistent under the premise of detecting protein concentration. In this case, the sample and the loading control ran on different gels and thus transferred to different membranes. **i**, **j** Analysis of the cell cycle following transfection of myoblasts with si-*CHAC1* (*n* = 3 biologically independent samples) or pcDNA3.1-*CHAC1*-3xFLAG (*n* = 4 biologically independent samples). **k**, **l** CCK-8 assay was used to detect the effect of *CHAC1* overexpression (pcDNA3.1-3xFLAG is *n* = 6 biologically independent samples at 12 h,24 h and 36 h, *n* = 7 at 48 h, *n* = 5 at 36 h, pcDNA3.1-*CHAC1*-3xFLAG is *n* = 7 at 12 h and 36 h, *n* = 8 at 24 h and 48 h) or interference (*n* = 5 biologically independent samples) on myoblast proliferation. **m** Transfection of pcDNA3.1-*CHAC1*-3xFLAG (*n* = 5 biologically independent samples, pcDNA3.1-3xFLAG is *n* = 4) or si-*CHAC1* (*n* = 3 biologically independent samples) was analyzed for proliferation of myoblasts by EdU staining at a scale of 100 μm. **n** MyHC staining of myoblasts after the overexpression or interference of *CHAC1* *n* = 3 biologically independent samples. The scale is 100 μm. **o–q** *CHAC1* promotes the RNA expression of genes related to myoblast differentiation, including *MyoD* (*n* = 3 biologically independent samples), *MyoG* (pcDNA3.1-3xFLAG is *n* = 4 biologically independent samples, pcDNA3.1-*CHAC1*-3xFLAG, si-NC and si-*CHAC1* are *n* = 4), *MyHC* (pcDNA3.1-*CHAC1*-3xFLAG is *n* = 4 biologically independent samples, pcDNA3.1-3xFLAG, si-NC and si-*CHAC1* are *n* = 3), and *MyoMarker* (pcDNA3.1-*CHAC1*-3xFLAG is *n* = 4 biologically independent samples, pcDNA3.1-3xFLAG, si-NC and si-*CHAC1* are *n* = 3), and decreases the protein expression levels of MyHC, *n* = 3 biologically independent samples, the error bars are equivalent throughout the Figure. In the box line, a line in the middle of the box represents the median, and the upper and lower bottoms of the box are the upper quartile and the lower quartile, respectively. The blue circle of the left box line diagram represents pAV-3xFLAG, and the orange circle represents pAV-*CHAC1*-3xFLAG. The blue circle of the right box line diagram represents si-NC, and the orange circle represents si-*CHAC1*. Figure 4h, q was from different blots. Since the molecular weight difference of GAPDH is 2 kDa from CDK1 and CHAC1, respectively, it was not easy to distinguish between them, so the same internal control (GAPDH) was used to ensure that the protein concentration was detected. The findings are presented as the mean ± SEM of three separate experiments. (*P < 0.05; **P < 0.01, ***P < 0.001).

The temporal expression profile of *CHAC1* in the leg muscles showed a downward trend with the embryonic anddevelopment and was significantly negatively correlated with the expression of miR-301a-5p, while it showed a decreasing and then increasing trend after incubation (Fig. 4a, b). Expression was high in the leg and breast muscles in 1-day-old tissues (Supplementary Fig. 15). It supposed that *CHAC1* was involved in both chicken embryonic and postnatal muscle development. We predicted the binding site of *CHAC1* to miR-301a-5p by bioinformatics analysis (Supplementary Fig. 13b). To further validate the interaction relationship between *lncMDP1*/miR-30a-5p/*CHAC1*, we examined it by RNA-FISH and the results showed that *lncMDP1* and *CHAC1* were co-localized in intracellular (Fig. 4c). Meanwhile, dual luciferase reporter gene analysis and qPCR showed that *CHAC1* and miR-301a-5p could interact directly, and miR-301a-5p could influence the mRNA expression of *CHAC1* (Fig. 4d, e). After *CHAC1* overexpression or interference in myoblasts, we found that the expression levels of pro-proliferation (*CDK1*, *PCNA*, *CCND1*, *CCNB1*, *CCNB2*) and anti-proliferation marker genes (*P21*, *CDKN1A*) showed the opposite trend (Fig. 4f, g). In addition, the protein expression level of CDK1 and CHAC1 presented a significant (*P* < 0.05) upregulation after *CHAC1* overexpression. However, an opposite result was obtained after interference of *CHAC1* (Fig. 4h and Supplementary Fig. 16a, unedited original blots in Supplementary Fig. 22, Supplementary Fig. 30, Supplementary Fig. 31). Overexpression of *CHAC1* significantly (*P* < 0.001) increased the number of cells in S phase (Fig. 4i and Supplementary Fig. 11d), whereas the number of cells in S phase significantly (*P* < 0.05) reduced after *CHAC1* interference (Fig. 4j and Supplementary Fig. 11e). The flow cytometry and CCK8 results showed that *CHAC1* overexpression significantly (*P* < 0.01) promoted myoblast proliferation (Fig. 4k) and that the interference of *CHAC1* inhibited myoblast proliferation (Fig. 4l). EdU staining results showed that overexpression of *CHAC1* significantly (*P* < 0.001) promoted cell increase compared with the control group. Conversely, interference with *CHAC1* significantly inhibited myoblast proliferation (Fig. 4m). By immunofluorescence staining, we found that overexpression of *CHAC1* significantly (*P* < 0.05) increased the total myotube area and promoted myoblast differentiation, whereas *CHAC1* interference decreased myoblast differentiation (Fig. 4n). Compared with controls, *CHAC1* overexpression significantly promoted myoblast differentiation, significantly (*P* < 0.05) up-regulated the expression of differentiation-associated marker genes (*MyHC*, *MyoD*, *MyoG*, and *Myomarker*), and up-regulated the protein expression of MyHC, while the interference of *CHAC1* downregulated their expression (Fig. 4o–q and Supplementary Fig. 16b, unedited original blots in Supplementary Fig. 22, Supplementary Fig. 32). In summary, the results of *CHAC1* and *lncMDP1* interference were consistent. Combined with the analysis of miR-301a-5p, it was concluded that *lncMDP1* acted as a molecular sponge to adsorb miR-301a-5p affecting the proliferation and differentiation of myoblasts to regulate *CHAC1*.

### CHAC1 promotes skeletal muscle regeneration

**Fig. 5:**
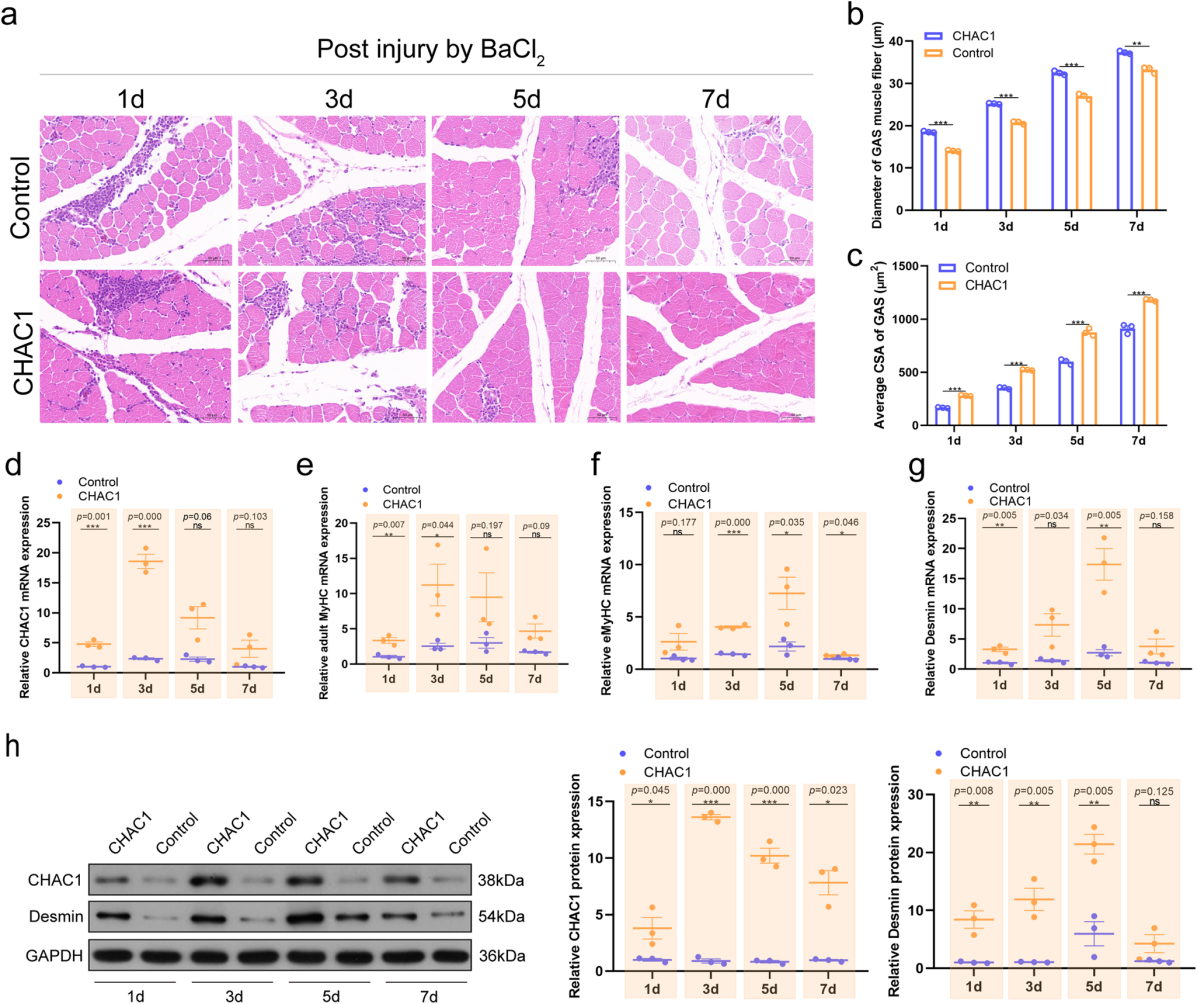
*CHAC1* promotes skeletal muscle regeneration. **a** H&E staining of chicken GAS muscle sections at days 1, 3, 5, and 7 after BaCL_2_ injury following pAV-*CHAC1*-3xFLAG injection, pAV-3xFLAG as control, scale bar 50 μm. **b** Quantification of GAS fiber diameter and (**c**) muscle fiber CSA, *n* = 3 biologically independent samples. **d** mRNA expression levels of *CHAC1*, *n* = 3 biologically independent samples. **e–g** mRNA expression levels of muscle regeneration marker genes *aMyHC*, *eMyHC*, and *Desmin*, *n* = 3 biologically independent samples. **h** Protein expression levels of CHAC1 and Desmin, *n* = 3 biologically independent samples. Western blot was performed on different membranes for proteins with a molecular weight difference of fewer than 5 kDa (similar protein size, difficult to distinguish), and the sample size was consistent under the premise of detecting protein concentration. In this case, the sample and the loading control ran on different gels and thus transferred to different membranes. The results are shown as the mean ± SEM of three independent experiments. (*P < 0.05; **P < 0.01, ***P < 0.001).

To further investigate the biological function of *CHAC1* in the regeneration of skeletal muscle, firstly, we injected BaCl_2_ into the gastrocnemius (GAS) muscle of AA broilers to cause muscle damage after injection of the adeno virus *CHAC1*-3xFLAG. Subsequently, we performed H&E staining on GAS muscles at four different time points. It was found that with the time change, the diameter of muscle fibers and the cross-sectional area (CSA) of muscle fibers in the GAS overexpressing *CHAC1* gradually increased compared to the control group. By 7th day, the inflammatory cells in the GAS overexpressing *CHAC1* were reduced and gradually replaced by newly formed muscle fibers, and the muscle fiber structure became intact and clear (Fig. 5a–c). Meanwhile, qPCR and western blotting results showed that *CHAC1* expression was significantly upregulated during muscle injury compared to controls and peaked at 3rd day (Fig. 5d, h, and Supplementary Fig. 17, unedited original blots in Supplementary Fig. 23, Supplementary Fig. 33). In addition, we also examined marker genes associated with promoting muscle regeneration and showed that GAS muscle after overexpression of *CHAC1* was significantly upregulated compared to controls in terms of expression of adult *MyHC* (*aMyHC*), embryonic *MyHC* (*eMyHC*) and *Desmin* (Fig. 5e–h and Supplementary Fig. 17, unedited original blots in Supplementary Fig. 23, Supplementary Fig. 34). Taken together, *CHAC1*significantly promote muscle regeneration.

### LncMDP1 acts as a miR-301a-5p sponge to attenuate its inhibitory of CHAC1

**Fig. 6:**
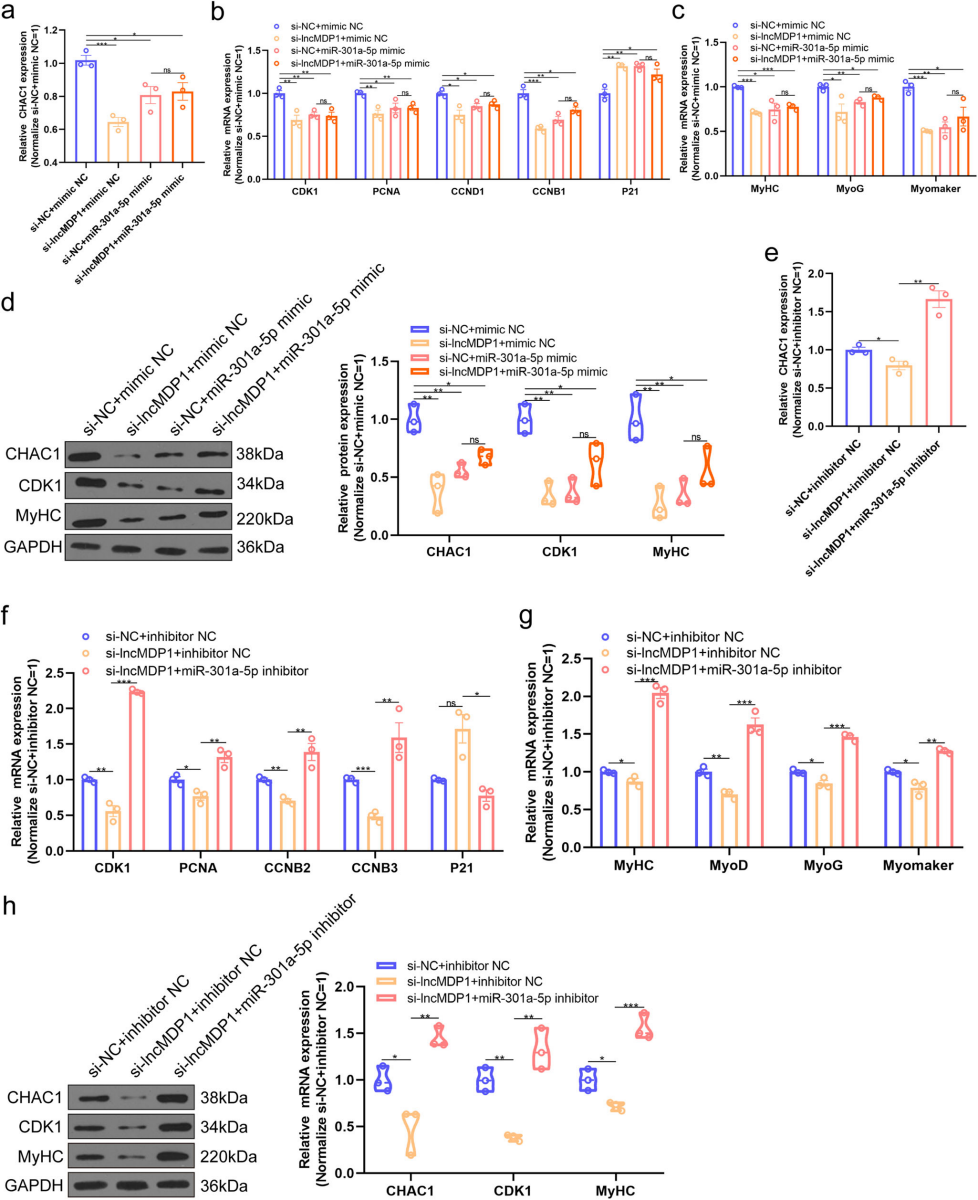
*LncMDP1* acts as a miR-301a-5p sponge to attenuate its inhibitory of *CHAC1.* **a–d** The mRNA and protein expression levels of *CHAC1* and proliferation and differentiation marker genes after cotransfection with si-*lncMDP1*, si-NC, miR-301a-5p mimic, and mimic NC, *n* = 3 biologically independent samples. The error bars are equivalent throughout the Figure. In the box line, a line in the middle of the box represents the median, and the upper and lower bottoms of the box are the upper quartile and the lower quartile, respectively. The blue circle represents si-NC and mimic NC, the yellow circle represents si-*lncMDP1* and mimic NC, the pink circle represents si-NC and miR-301a-5p mimic, and the orange circle represents si-*lncMDP1* and miR-301a-5p mimic. Western blot was performed on different membranes for proteins with a molecular weight difference of fewer than 5 kDa (similar protein size, difficult to distinguish), and the sample size was consistent under the premise of detecting protein concentration. In this case, the sample and the loading control ran on different gels and thus transferred to different membranes. **e–h** The mRNA and protein expression levels of *CHAC1* and proliferation and differentiation marker genes after cotransfection with si-*lncMDP1*, si-NC, miR-301a-5p inhibitor, and inhibitor NC, *n* = 3 biologically independent samples. The results are shown as the mean ± SEM of three independent experiments. The error bars are equivalent throughout the Figure. In the box line, a line in the middle of the box represents the median, and the upper and lower bottoms of the box are the upper quartile and the lower quartile, respectively. The blue circle represents si-NC and inhibitor NC, the yellow circle represents si-*lncMDP1* and inhibitor NC, and the pink circle represents si-*lncMDP1* and miR-301a-5p inhibitor. Western blot was performed on different membranes for proteins with a molecular weight difference of fewer than 5 kDa (similar protein size, difficult to distinguish), and the sample size was consistent under the premise of detecting protein concentration. In this case, the sample and the loading control ran on different gels and thus transferred to different membranes. (*P < 0.05; **P < 0.01, ***P < 0.001).

To further determine that *lncMDP1* attenuates its inhibitory effect on *CHAC1* by adsorption of miR-301a-5p, we performed a functional rescue experiment. The results of qPCR and western blotting showed that the expression of *CHAC1* was significantly decreased by transfection with si-*lncMDP1* and miR-301a-5p mimic compared to si-NC and mimic NC (*P* < 0.05). The expression of pro-proliferative (*CDK1*, *PCNA*, *CCND1*, and *CCNB1*) and pro-differentiation (*MyHC*, *MyoG*, and *Myomaker*) marker genes were also significantly (*P* < 0.05) decreased while the expression of the proliferation inhibitory marker gene *P21* was significantly (*P* < 0.05) increased, indicating that interference with *lncMDP1* enhanced the inhibitory effect of miR-301a-5p on *CHAC1* (Fig. 6a–d and Supplementary Fig. 18a, unedited original blots in Supplementary Fig. 24, Supplementary Fig. 35–37), consistent with the above results after interference with *CHAC1*. Meanwhile,cotransfection of *lncMDP1* rescued the inhibitory effect of miR-301a-5p on *CHAC1* and then restored the role of *CHAC1* on proliferation and differentiation of CPMs (Fig. 6e–h and Supplementary Fig. 18b, unedited original blots in Supplementary Fig. 25, Supplementary Fig. 38–40). In summary, these results suggested that *lncMDP1* acted as a sponge for miR-301a-5p and attenuate the inhibitory effect of miR-301a-5p *CHAC1*, thereby promoting the proliferation and differentiation of CPMs.

## Discussion

Sequencing technology has long played an important role in various studies of biology, providing many new insights that will help us understand complex biological systems^18^. Transcriptomics can be used to explore the interaction between mRNA and non-coding RNA^19^. Studies have shown that in mammals there are less than 2% of the genome-encoding proteins^20^. Researchers have found that not only a large number of mRNAs are the main regulators in the growth and development of skeletal muscle, but also many ncRNAs play an important synergistic regulatory role^21^. However, there are few studies on ncRNA in skeletal muscle development. In our study, to find the key factors affecting the growth and development of skeletal muscle in chickens, whole transcriptome sequencing was performed on seven stages (G1, G2, D1, D2, D3, D4, D6, D8) of proliferation and differentiation of CPMs. A total of 21 cDNA libraries were obtained to understand the dynamic expression profiles of mRNA, miRNA, and lncRNA during myoblast development. A total of 6,937 DEmRNAs, 890 DEmiRNAs, and 942 DElncRNAs related to myoblast proliferation and differentiation were identified.

In this study, we identified a total of 196 DEmRNAs by comparing differentially expressed genes in the proliferative and differentiation periods. GO and KEGG enrichment analysis indicated that these DEmRNAs weresignificantly enriched in the cell cycle, p53 signaling pathway, DNA replication, and pathways related to amino acid metabolism, and so on. It has been shown that all these pathways play an important role in regulating skeletal muscle development^22–25^. For example, the proliferation and differentiation of myoblasts are closely related to the cell cycle, and muscle-specific transcription is activated when myoblasts are blocked from entering the S phase, causing them to stagnate in G0/G1 phase^26,27^. miR-16-5p can directly target *SESN1* to regulate the p53 signaling pathway to inhibit myoblasts proliferation and differentiation, and promote apoptosis^28^. It has been shown that *CDK1* plays an important role in myoblasts proliferation, myofiber hypertrophy, and muscle regeneration^29,30^. *PCNA* can regulate the cell cycle and thus the proliferation of skeletal muscle myoblasts^31^. Therefore, the differentially expressed genes that we found to be enriched in these signaling pathways are likely to be involved in muscle growth and development.

In recent years, large amounts of research data have demonstrated that some lncRNAs play an important role in the myogenesis and hypertrophy of skeletal muscle by affecting the proliferation and differentiation of myoblasts^32–34^. In our study, a total of 942 lncRNAs were identified that were differentially expressed during the growth and development of CPMs. *LncMDP1* was significantly upregulated during CPMs differentiation, suggesting that it may influenc skeletal muscle growth and development. Through our studies in vitro, it was shown that *lncMDP1* significantly inhibited myoblast proliferation and differentiation after it was disturbed. Additionally, this lncRNA was highly expressed in the leg muscles of AA broilers at the embryonic stage, and its expression gradually decreased after birth.

Currently, studies, the ceRNA network was identified to be a key regulatorymechanisms throughout the development of organisms, including cancer treatment, cell proliferation, differentiation, and growth and development^35–37^. For example, lncRNA-*Six1* can act as a molecular sponge for miR-1611, thereby participating in the regulation of *Six1* expression and regulating skeletal muscle development^38^. *Lnc-mg* promotes myogenesis and acts as a ceRNA competitively binding miR-125b thereby regulating *IGF2* expression^39^. LncRNA *MEG3* regulates the expression of *ABCA1* through the adsorption of miR-361-5p, thereby regulating smooth muscle cell development^40^. A key ceRNA network of lncRNAs‒miRNAs‒mRNAs that may be involved in the growth and development of CPMs was constructed in our study, and *CHAC1* was the core gene in this network, which is significantly enriched in glutathione metabolic pathway. It has been shown that *CHAC1* may be involved in the regulation of muscle growth and development in zebrafish^41^. Within this network, based on sequencing data and real-time fluorescence quantification, miR-301a-5p and *lncMDP1* and *CHAC1* showed opposite expression patterns in the CPMs induction differentiation model, while miR-301a-5p was negatively correlated with *lncMDP1* and *CHAC1* expression in AA chicken leg muscle, suggesting that they may be involved through interacting in the developmental regulation of myoblast relationships. In this study, we confirmed the presence of miR-301a-5p binding sites to *lncMDP1*, and *CHAC1* targets by qPCR and dual luciferase reporter gene assay. Results from in vitro experiments showed that *CHAC1* and *lncMDP1* could promote the proliferation and differentiation of myoblasts, in contrast to the biological function of miR-301a-5p. Furthermore, we found that *CHAC1* could promote muscle regeneration by inducing muscle damage in chickens. Finally, it was further confirmed by rescue assays that *lncMDP1* regulates the expression of *CHAC1* by adsorbing miR-301a-5p. Our results suggested that *lncMDP1*, as a miR-301a-5p sponge, has a timulative effect on *CHAC1*, which in turn promotes the proliferation, differentiation, and muscle regeneration of CPMs.

In a conclusion, we systematically constructed a panorama of proliferation and differentiation of CPMs (mRNA, miRNA, lncRNA) by whole transcriptome sequencing analysis of a total of 21 samples from 7 different growth and developmental stages of CPMs. And a lncRNA named *lncMDP1* was identified, which was consistently upregulated during myoblasts differentiation and could act as a ceRNA that regulates *CHAC1* by competitively binding miR-301a-5p, thereby promoting myoblast proliferation, differentiation, and muscle regeneration. Our findings further enrich the understanding of the regulatory network and molecular mechanism of chicken skeletal muscle and provide a theoretical basis and new ideas for improving chicken meat performance and quality in the future.

## Methods

### Cell culture

CPMs were isolated from the leg muscles of 11-day-old chicken embryos (Henan Yue Poultry Agriculture and Animal Husbandry Group Co., Ltd, Jiaozuo, China)^42^. After the skin was removed, the leg muscles were placed in a Petri dish containing high-sugar DMEM (HyClone, Logan, UT) supplemented with 15% FBS (BI, Kibbutz BeitHaemek, Israel) and 0.2% penicillin/streptomycin (Solarbio, Beijing, China), and the bones were removed with forceps. The hamstring tissues were cut into 1.5 mL centrifuge tubes and subsequently transferred to a 50 mL centrifuge tube. The suspension was vortexed 30-40 times and then passed through a 70 mm sieve to obtain a single cell suspension. This procedure was repeated 3 times, each time adding the appropriate amount of prepared complete high-sugar DMEM to obtain additional cells. Cell pellets were collected by centrifugation at room temperature and the liquid was discarded. The cells were resuspended in complete high-sugar DMEM and placed in cell culture flasks. Finally, sequential differential replating was performed three times to remove fibroblasts, and the final obtained CPMs were cultured in a humidified environment at 37 °C in a 5% CO_2_ incubator. Cell samples were collected daily, depending on the degree of cell fusion observed (50% or 100%), and during days 1, 2, 4, 6, and 8 of CPMs differentiation (induction of myoblast differentiation with 2% horse serum medium). Three biological replicates were collected at each time point for whole transcriptome sequencing.

### The mRNA, lncRNA, and miRNA sequencing analysis

The quality of RNA obtained from CPMs was checked with RNase-free agarose gel electrophoresis as assessed using an Agilent 2100 Bioanalyzer (Agilent Technologies, Palo Alto, USA). Reads were then filtered with fastp (version 1.18.0) to get high quality and clean reads. Mapping reads to the rRNA database was performed using Bowtie2 (version 2.2.8). The coding capability of novel transcripts was anticipated by two softwares, CPC and CNCI. Differential expression of mRNA and lncRNA between two different groups was analyzed with DESeq2 software, and differential expression analysis of mRNA and lncRNA between two samples was determined with edgeR^43^. We judged mRNA and lncRNA to be different separately subject to the conditions of false discovery rate (FDR) < 0.05 and absolute (FC) ≥ 2 DEmiRNAs analysis between groups and samples were performed in the same way as above, and DEmiRNAs were identified with a fold change (FC) ≥ 2 and P value < 0.05. Their target genes were analyzed and predicted using both Miranda (version 3.3a) and TargetScan (version 7.0) software^44^.

### GO and KEGG pathway enrichment analysis

DEmRNAs were subjected to GO analysis through the Gene Ontology database^45^, and their enrichment pathway analysis was performed using KEGG pathway analysis^46^.

### WGCNA

WGCNA can be used to analyze and characterize patterns of correlation between genes in large sample sizes. It can also identify highly correlated gene modules and correlate modules with sample expression patterns for analysis. The intramodal connectivity (K.in) and modular correlation (MM) were calculated for all genes using the R package of WGCNA, and hub genes tend to be characterized by high connectivity and may play an important role. The visualization of the network was implemented using Cytoscape_3.3.0^47^.

### Trend analysis

The expression patterns of genes across multiple samples were analyzed using gene expression pattern analysis, We used Short Time-series Expression Miner software (STEM, version 1.3.13) for clustering analysis to identify the expression patterns of differential genes^48^. We considered clustering profiles with *p*-values ≤ 0.05 as significant profiles.

### Construction of lncRNA-miRNA-mRNA interaction network

Previous research has demonstrated that mRNAs and noncoding RNAs with one or more common miRNA response elements (MREs) can competitively bind to miRNAs and regulate one another as ceRNAs^49^. To ascertain the correlations between miRNA‒mRNA or miRNA‒lncRNA, interaction networks could be used to predict the probable roles of the expressed genes. Before analyzing the positive correlation relationship between the expression levels of potential ceRNAs, the targeting relationship between miRNAs and candidate ceRNAs (lncRNA, mRNA) and the negative correlation relationship between expression levels were examined. Finally, we performed the construction of ceRNA regulatory networks using the obtained candidate ceRNAs as well as their shared miRNA pairs (i.e., lncRNA‒miRNA-mRNA relationship pair). Visualization networks were plotted using Cytoscape software (v3.6.0). (http://www.cytoscape.org/).

### RNA extraction, cDNA synthesis, and qPCR

Total RNA in AA broiler tissues and CPMs was extracted using TRIzol reagent (Takara, Tokyo, Japan). The amount of RNA was measured with a spectrophotometer (Thermo, Waltham, USA). Using the PrimeScript RT kit and gDNA eraser, synthesized cDNA was reverse transcribed (Takara, Tokyo, Japan). The ReverTra Ace qPCR RT kit was used to reverse-transcribe miRNA (Takara, Tokyo, Japan). qPCR was performed using SYBR Green master mix (Takara, Tokyo, Japan) and LightCycler 96 qPCR system (Roche, Basel, Switzerland) for experiments. mRNA and miRNA internal reference genes were *GAPDH* and U6. All experiments were performed in triplicate. The relative quantification of genes was performed using the 2^-ΔΔCt^ method^50^. Specific primers, mimics, and inhibitors dedicated to BmLge-Loop miRNA qPCR were designed by RiboBio (RiboBio, Guangzhou, China). The primer sequences are listed in Supplementary Tab. 2.

### Plasmid construction and dual‑luciferase reporter assay

The full-length coding sequence (CDS, NM_001199656.2) of ChaC glutathione-specific gamma-glutamylcyclotransferase 1 (*CHAC1*) and partial sequences of *lncMDP1* were amplified from chicken leg muscle cDNA to construct an overexpression vector, which was ligated to the pcDNA3.1-3xFLAG vector (Promega, Madison, USA) via two restriction enzyme sites, *EcoR I* and *Xho I*. We used the Bibiserv2 website to predict the binding site sequences between miR-301a-3p and *CHAC1* and *lncMDP1* (https://bibiserv.cebitec.uni-bielefeld.de/genefisher2/), which in turn led to the construction of the psiCHECKTM-2 dual luciferase reporter vector (Promega, Wisconsin, USA)^51^. The wild-type sequences of *CHAC1* 3’UTR and *lncMDP1* amplified from AA broiler leg muscle cDNA were ligated to the psiCHECK TM-2 vector using *Xho I* and *Not I* restriction enzyme sites. We changed the binding site of miR-301a-5p from TTGTCAG to GGTCTCT to generate mutant sequences of *CHAC1* 3’UTR and *lncMDP1*. *CHAC1*-WT or *CHAC1*-MT reporter plasmids and *lncMDP1*-WT or *lncMDP1*-MT reporter plasmids, combined with NC mimics or miR-301a-5p mimics were cotransfected into DF-1 cells (American Type Culture Collection, Virginia, USA) to study the miR-301a-5p binding of *CHAC1* 3’UTR and *lncMDP1* sites^52^. Then, 36 h post-transfection, the cells were treated according to the manufacturer’s instructions, and firefly and renilla luciferase activities were assayed using a dual luciferase reporter system (Promega, Wisconsin, USA).

### Flow cytometry, CCK-8, and EdU assays

CCK-8 analysis: Cell viability was determined using a CCK-8 kit (Vazyme, Nanjing, China). CPMs cultured in 96-well plates were assayed for cell viability at 450 nm every 12 h for two days using a fluorescence multimode digest (BD BioTek, Winooski, USA). The cell plates were incubated at 37 °C in a 5% CO_2_ incubator after adding 10 μL of CCK-8 solution to each well 2 h before the assay according to the manufacturer’s instructions.

EdU assay: We transfected CPMs inoculated in 24-well plates and fixed the cells after 36 h. The cells were incubated for 2 h with the Cell-Light EdU Apollo 567 in vitro kit (RiboBio, Guangzhou, China) according to the manufacturer’s instructions^53^. The images were acquired using a fluorescent microscope (Olympus, Tokyo, Japan). All experiments were repeated three times.

Flow cytometry: We performed transfection of CPMs inoculated in 6-well plates confluent to 70% (pcDNA3.1-3xFLAG) and 40% (siRNA), respectively. Cells were observed to fuse to 90–100% when cells were processed, first washed with PBS, digested with trypsin, and terminated with complete medium to obtain cell precipitates and then resuspended with 70% ethanol and subsequently placed at -4 °C to fix the cells. Cell cycle analysis we were performed using a BD Accuri C6 flow cytometer (BD Biosciences, California, USA).

### Immunofluorescence

We performed MyHC immunofluorescence detection for CPMs inoculated in 12-well plates by co-incubation with anti-MyHC antibody (DHSB, USA; B103) and anti-mouse Cy3-coupled antibody (Proteintech, Wuhan, China), and nuclear staining was performed using DAPI (Solarbio, Beijing, China). Images were still acquired using a fluorescence microscope (Olympus, Tokyo, Japan).

### RNA-FISH

CPMs were inoculated in 6-well plates, slides were placed in the cell culture plates in advance, and cells were fixed using in situ hybridization fixative when they reached 80–90% confluence. After the cells were then permeabilized with 0.1% Triton X-100, the cells were incubated with *lncMDP1* and *CHAC1* probes (Servicebio, Wuhan, China) respectively at 37 °C for 12 h. The nuclei were stained using DAPI and observed and photographed by laser confocal microscopy (Nikon, Tokyo, Japan).

### Western blotting assay

We lysed the CPMs inoculated in 6-well plates in lysis buffer (EpiZyme, Shanghai, China). Cellular protein concentration was determined by the BCA protein assay kit method (A53225, Thermo, Waltham, USA). Protein denaturation was subsequently performed by using 5 × loading buffer at 100 °C for 10 min after protein denaturation, and the denatured samples could be directly used for protein blotting analysis. We first separated the total cellular proteins using 12% SDS-PAGE and then transferred them to PVDF membranes (Millipore, MA, USA), followed by sealing the membranes with 5% skim milk in Tris-buffered saline containing 0.5% Tween-20 for 1 h on a decolorization shaker, followed by the use of anti-MYHC (1:400, B103; DHSB, Iowa City, USA), anti-CDK1 (1:1000, 19532-1-AP, Proteintech, Wuhan, China), anti-CHAC1 (1:1000, 15207-1-AP; Proteintech, Wuhan, China), anti-Desmin (1:5000, 16520-1-AP; Proteintech, Wuhan, China), and anti-GAPDH (1:10000, 60004-1-Ig; Proteintech, Wuhan, China) were incubated overnight at 4 °C, and the next day secondary antibodies coupled with HRP (1:2000, SA00001-1; Proteintech, Wuhan, China) were placed at room temperature for 1 h. Finally, the membranes were incubated by Images obtained by Odyssey FC (LI-COR, Nebraska, USA) and the signal was enhanced using ECL solution (EpiZyme, Shanghai, China) before taking pictures. GAPDH was used to normalize protein expression^52^. Each experiment is guaranteed to have three biological replicates. The grayscale values of each band were calculated using ImageJ software (NIH, Bethesda, USA). In this study, western blot was performed on different membranes for proteins with a molecular weight difference of fewer than 5 kDa (similar protein size, difficult to distinguish), and the sample size was consistent under the premise of detecting protein concentration. In this case, the sample and the loading control ran on different gels and thus transferred to different membranes. In addition, for proteins with a molecular weight difference greater than 5 kDa (similar protein size, difficult to distinguish), the loading amount and the loading control ran on the same gel, transferring to different membranes.

### Skeletal muscle injury and regeneration

Muscle injury was induced by injecting 50 μL of 50 mM BaCl_2_ in saline into the GAS muscle of 3-week-old AA males^54^. After the injury, 6 × 10^6^ plaque-forming unit (PFU) of pAV-*CHAC1*-3xFLAG and pAV-3xFLAG as control adenovirus were injected into the GAS muscle, and the GAS muscle was collected on days 1, 3, 5, and 7 of adenovirus injection for subsequent muscle regeneration assays. Our method of euthanasia for chickens is cervical dislocation.

### Statistics and reproducibility

All data in this study were statistically analyzed using SPSS software (SPSS for Windows, standard version 24.0; SPSS, New York, USA). Whether the values were statistically significantly different between the different two groups (*P < 0.05; **P < 0.01, ***P < 0.001) was statistically analyzed by using the *t* test, all experiments included at least three biological replicates, and the data were expressed as mean ± S.E.M. The source data behind the graphs in the figure were in supplementary data 1. The statistical results of relevant data in the article were in supplementary data 2.

### Reporting summary

Further information on research design is available in the Nature Portfolio Reporting Summary linked to this article.

### Supplementary information

Supplementary Information

Description of Additional Supplementary Files

Supplementary Data 1

Supplementary Data 2

Reporting Summary

## Data Availability

All study data results are available from the text. Whole transcriptome sequencing results have been uploaded to the NCBI Database Sequence Read Archive under the login numbers PRJNA909444 and PRJNA908949. All other data are available from the corresponding author on reasonable request. The source data and the statistical data can be found in the Supplementary Data 1 and 2.
