# Supplementary material for: Whole transcriptome profiling reveals a lncMDP1 that regulates myogenesis by adsorbing miR-301a-5p targeting CHAC1
Source: Commun Biol. 2024 May 2;7:518. doi: 10.1038/s42003-024-06226-1 (PMC11066001; doi:10.1038/s42003-024-06226-1)
Supplement: Supplementary file 1 — Supplementary Information [file 42003_2024_6226_MOESM1_ESM.pdf]

Title:

Whole transcriptome profiling reveals a *lncMDP1* that regulates myogenesis by adsorbing miR-301a-5p targeting *CHAC1*

Authors:

Bingjie Chen[1,*], Hanfang Cai1[,*], Yufang Niu[1], Yushi Zhang[1], Yanxing Wang[1], Yang Liu[1], Ruili Han[1,2], Xiaojun Liu[1,2], Xiangtao Kang[1,2,#] and Zhuanjian Li[1,2,#]

[1]College of Animal Science and Technology, Henan Agricultural University, Zhengzhou 450046, China;

[2]Henan Key Laboratory for Innovation and Utilization of Chicken Germplasm Resources, Zhengzhou 450046, China.

[*]These authors contributed equally to this work.

Corresponding author: Zhuanjian Li. Email: lizhuanjian@henau.edu.cn; Xiangtao Kang. Email: xtkang2001@263.net

Supplementary Information

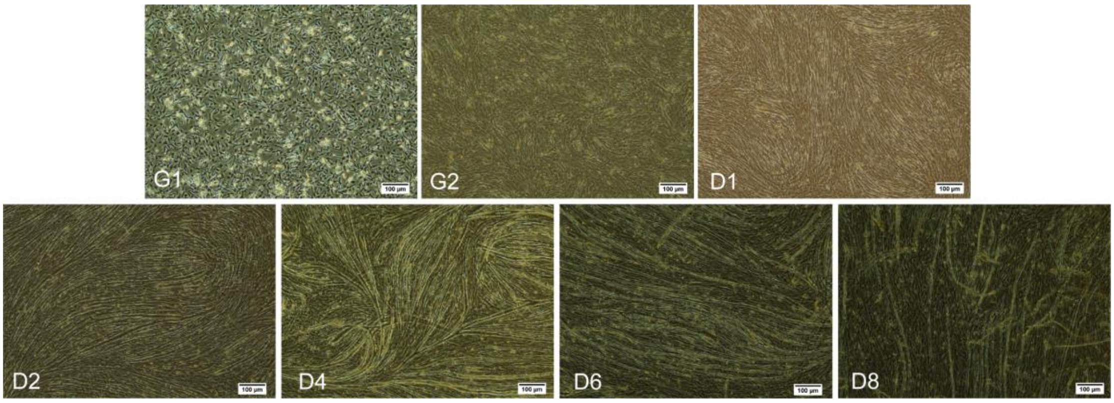

Supplementary Figure 1 Proliferation and differentiation stage model of chicken primary myoblasts. Cell culture images of CPMs during the proliferation (G1 and G2 represent 50% and 100% confluence.) and differentiation (D1, D2, D4, D6, and D8) periods. The scale is 100 μm.

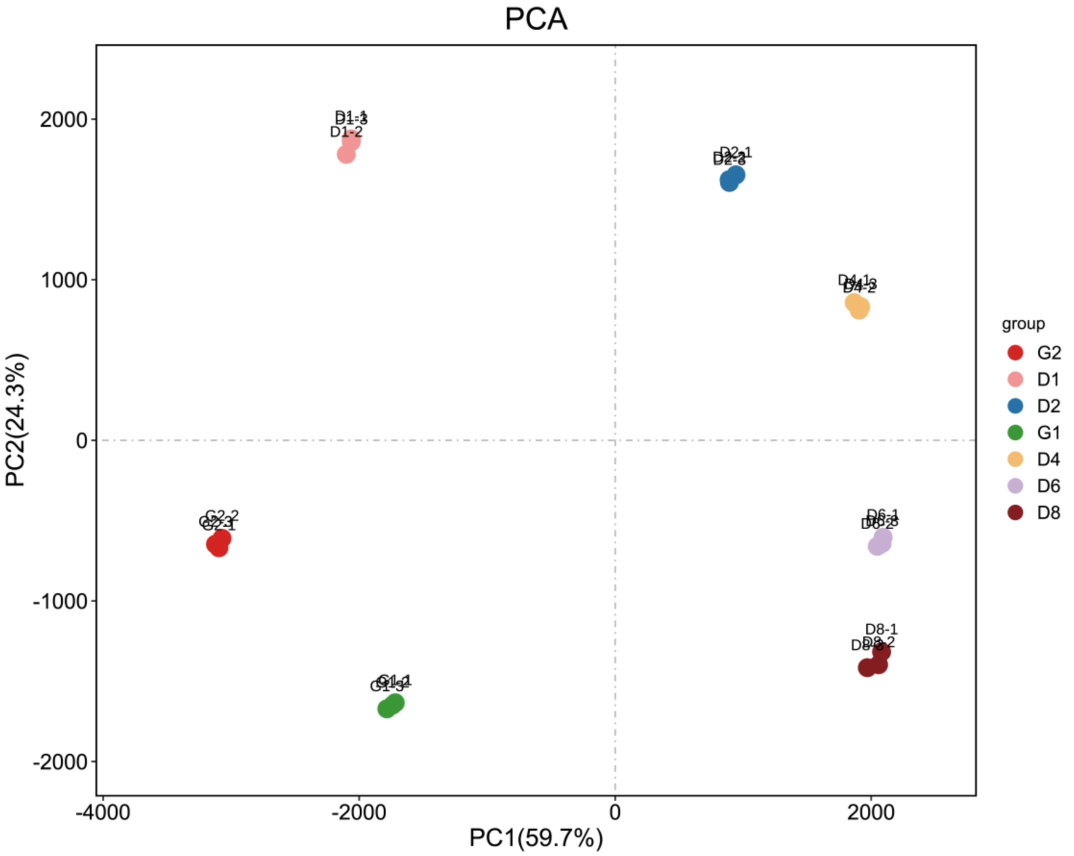

Supplementary Figure 2 PCA was used to understand the repeatability of samples from different developmental stages of CPMs.

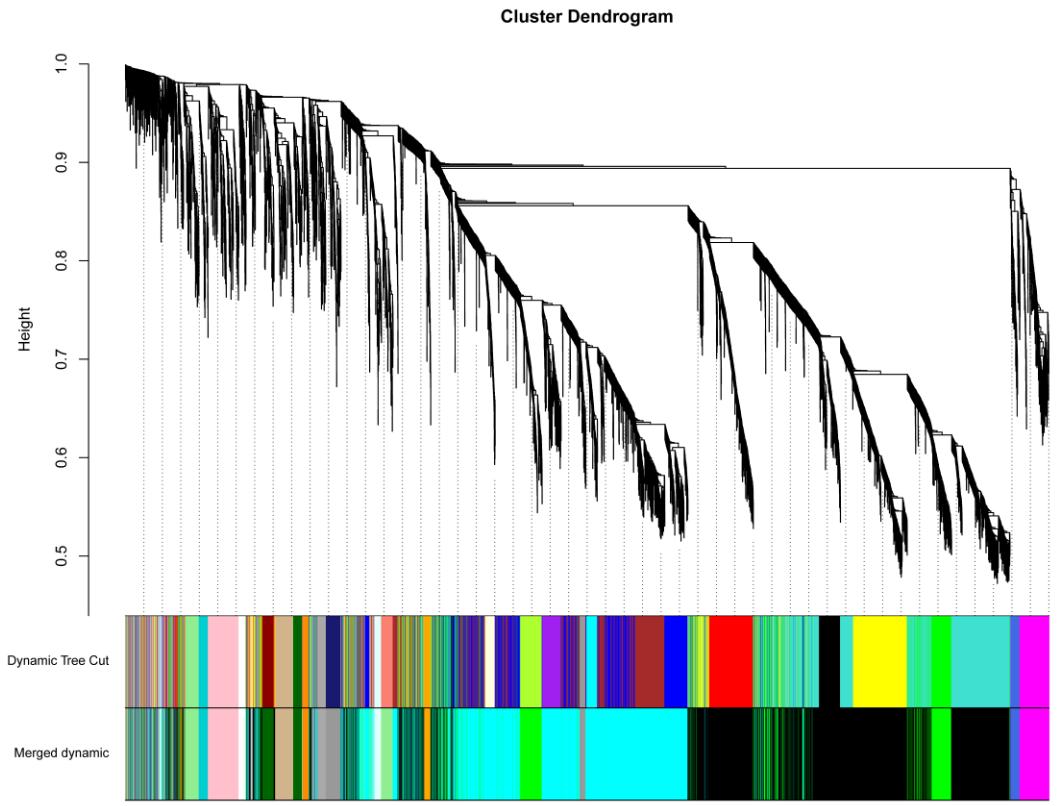

Supplementary Figuree 3 Hierarchical clustering tree showing the coexpression modules identified by WGCNA. Each leaf in the tree represents a gene. The major branches of the tree constitute 17 modules labeled with different colors.

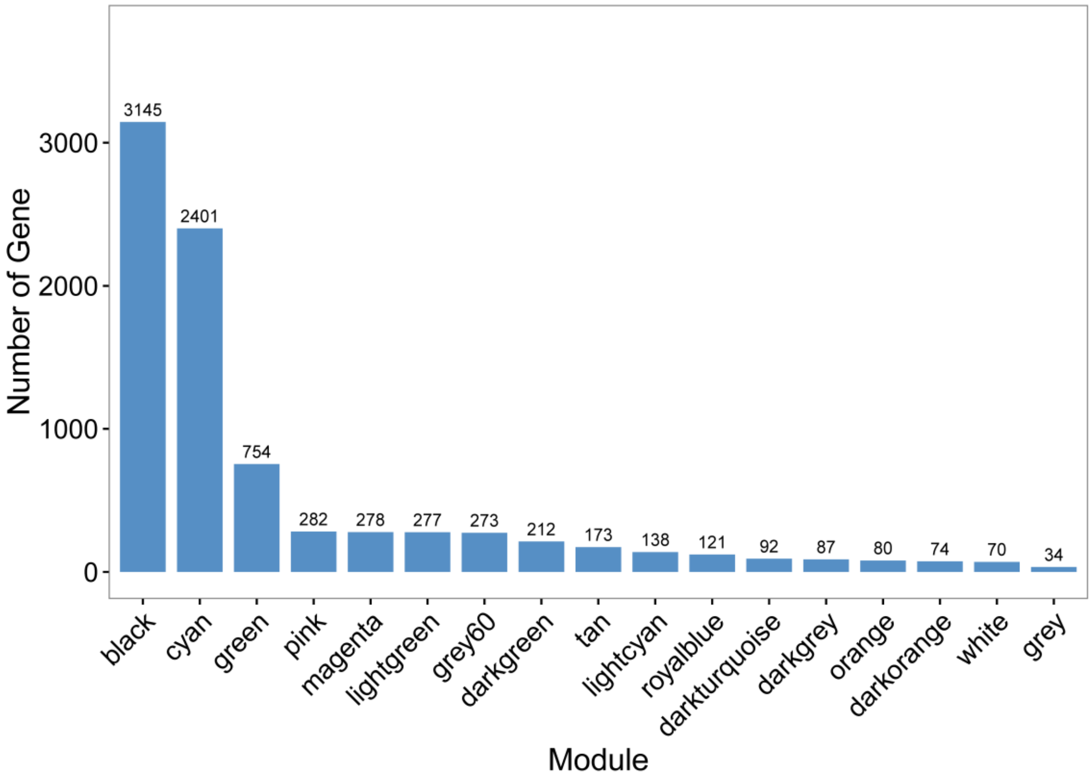

Supplementary Figure 4 Number of genes contained in each module.

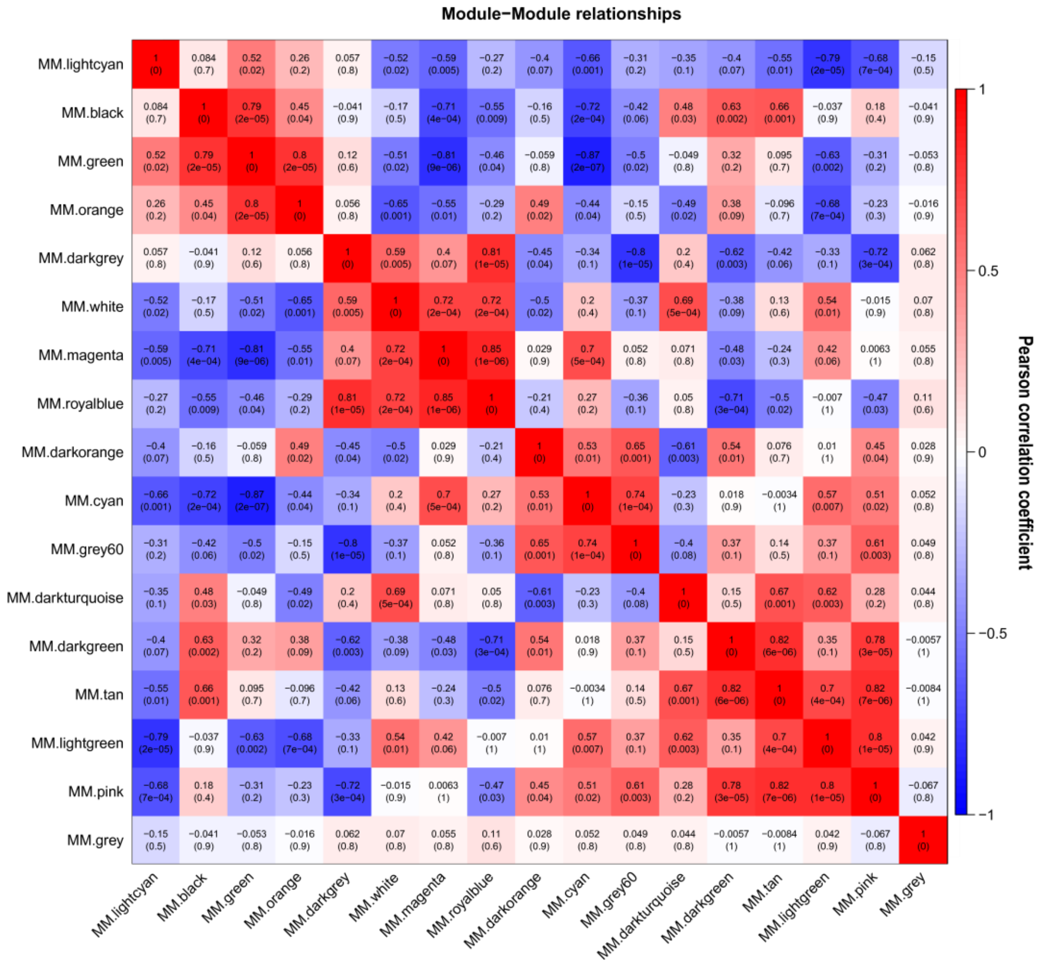

Supplementary Figure 5 Correlation analysis between modules. The deeper the color of the cube (redder or bluer), the stronger the correlation. The lighter the color of the cube, the weaker the correlation.

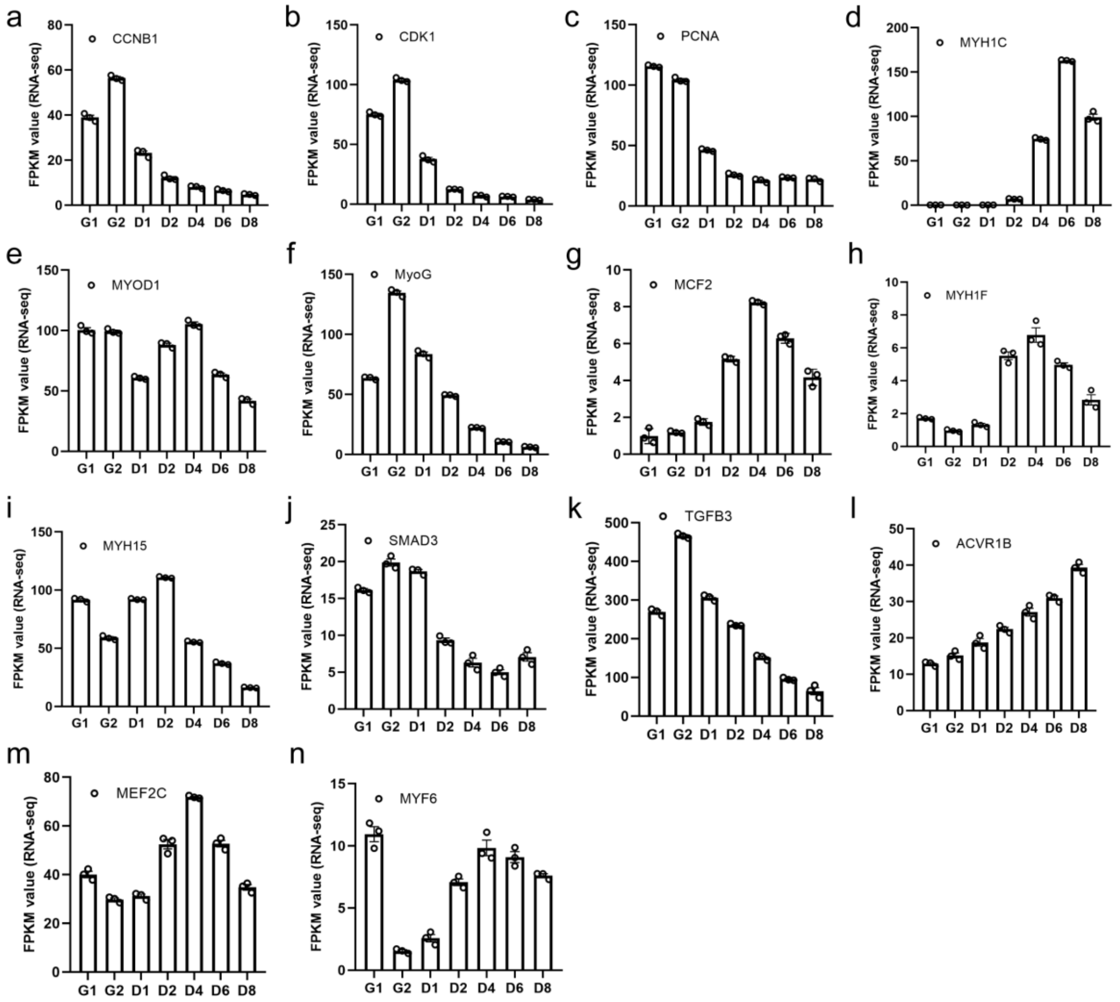

Supplementary Figure 6 RNA-seq of 14 marker genes related to myoblast proliferation (*CDK1*, *CCNB1*, *PCNA*) (**a-b**), differentiation (*MYH1C*, *MYOD1*, *MyoG*, *MCF2*, *MYH1F*, *MYH15*) (**d-i**), fusion (*SMAD3*, *TGFB3*, *ACVR1B*) (**j-l**), myoblast-related (*MEF2C*, *MYF6*) (**m-n**). The results are shown as the mean ± SEM of three independent experiments.

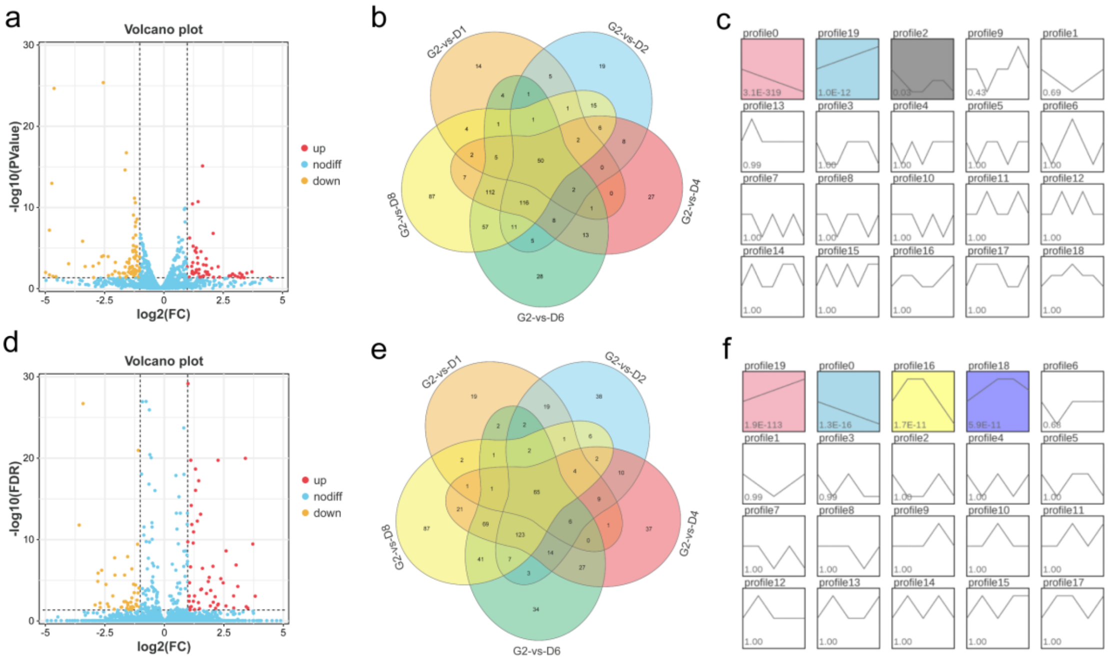

Supplementary Figure 7 Overview of the DEmiRNAs and DElncRNAs. a Volcanic graphs of differentially expressed miRNAs in G1-vs.-G2. The red scatter indicates up-regulated differentially expressed genes, the red scatter indicates down-regulated differentially expressed genes, and the blue scatter indicates no differentially expressed genes. **b** Venn diagram for the comparison of five groups: G2 and D1, D2, D4, D6, and D8. **c** Trend analysis plots DEmiRNAs. Pink represents profile0, blue represents profile19, gray represents profile2. **e** Volcanic graphs of differentially expressed miRNAs in G1-vs.-G2. **f** Venn diagram for the comparison of five groups: G2 and D1, D2, D4, D6, and D8. (G) Trend analysis plots of all DElncRNAs. Pink represents profile19, blue represents profile0, yellow represents profile16, purple represents profile18.

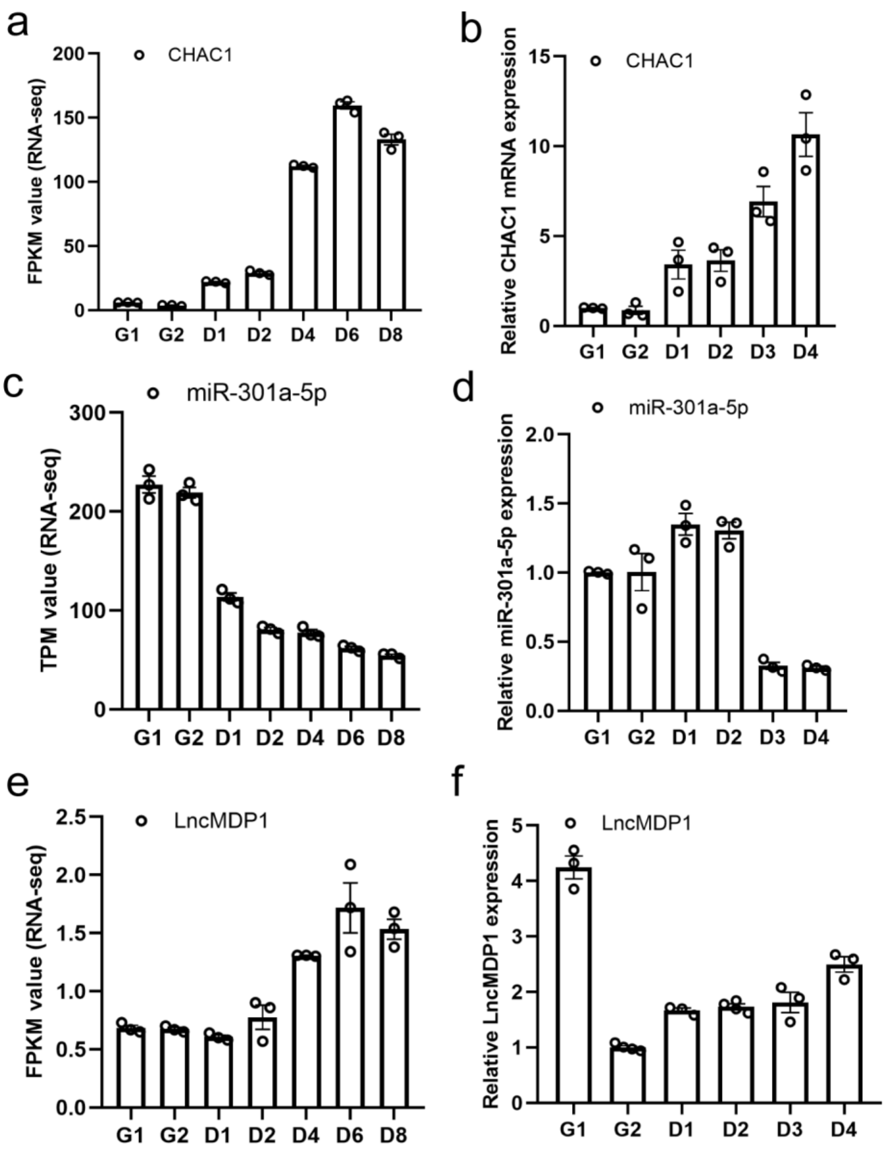

Supplementary Figure 8 Transcriptome data validation of *lncMPD3*–miR-301a-5p–*CHAC1* interaction network. a RNA sequencing results showed that *CHAC1* expression increased during myoblast differentiation, and the results were verified by qPCR. **b** RNA sequencing results showed that *lncMPD3* presented an overall upward trend during myoblast differentiation, and the results were verified by qPCR. **c** RNA sequencing results presented that miR-301a-5p showed a downward trend during myoblast differentiation, and the results were consistent with qPCR verification. The results are shown as the mean ± SEM of three independent experiments.

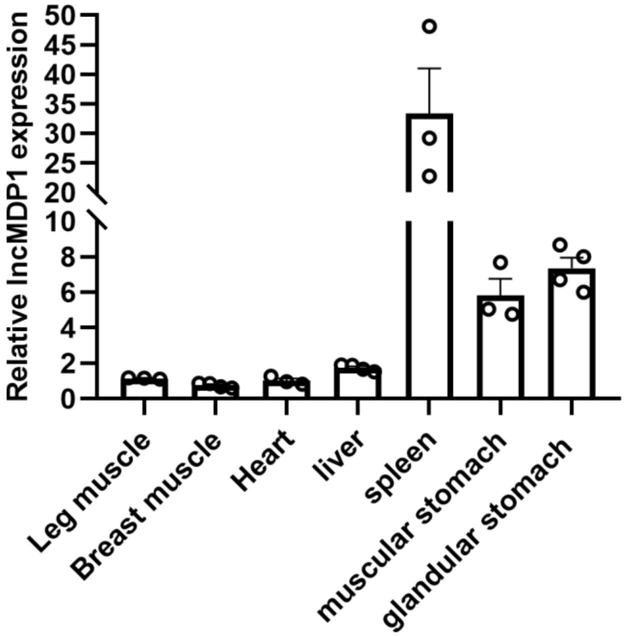

Supplementary Figure 9 RNA expression levels of *lncMPD3* in 7 different tissues of AA broilers. The relative RNA expression of *lncMPD3* in different tissues was normalized to the that in leg muscle. The results are shown as the mean ± SEM of three independent experiments.

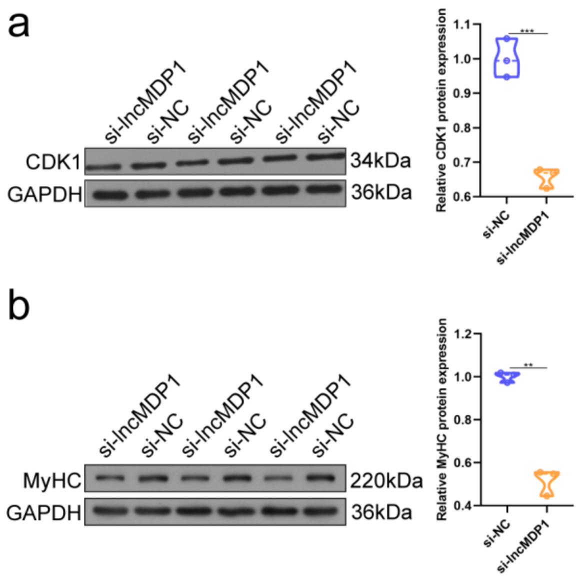

Supplementary Figure 10 Protein expression levels of CDK1 and MyHC after interference with *lncMDP1*. a Interference with *lncMDP1* decreased the protein expression level of CDK1. **b** Interference with *lncMDP1* reduced the protein expression levels of MyHC. The results are shown as the mean ± SEM of three independent experiments. The error bars are equivalent throughout the Figure. In the box line, a line in the middle of the box represents the median, and the upper and lower bottoms of the box are the upper quartile and the lower quartile, respectively. The blue circle represents si-NC, and the orange circle represents si-*lncMDP1*. (* $P < 0.05$; ** $P < 0.01$, *** $P < 0.001$)

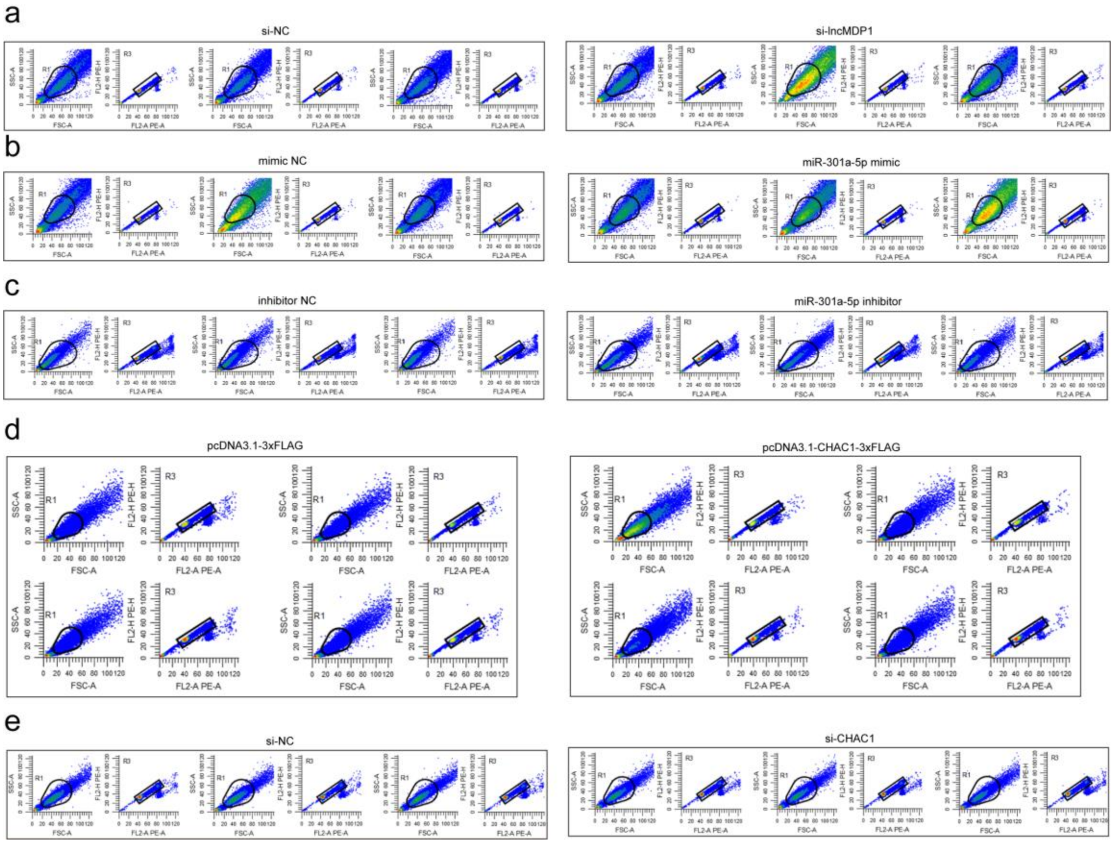

Supplementary Figure 11 Flow cytometry cycle detection gating strategy diagram. a Flow cytometry cycle gating strategy after interfering with si-*lncMDP1* and si-NC. **b** Flow cytometry cycle gating strategy after transfection of miR-301a-5p mimic and mimic NC. **c** Flow cytometry cycle gating strategy after transfection of miR-301a-5p inhibitor and inhibitor NC. **d** Flow cytometry cycle gating strategy of overexpressing pcDNA3.1-*CHAC1*-3xFLAG and pcDNA3.1-3xFLAG. **e** Flow cytometry cycle gating strategy after interfering with si-*CHAC1* and si-NC.

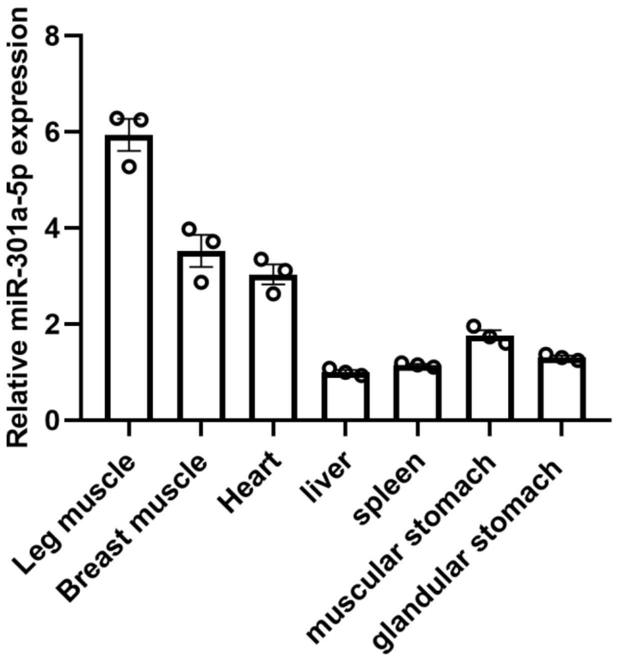

Supplementary Figure 12 RNA expression levels of miR-301a-5p in seven different tissues of AA broilers. The results are shown as the mean ± SEM of three independent experiments.

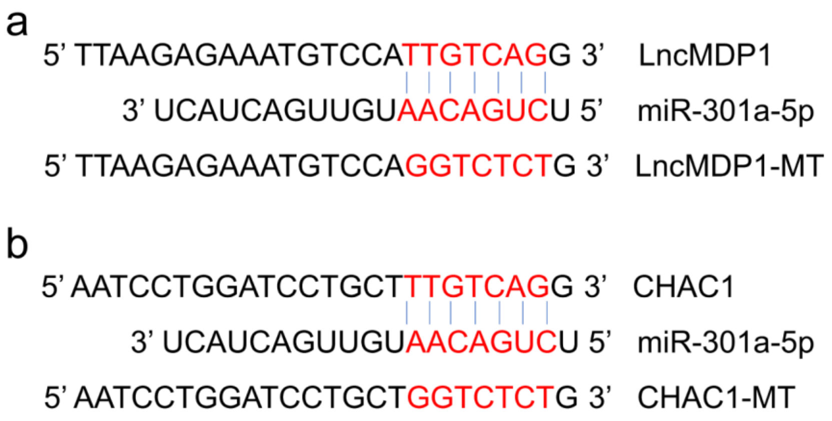

Supplementary Figure 13 Schematic representation of the binding sites of *lncMDP3* and *CHAC1* to miR-301a-5p. a Wild-type vector of *lncMPD3* (*lncMPD3*-WT) and the miR-301a-5p binding site sequences, and mutant vector of *lncMPD3* (*lncMPD3*-MT) with the miR-301a-5p binding site sequence. **b** Wild-type vector for *CHAC1* (*CHAC1*-WT) and the miR-301a-5p binding site sequence, and mutant vector for *CHAC1* (*CHAC1*-MT) with the miR-301a-5p binding site sequence. Red text represents the binding base sequence and mutation site.

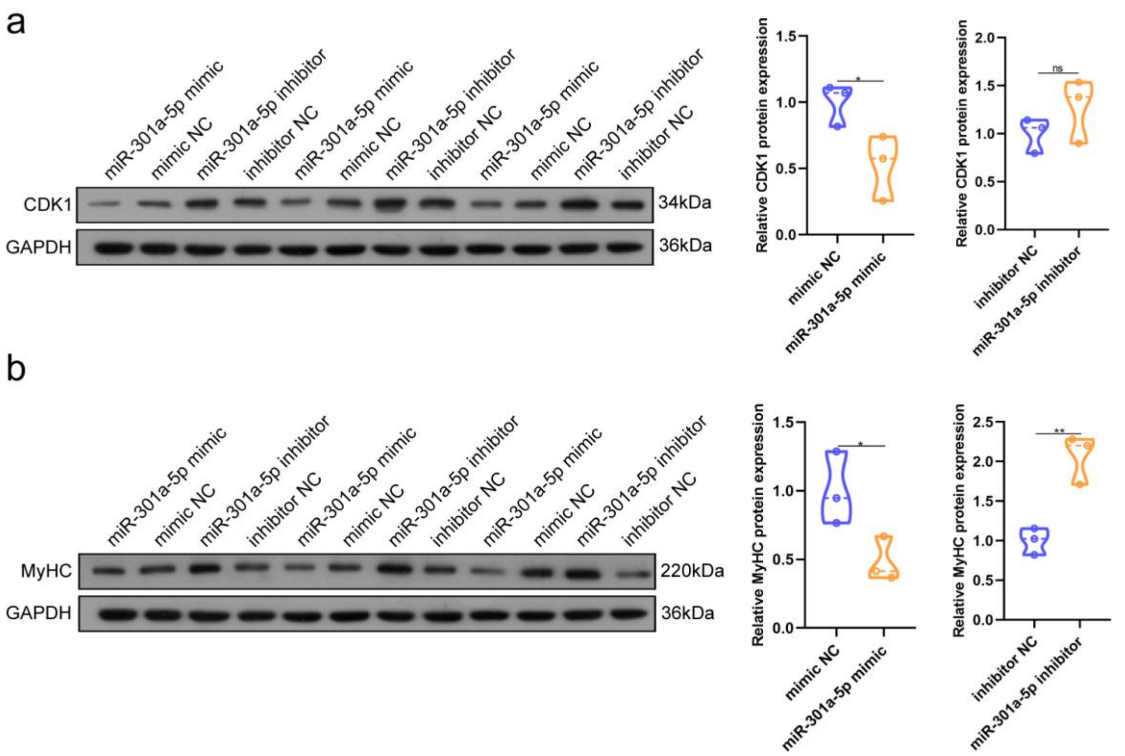

Supplementary Figure 14 Protein expression levels of CDK1 and MyHC after overexpression and interference with miR-301a-5p. a The protein expression level of CDK1 after transfection of miR-301a-5p mimics and miR-301a-5p inhibitors. Western blot was performed on different membranes for proteins with a molecular weight difference of fewer than 5 kDa (similar protein size, difficult to distinguish), and the sample size was consistent under the premise of detecting protein concentration. In this case, the sample and the loading control ran on different gels and thus transferred to different membranes. **b** The protein expression level of MyHC after transfection of miR-301a-5p mimics and miR-301a-5p inhibitors. The results are shown as the mean ± SEM of three independent experiments. The error bars are equivalent throughout the Figure. In the box line, a line in the middle of the box represents the median, and the upper and lower bottoms of the box are the upper quartile and the lower quartile, respectively. The blue circle of the left box line diagram represents mimic NC, and the orange square represents miR-301a-5p mimic. The blue circle of the right box line diagram represents inhibitor NC, and the orange square represents miR-301a-5p inhibitor. (* $P < 0.05$; ** $P < 0.01$, *** $P < 0.001$)

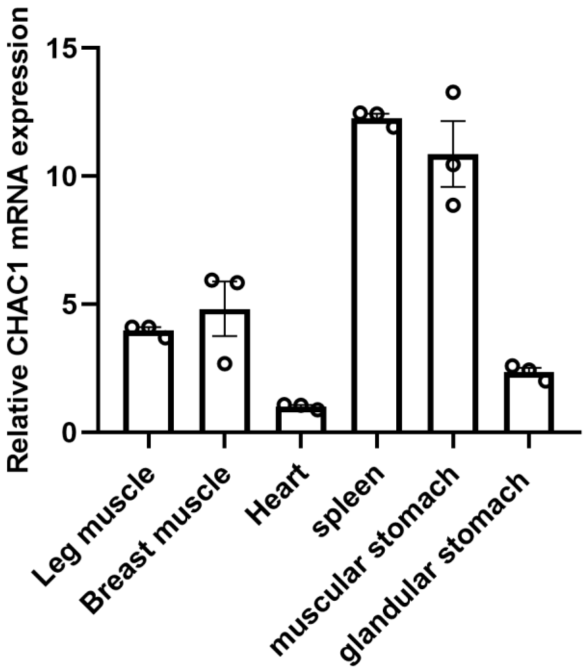

Supplementary Figure 15 RNA expression levels of *CHAC1* in seven different tissues of AA broilers. The results are shown as the mean ± SEM of three independent experiments.

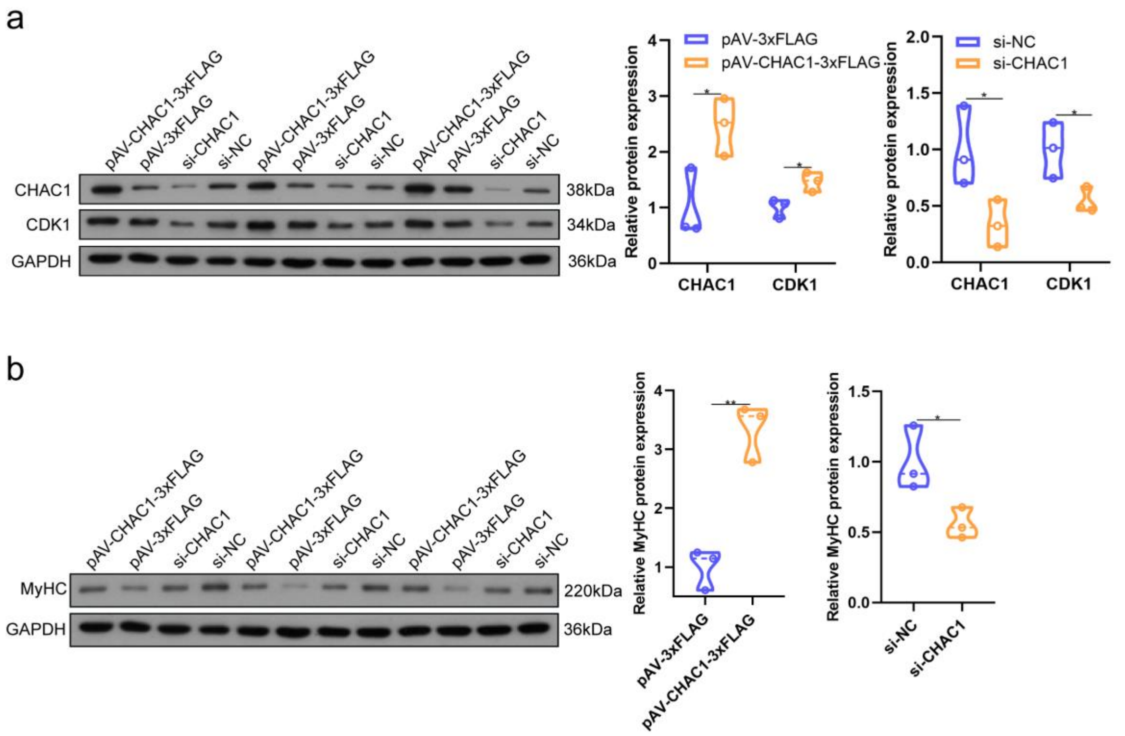

Supplementary Figure 16 ***CHAC1*** **promotes myoblast proliferation and differentiation. a** Protein expression levels of CDK1 and CHAC1 after *CHAC1* overexpression and interference. The error bars are equivalent throughout the Figure. In the box line, a line in the middle of the box represents the median, and the upper and lower bottoms of the box are the upper quartile and the lower quartile, respectively. The blue circle of the left box line diagram represents pAV-3xFLAG, and the orange circle represents pAV-*CHAC1*-3xFLAG. The blue circle of the right box line diagram represents si-NC, and the orange circle represents si-*CHAC1*. Western blot was performed on different membranes for proteins with a molecular weight difference of fewer than 5 kDa (similar protein size, difficult to distinguish), and the sample size was consistent under the premise of detecting protein concentration. In this case, the sample and the loading control ran on different gels and thus transferred to different membranes. **b** Protein expression levels of MyHC after *CHAC1* overexpression and interference. The results are shown as the mean ± SEM of three independent experiments. The error bars are equivalent throughout the Figure. In the box line, a line in the middle of the box represents the median, and the upper and lower bottoms of the box are the upper quartile and the lower quartile, respectively. The blue circle of the left box line diagram represents pAV-3xFLAG, and the orange circle represents pAV-*CHAC1*-3xFLAG. The blue circle of the right box line diagram represents si-NC, and the orange circle represents si-*CHAC1*. (* $P < 0.05$; ** $P < 0.01$, *** $P < 0.001$)

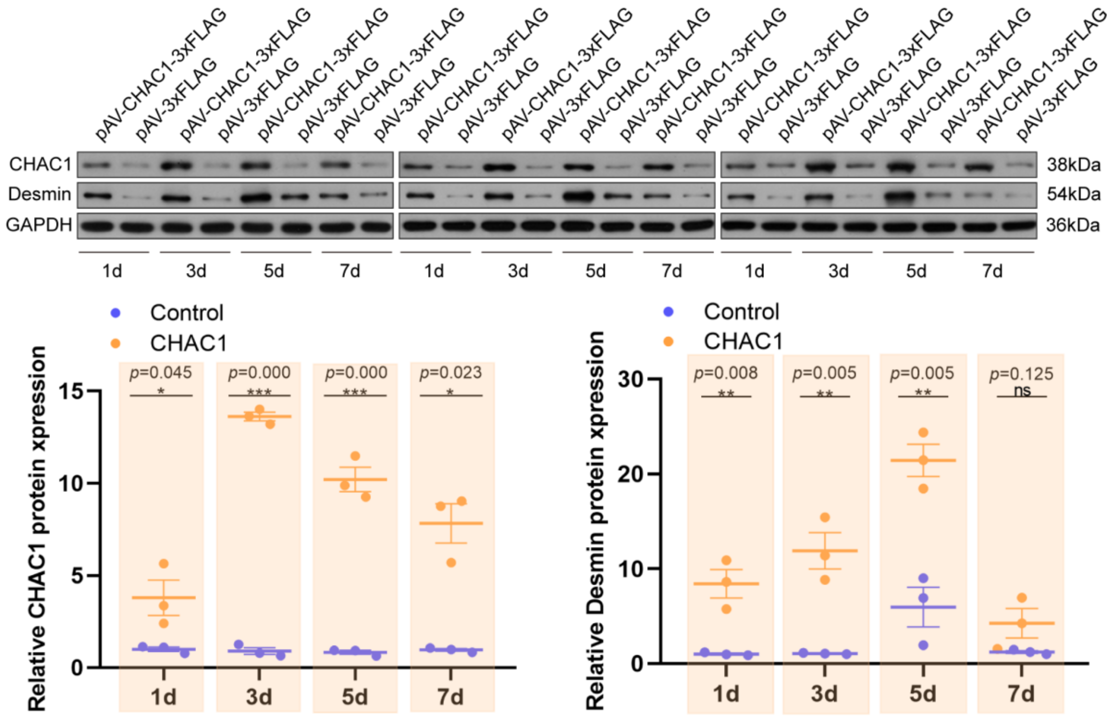

Supplementary Figure 17 Protein expression levels of CHAC1 and Desmin. The results are shown as the mean ± SEM of three independent experiments. The results are shown as the mean ± SEM of three independent experiments. Western blot was performed on different membranes for proteins with a molecular weight difference of fewer than 5 kDa (similar protein size, difficult to distinguish), and the sample size was consistent under the premise of detecting protein concentration. In this case, the sample and the loading control ran on different gels and thus transferred to different membranes. (* $P < 0.05$; ** $P < 0.01$, *** $P < 0.001$)

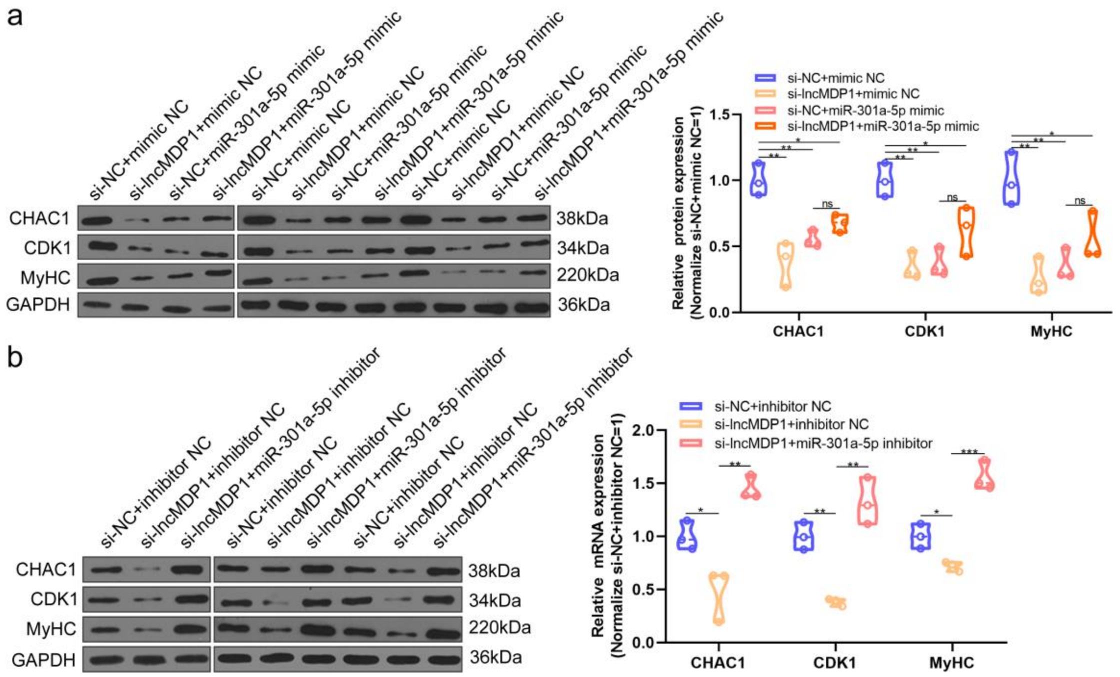

Supplementary Figure 18 ***LncMDP1*** **acts as a miR-301a-5p sponge attenuating its inhibitory effect on** ***CHAC1*****. a** Protein expression levels of *CHAC1* and proliferation and differentiation marker genes after cotransfection with si-*lncMDP1*, si-NC, miR-301a-5p mimic, and mimic NC. The error bars are equivalent throughout the Figure. In the box line, a line in the middle of the box represents the median, and the upper and lower bottoms of the box are the upper quartile and the lower quartile, respectively. The blue circle represents si-NC and mimic NC, the yellow circle represents si-lncMDP1 and mimic NC, the pink circle represents si-NC and miR-301a-5p mimic, and the orange circle represents si-lncMDP1 and miR-301a-5p mimic. . Western blot was performed on different membranes for proteins with a molecular weight difference of fewer than 5 kDa (similar protein size, difficult to distinguish), and the sample size was consistent under the premise of detecting protein concentration. In this case, the sample and the loading control ran on different gels and thus transferred to different membranes. **b** Protein expression levels of *CHAC1* and proliferation and differentiation marker genes after cotransfection with si-*lncMDP1*, si-NC, miR-301a-5p inhibitor, and inhibitor NC. The results are shown as the mean ± SEM of three independent experiments. The error bars are equivalent throughout the Figure. In the box line, a line in the middle of the box represents the median, and the upper and lower bottoms of the box are the upper quartile and the lower quartile, respectively. The blue circle represents si-NC and inhibitor NC, the yellow circle represents si-lncMDP1 and inhibitor NC, and the pink circle represents si-lncMDP1 and miR-301a-5p inhibitor. (* $P < 0.05$; ** $P < 0.01$, *** $P < 0.001$). Western blot was performed on different membranes for proteins with a molecular weight difference of fewer than 5 kDa (similar protein size, difficult to distinguish), and the sample size was consistent under the premise of detecting protein concentration. In this case, the sample and the loading control ran on different gels and thus transferred to different membranes.

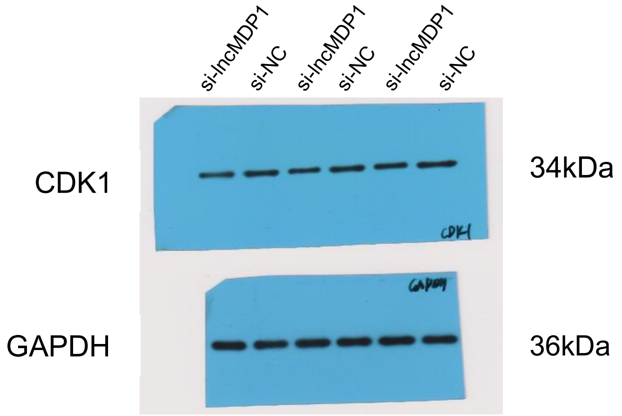

Supplementary Figure 19 The expression of CDK1 and GAPDH in si-*lncMDP1* and si-NC was shown in Figure 2e and Supplementary Figure 10a. Western blot was performed on different membranes for proteins with a molecular weight difference of fewer than 5 kDa (similar protein size, difficult to distinguish), and the sample size was consistent under the premise of detecting protein concentration. In this case, the sample and the loading control ran on different gels and thus transferred to different membranes.

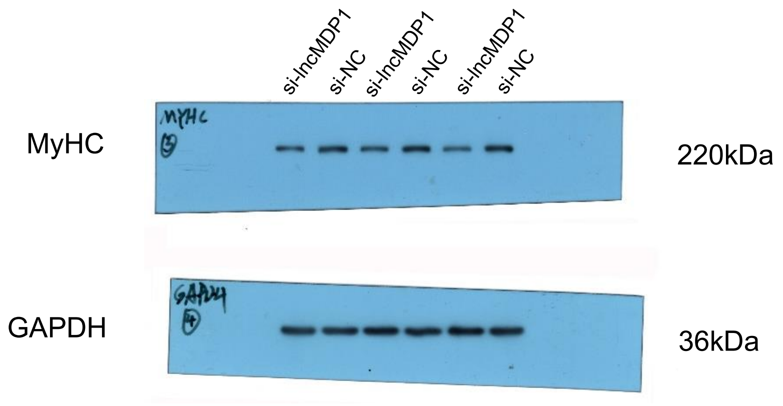

Supplementary Figure 20 The expression of MyHC and GAPDH in si-*lncMDP1* and si-NC was shown in Figure 2j and Supplementary Figure 10b.

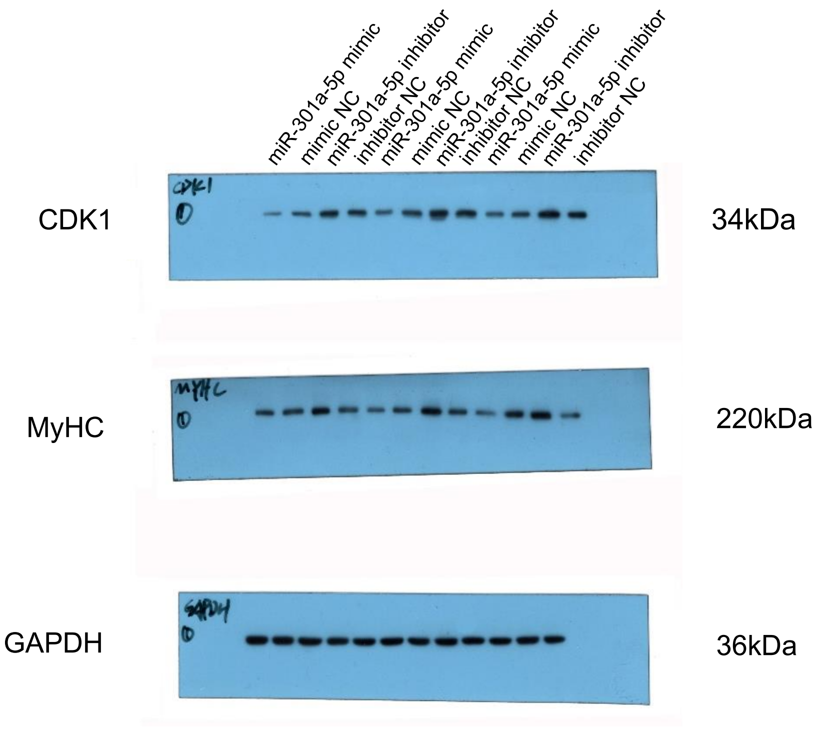

Supplementary Figure 21 The expression of CDK1, MyHC and GAPDH in miR-301a-5p mimic, mimic NC, miR-301a-5p inhibitor and inhibitor NC was shown in Figure 3g, Figure 3p, Supplementary Figure 14. Figures 3g and 3p were from different blots and since the molecular weight difference between CDK1 and GAPDH was only 2 kDa, it was not easy to distinguish between them, so the same internal control (GAPDH) was used to ensure that the sample load is consistent under the premise of detecting the protein concentration. Western blot was performed on different membranes for proteins with a molecular weight difference of fewer than 5 kDa (similar protein size, difficult to distinguish), and the sample size was consistent under the premise of detecting protein concentration. In this case, the sample and the loading control ran on different gels and thus transferred to different membranes.

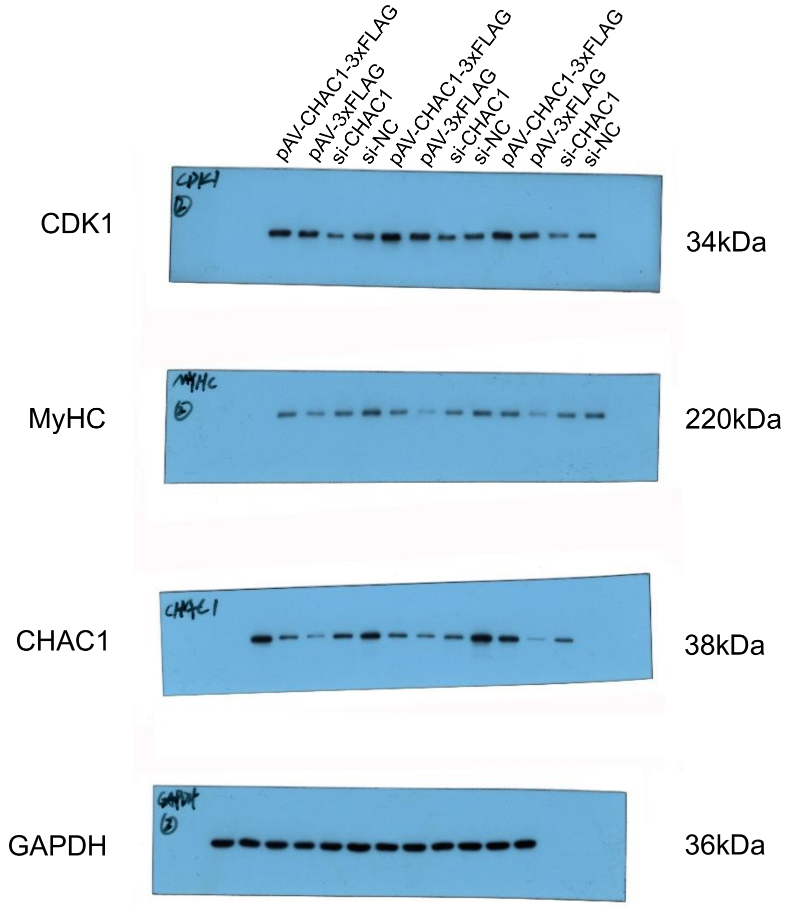

Supplementary Figure 22 The expression of CHAC1, CDK1, MyHC and GAPDH in pAV-*CHAC1*-3xFlag, pAV-3xFlag, si-*CHAC1* and si-NC was shown in Figure 4h, Figure 4q and Supplementary Figure 16. Figures 4h and 4q were from different blots. Since the molecular weight difference of GAPDH is 2 kDa from CDK1 and CHAC1, respectively, it was not easy to distinguish between them, so the same internal control (GAPDH) was used to ensure that the protein concentration was detected. Western blot was performed on different membranes for proteins with a molecular weight difference of fewer than 5 kDa (similar protein size, difficult to distinguish), and the sample size was consistent under the premise of detecting protein concentration. In this case, the sample and the loading control ran on different gels and thus transferred to different membranes.

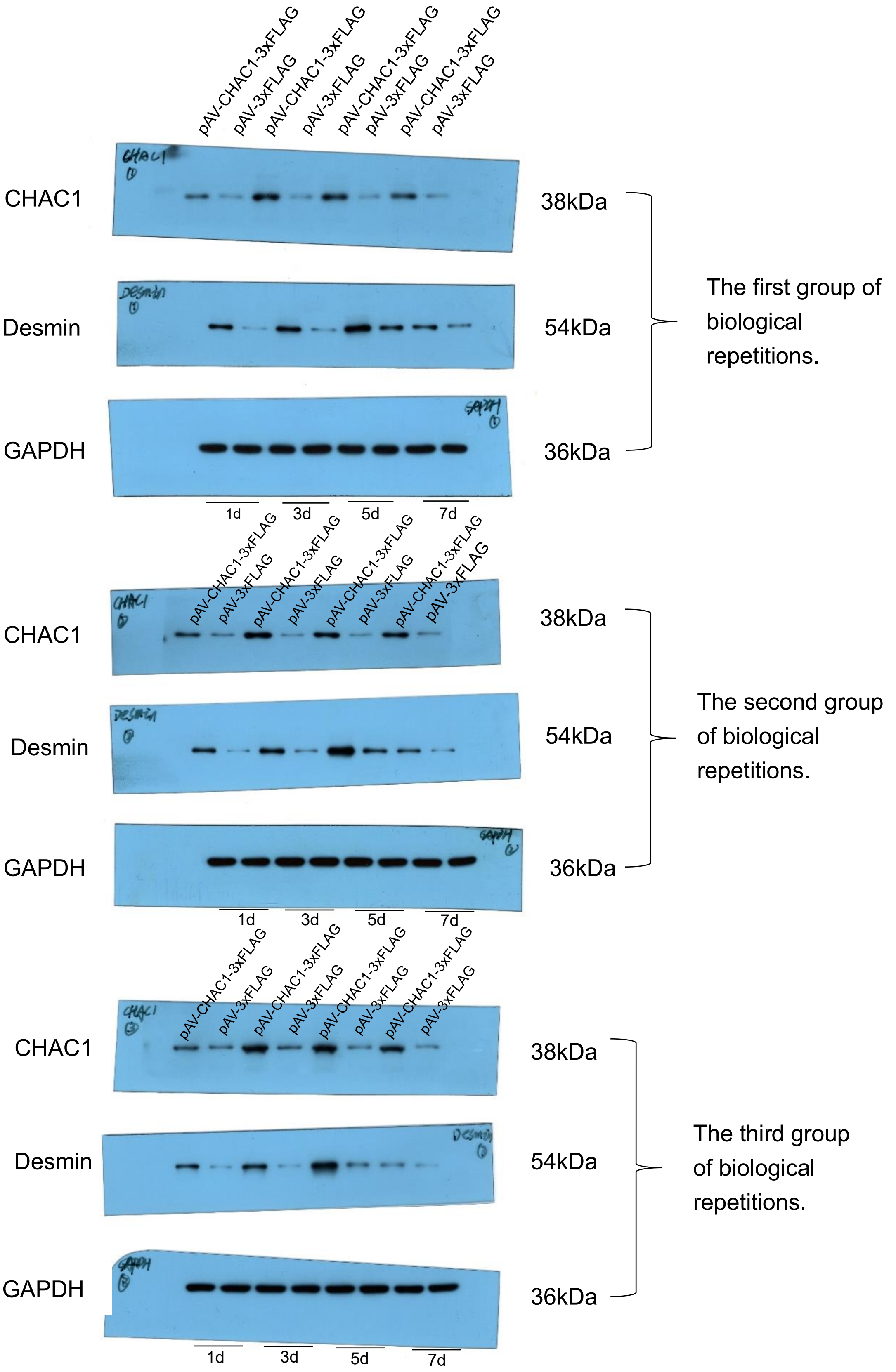

Supplementary Figure 23 The expression of CHAC1, Desmin and GAPDH in the gastrocnemius muscle of AA broilers injected with pAV-*CHAC1*-3xFlag and pAV-3xFlag is shown in Figure 5h (The 1st, 2nd and 3rd figures were from the same blot) and Supplementary Figure 17 (The 1st, 2nd and 3rd figures were from the same blot, the 4th, 5th and 6th figures were from the same blot, the 7th, 8th and 9th figures were from the same blot). Western blot was

performed on different membranes for proteins with a molecular weight difference of fewer than 5 kDa (similar protein size, difficult to distinguish), and the sample size was consistent under the premise of detecting protein concentration. In this case, the sample and the loading control ran on different gels and thus transferred to different membranes.

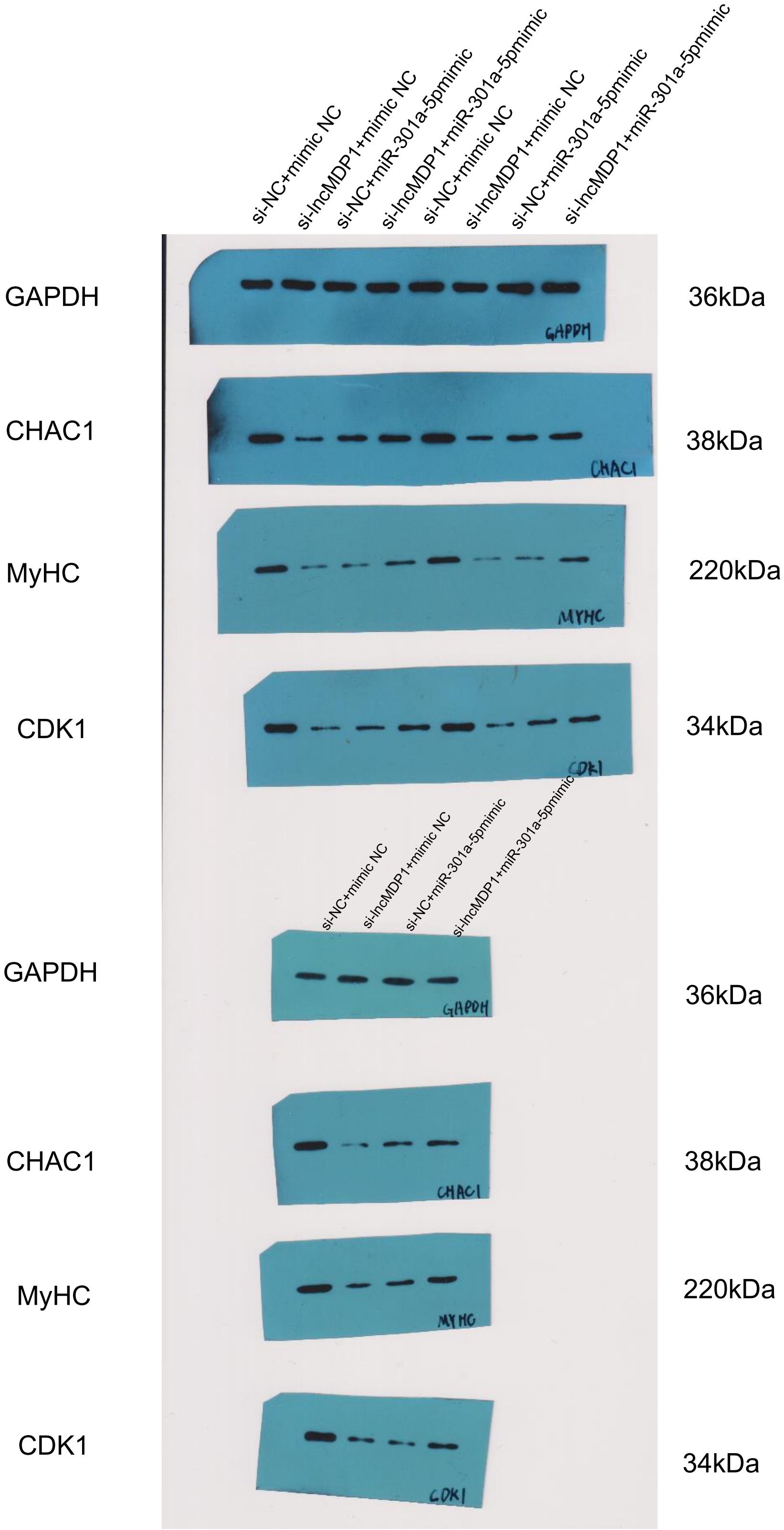

Supplementary Figure 24 The expression of CHAC1, CDK1, MyHC and GAPDH in si-*lncMDP1*, si-NC, miR-301a-5p mimic, mimic NC was shown in Figure 6d and Supplementary Figure 18a. Western blot was performed on different membranes for proteins with a molecular weight difference of fewer than 5 kDa (similar protein size, difficult to distinguish), and the sample size was consistent under the premise of detecting protein concentration. In this case, the sample

and the loading control ran on different gels and thus transferred to different membranes.

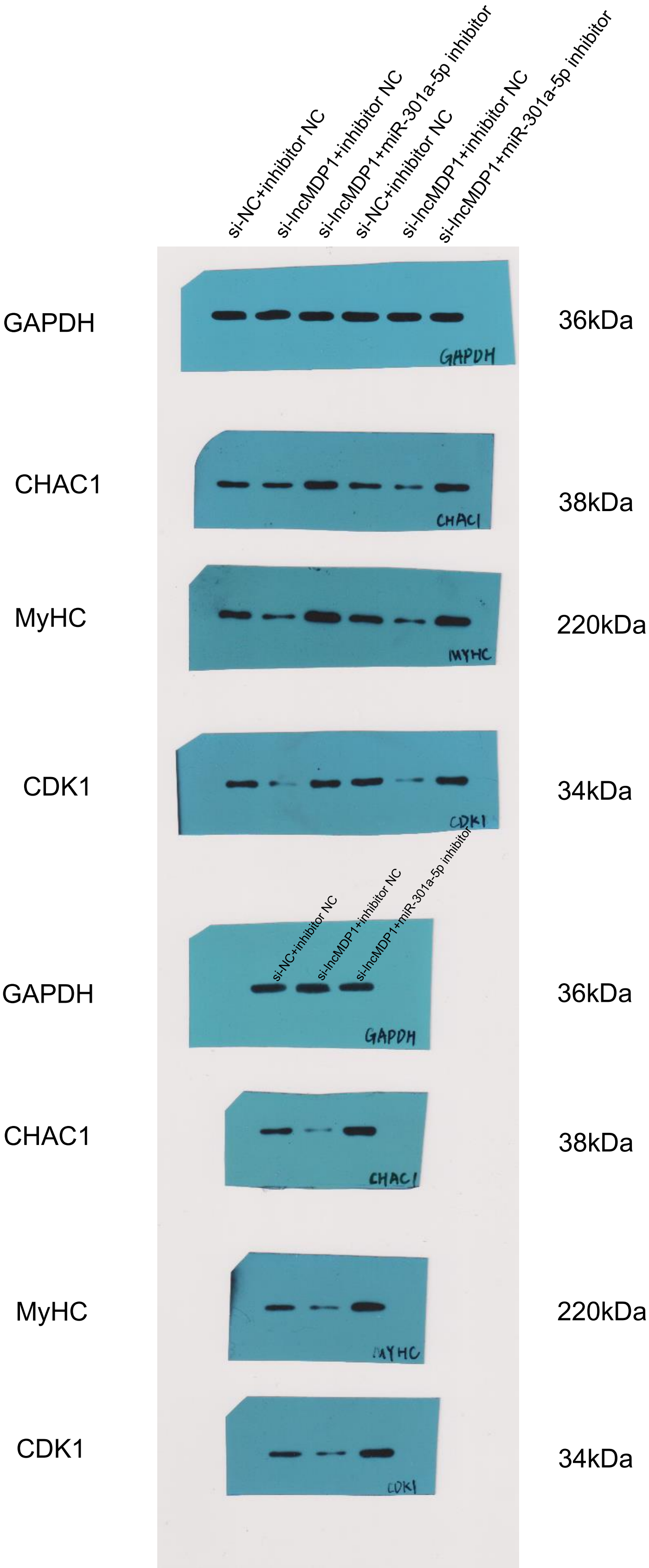

Supplementary Figure 25 The expression of CHAC1, CDK1, MyHC and GAPDH in si-*lncMDP1*, si-NC, miR-301a-5p inhibitor, inhibitor NC was shown in Figure 6h and Supplementary Figure 18b. Western blot was performed on different membranes for proteins with

a molecular weight difference of fewer than 5 kDa (similar protein size, difficult to distinguish), and the sample size was consistent under the premise of detecting protein concentration. In this case, the sample and the loading control ran on different gels and thus transferred to different membranes.

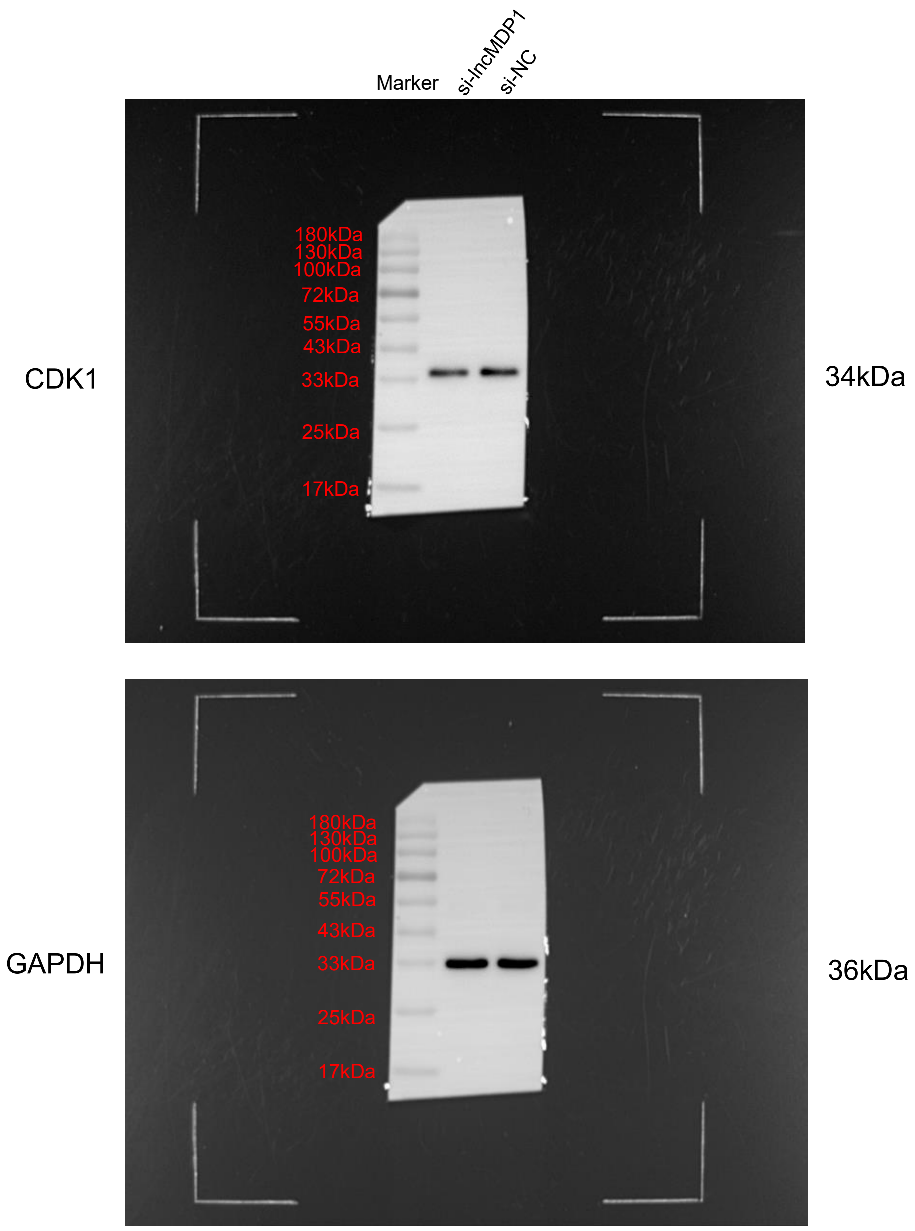

Supplementary Figure 26 The expression of CDK1 and GAPDH in si-*lncMDP1* and si-NC. Following the editing requirements, use a load-controlled repeat western blot (a set of biological replicates), run the target protein and load-control control on the same gel and transfer them to the same membrane, and finally provide an uncropped membrane. So we firstly incubate the membrane with antibodies to one of the CDK1 and GAPDH proteins, then wash the membrane to strip the proteins, and finally incubate the other protein on the membrane.

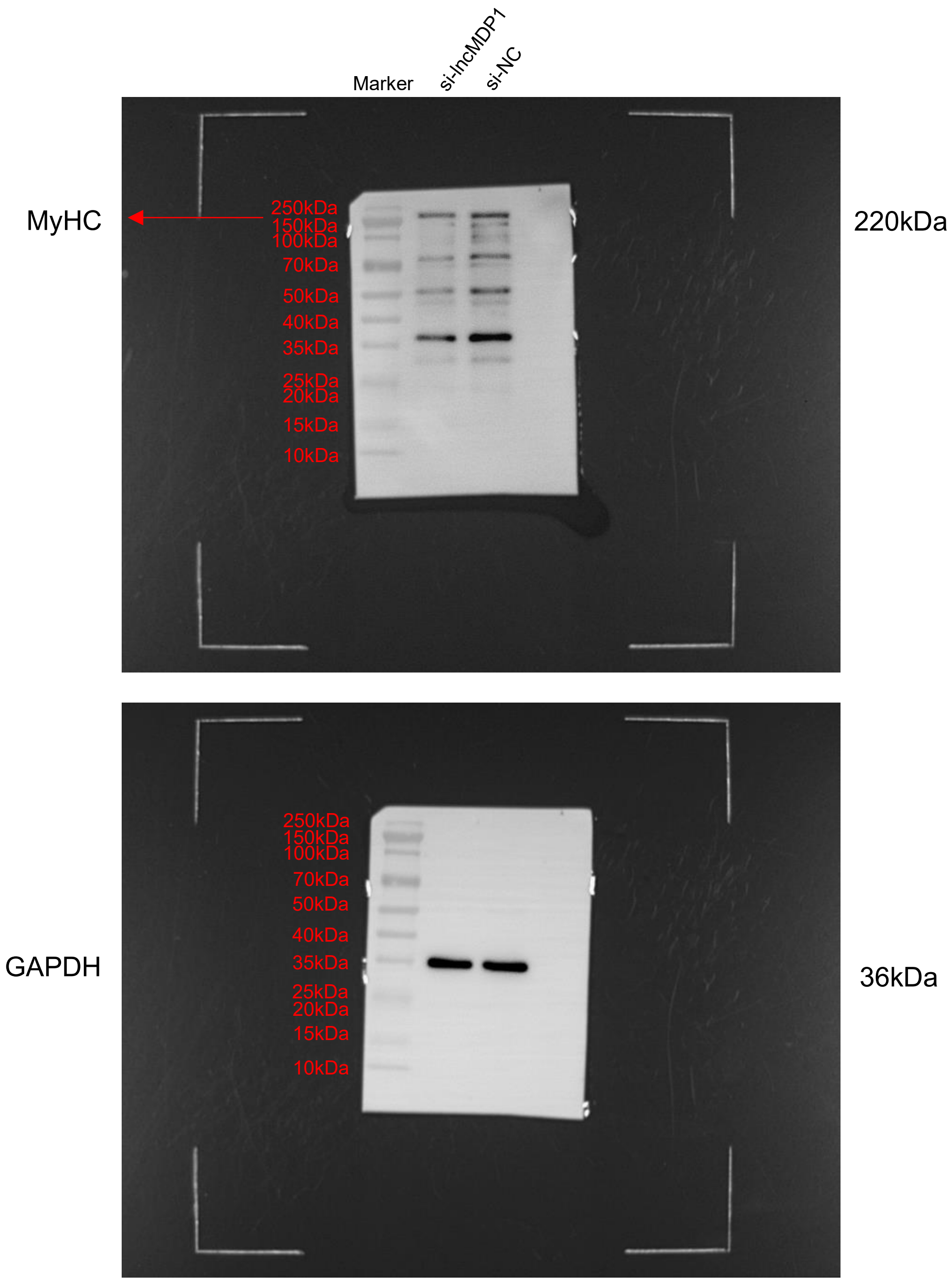

Supplementary Figure 27 The expression of MyHC and GAPDH in si-*lncMDP1* and si-NC. Following the editing requirements, use a load-controlled repeat western blot (a set of biological replicates), run the target protein and load-control control on the same gel and transfer them to the same membrane, and finally provide an uncropped membrane. So we firstly incubate the membrane with antibodies to one of the MyHC and GAPDH proteins, then wash the membrane to strip the proteins, and finally incubate the other protein on the membrane.

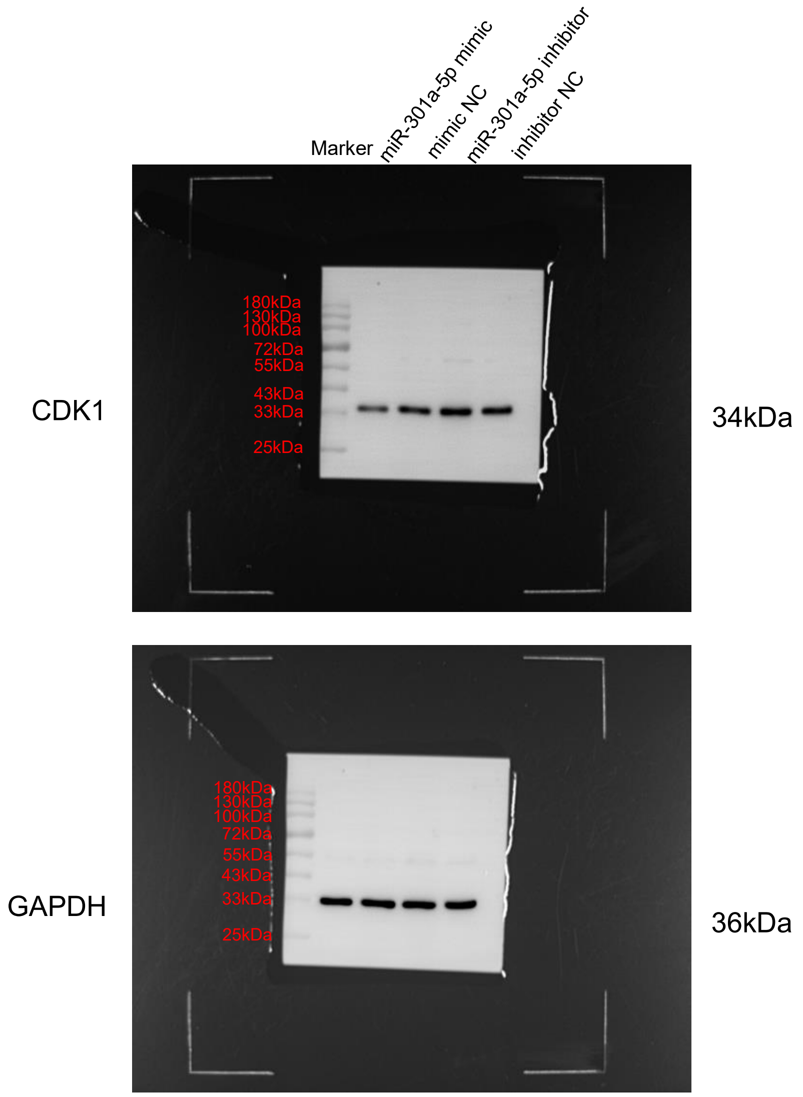

Supplementary Figure 28 The expression of CDK1 and GAPDH in miR-301a-5p mimic, mimic NC, miR-301a-5p inhibitor and inhibitor NC. Following the editing requirements, use a load-controlled repeat western blot (a set of biological replicates), run the target protein and load-control control on the same gel and transfer them to the same membrane, and finally provide an uncropped membrane. So we firstly incubate the membrane with antibodies to one of the CDK1 and GAPDH proteins, then wash the membrane to strip the proteins, and finally incubate the other protein on the membrane.

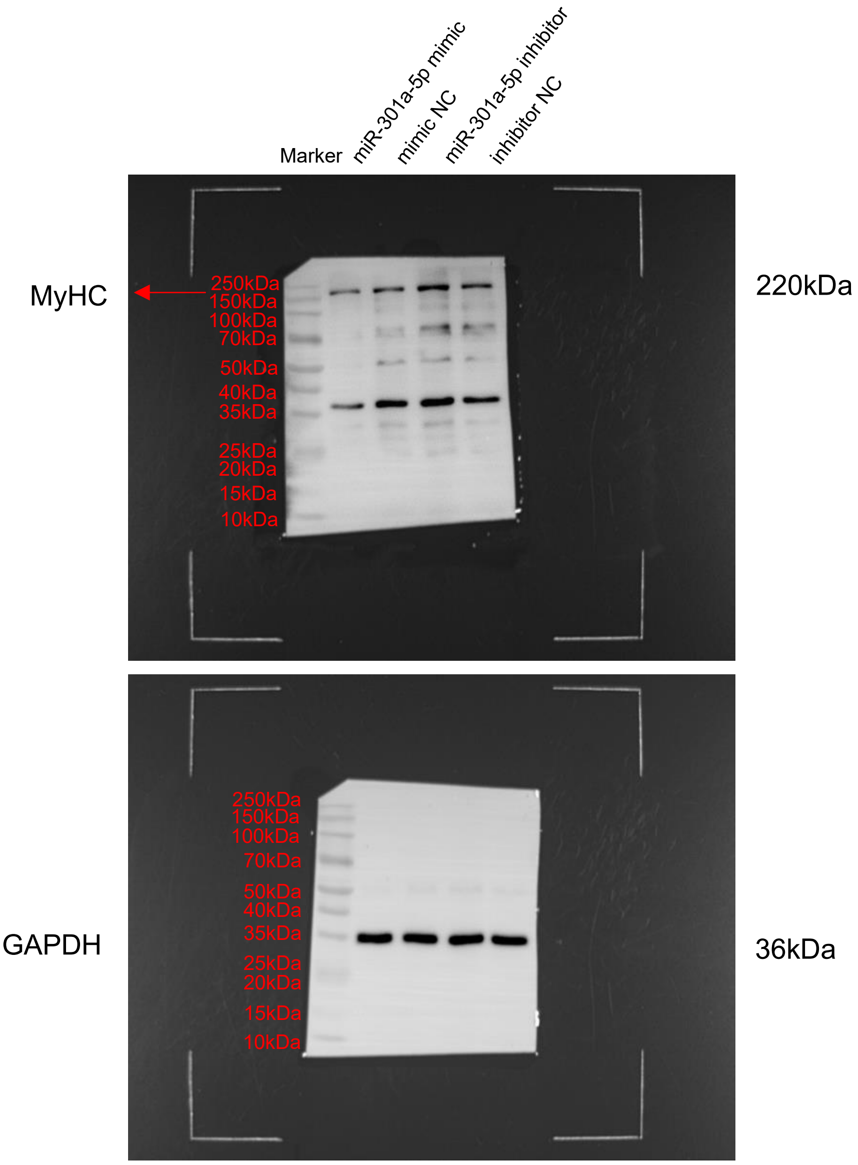

Supplementary Figure 29 The expression of MyHC and GAPDH in miR-301a-5p mimic, mimic NC, miR-301a-5p inhibitor and inhibitor NC. Following the editing requirements, use a load-controlled repeat western blot (a set of biological replicates), run the target protein and load-control control on the same gel and transfer them to the same membrane, and finally provide an uncropped membrane. So we firstly incubate the membrane with antibodies to one of the MyHC and GAPDH proteins, then wash the membrane to strip the proteins, and finally incubate the other protein on the membrane.

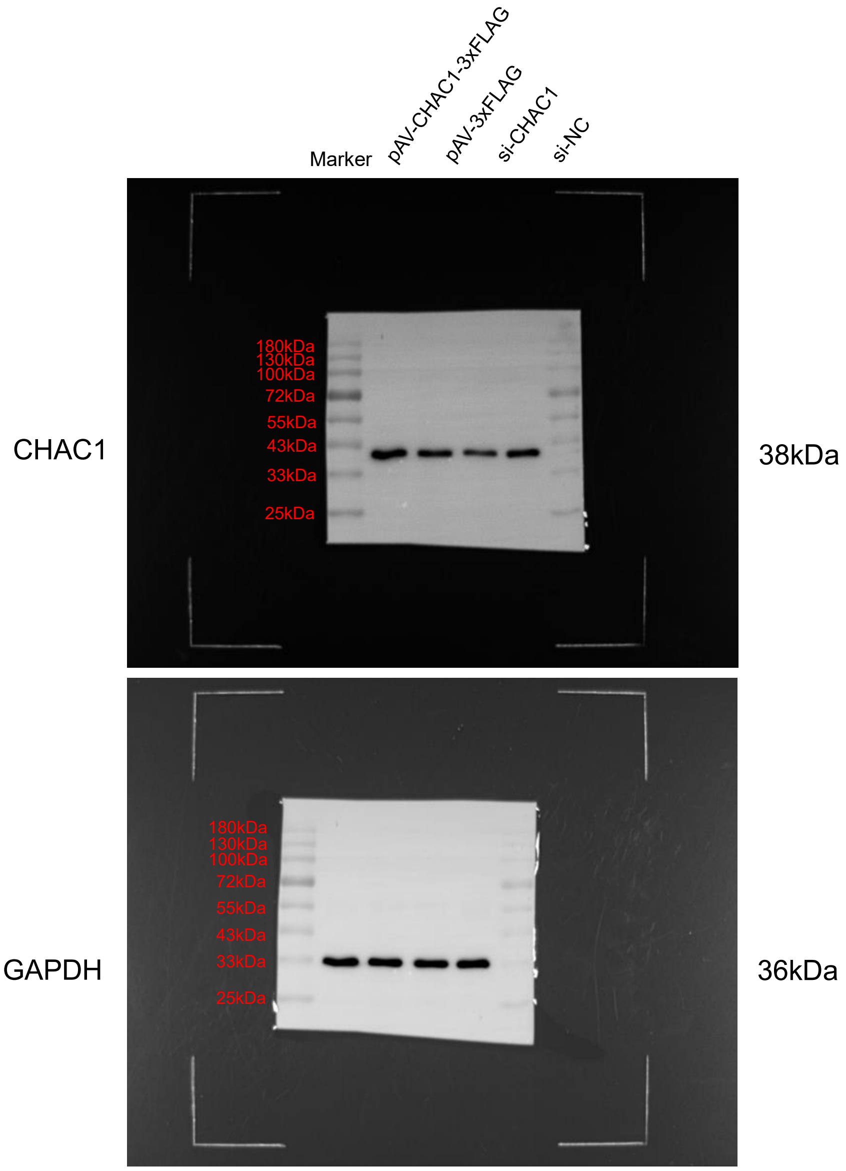

Supplementary Figure 30 Expression of CHAC1 and GAPDH in pAV-*CHAC1*-3xFlag, pAV-3xFlag, si-*CHAC1*, and si-NC. Following the editing requirements, use a load-controlled repeat western blot (a set of biological replicates), run the target protein and load-control control on the same gel and transfer them to the same membrane, and finally provide an uncropped membrane. So we firstly incubate the membrane with antibodies to one of the CHAC1 and GAPDH proteins, then wash the membrane to strip the proteins, and finally incubate the other protein on the membrane.

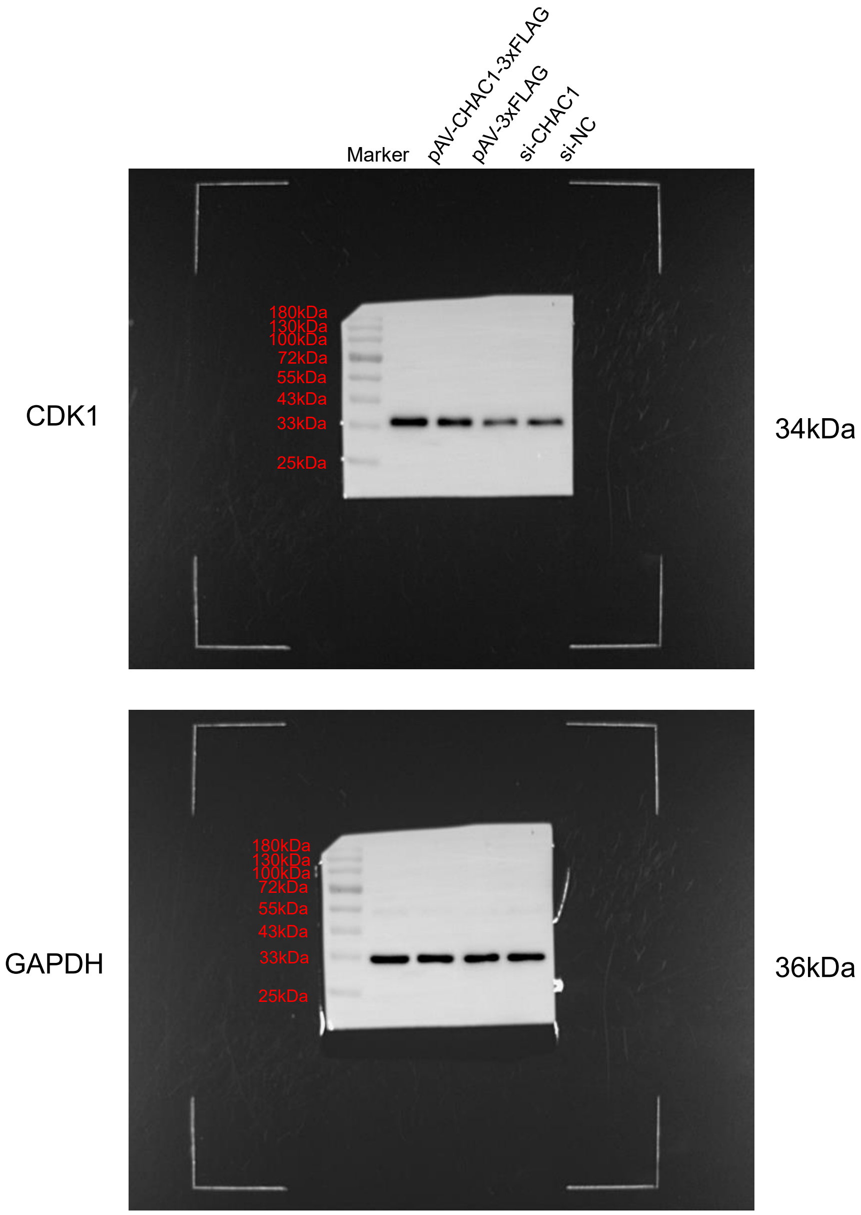

Supplementary Figure 31 The expression of CDK1 and GAPDH in pAV-*CHAC1*-3xFlag, pAV-3xFlag, si-*CHAC1*, and si-NC. Following the editing requirements, use a load-controlled repeat western blot (a set of biological replicates), run the target protein and load-control control on the same gel and transfer them to the same membrane, and finally provide an uncropped membrane. So we firstly incubate the membrane with antibodies to one of the CDK1 and GAPDH proteins, then wash the membrane to strip the proteins, and finally incubate the other protein on the membrane.

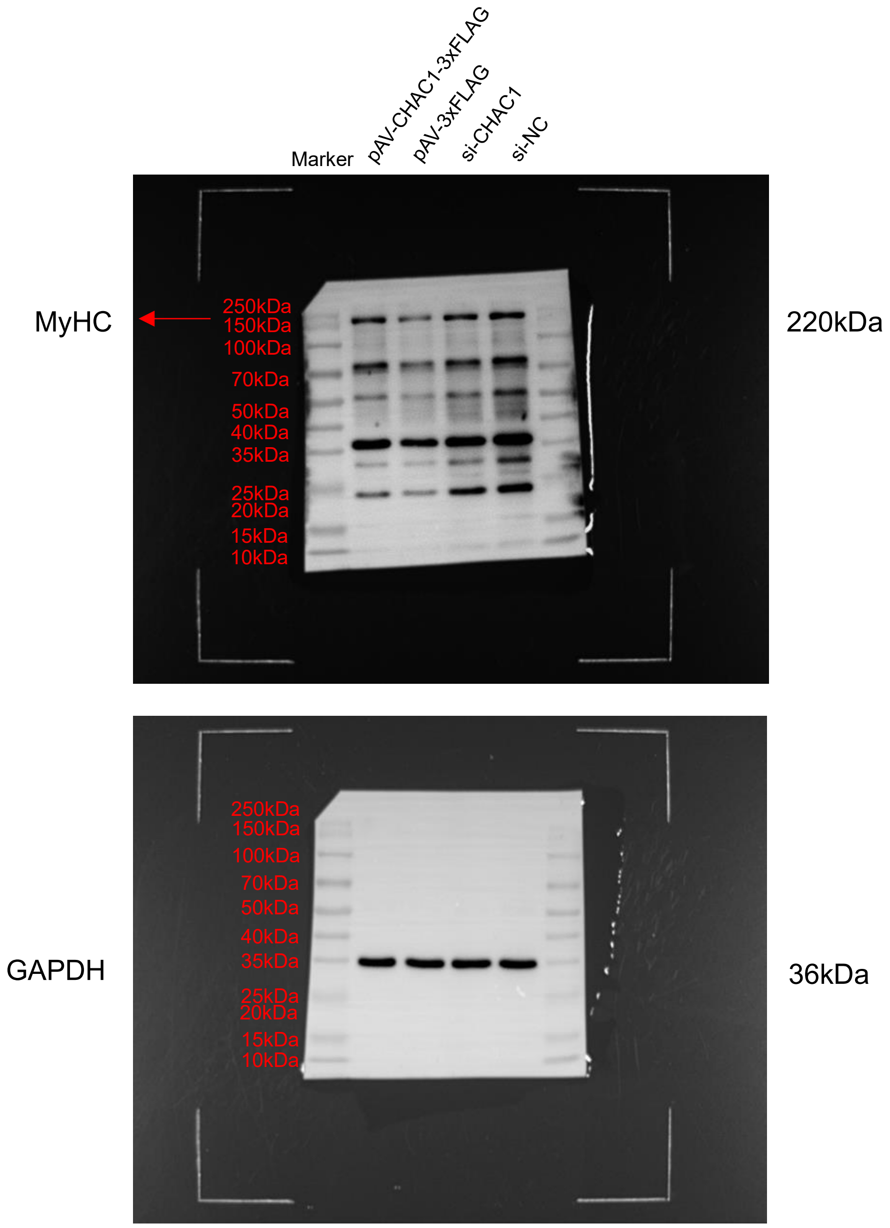

Supplementary Figure 32 The expression of MyHC and GAPDH in pAV-*CHAC1*-3xFlag, pAV-3xFlag, si-*CHAC1*, and si-NC. Following the editing requirements, use a load-controlled repeat western blot (a set of biological replicates), run the target protein and load-control control on the same gel and transfer them to the same membrane, and finally provide an uncropped membrane. So we firstly incubate the membrane with antibodies to one of the MyHC and GAPDH proteins, then wash the membrane to strip the proteins, and finally incubate the other protein on the membrane.

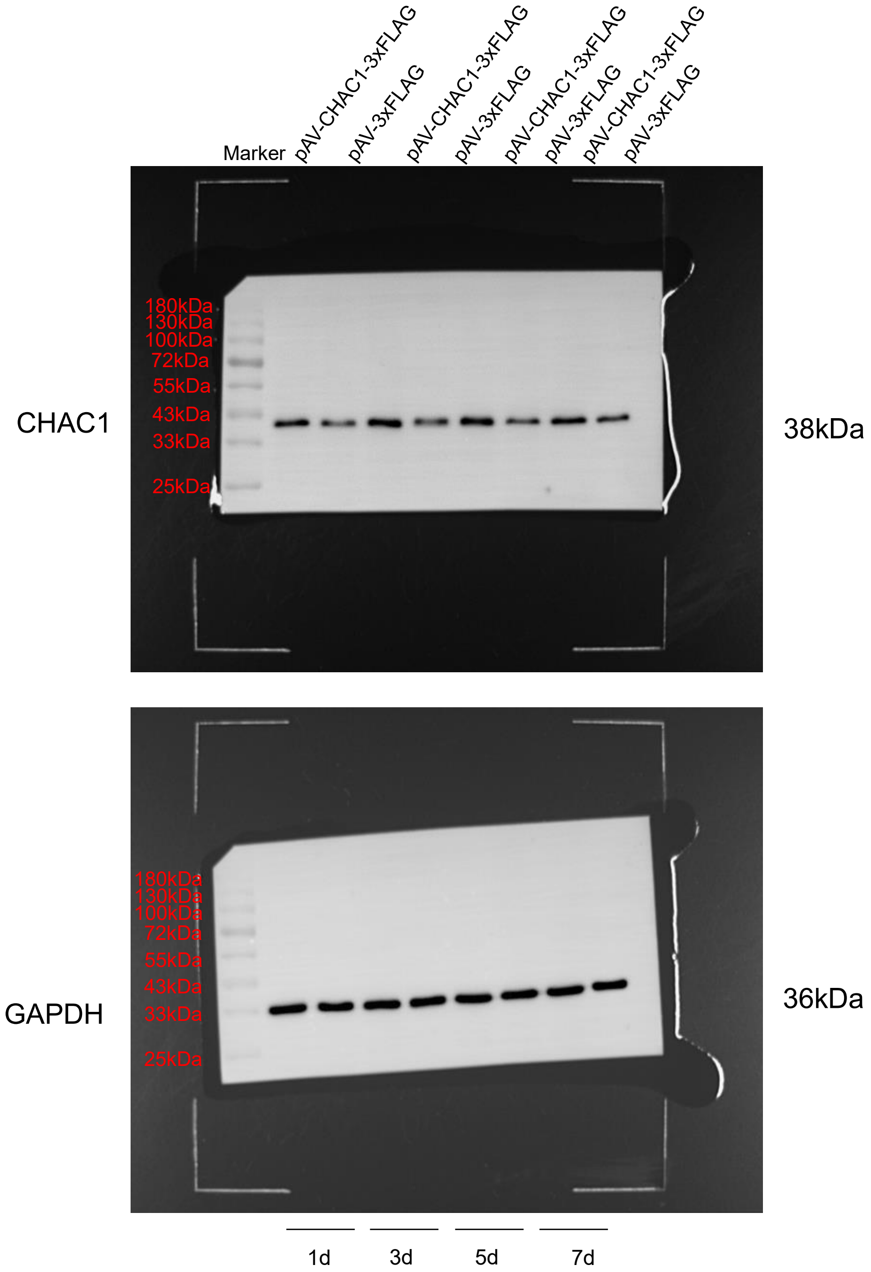

Supplementary Figure 33 The expression of CHAC1 and GAPDH after injection of pAV-*CHAC1*-3xFlag and pAV-3xFlag in the gastrocnemius muscle of AA broilers. Following the editing requirements, use a load-controlled repeat western blot (a set of biological replicates), run the target protein and load-control control on the same gel and transfer them to the same membrane, and finally provide an uncropped membrane. So we firstly incubate the membrane with antibodies to one of the CHAC1 and GAPDH proteins, then wash the membrane to strip the proteins, and finally incubate the other protein on the membrane.

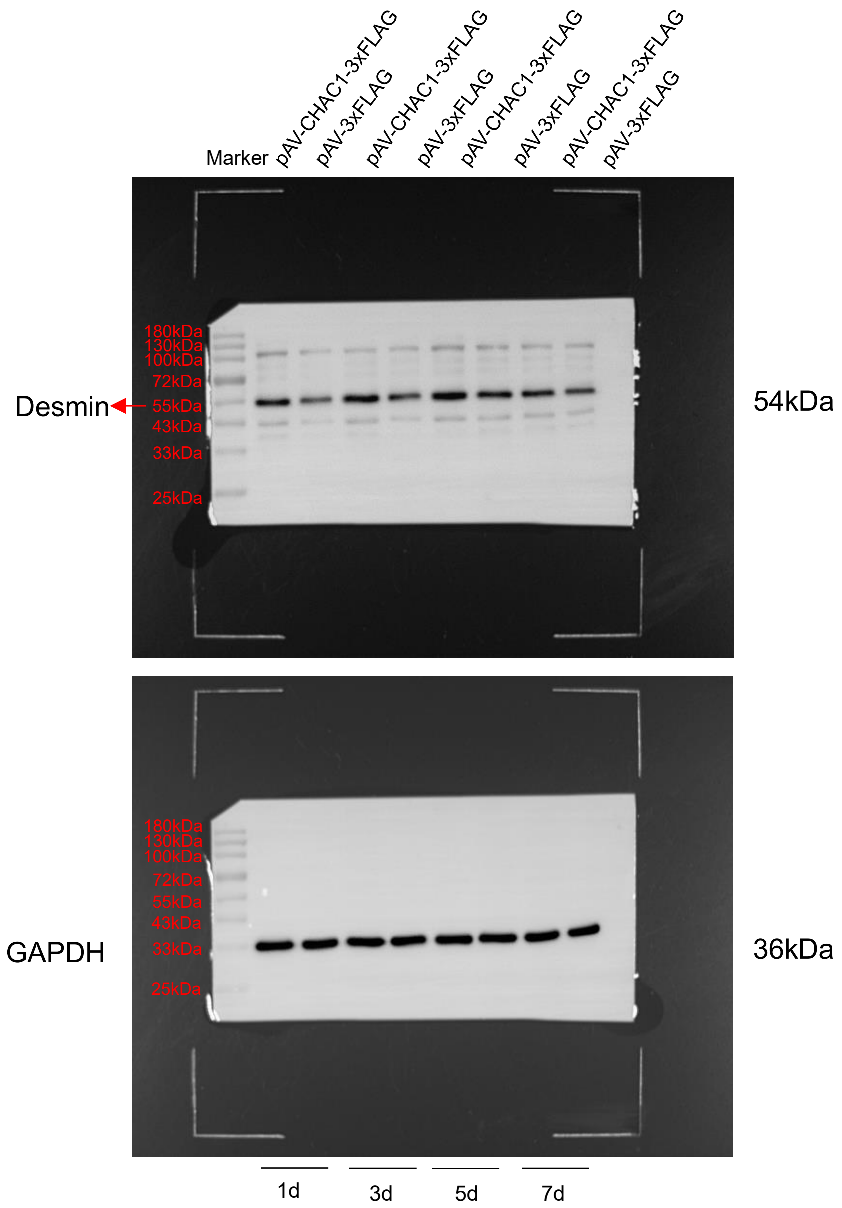

Supplementary Figure 34 The expression of Desmin and GAPDH after injection of pAV-*CHAC1*-3xFlag and pAV-3xFlag in the gastrocnemius muscle of AA broilers. Following the editing requirements, use a load-controlled repeat western blot (a set of biological replicates), run the target protein and load-control control on the same gel and transfer them to the same membrane, and finally provide an uncropped membrane. So we firstly incubate the membrane with antibodies to one of the Desmin and GAPDH proteins, then wash the membrane to strip the proteins, and finally incubate the other protein on the membrane.

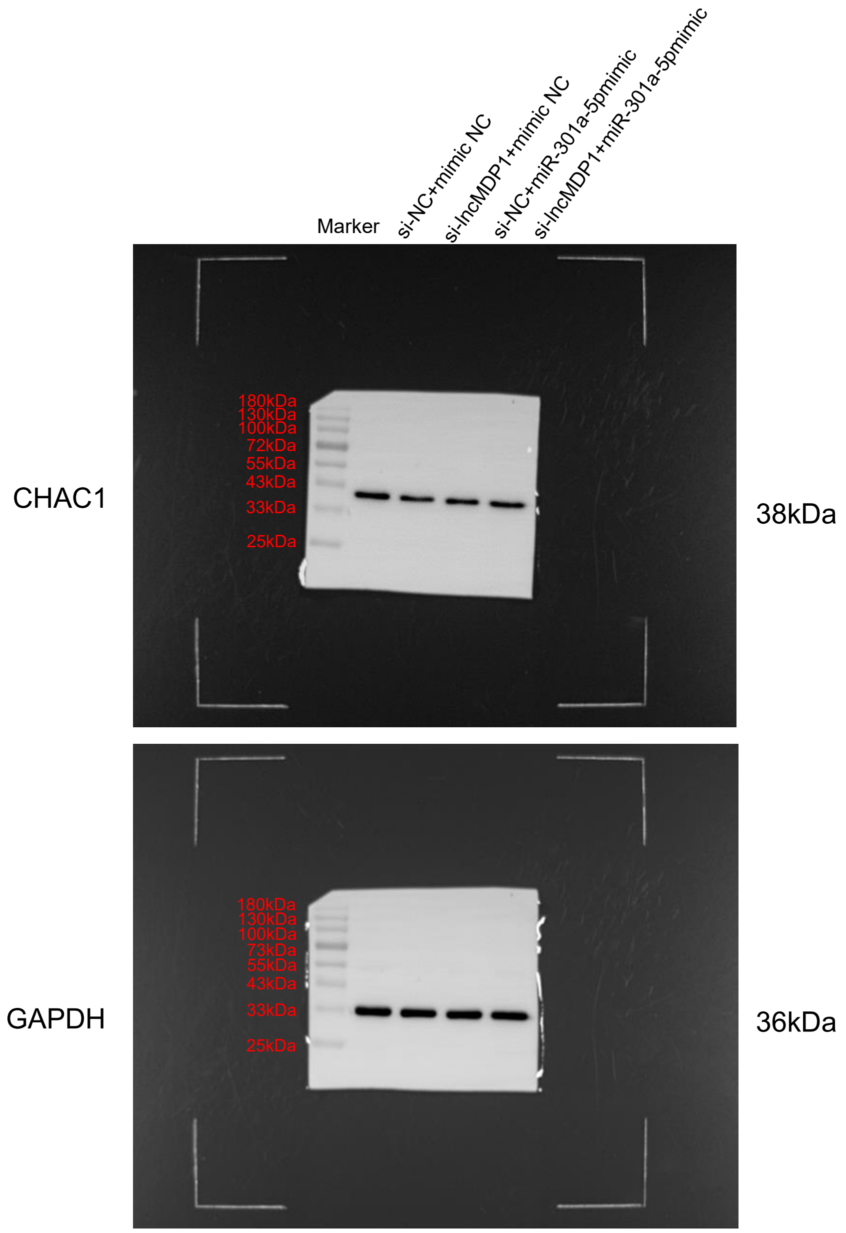

Supplementary Figure 35 The expression of CHAC1 and GAPDH in si-*lncMDP1*, si-NC, miR-301a-5p mimics, and simulated NC. Following the editing requirements, use a load-controlled repeat western blot (a set of biological replicates), run the target protein and load-control control on the same gel and transfer them to the same membrane, and finally provide an uncropped membrane. So we firstly incubate the membrane with antibodies to one of the CHAC1 and GAPDH proteins, then wash the membrane to strip the proteins, and finally incubate the other protein on the membrane.

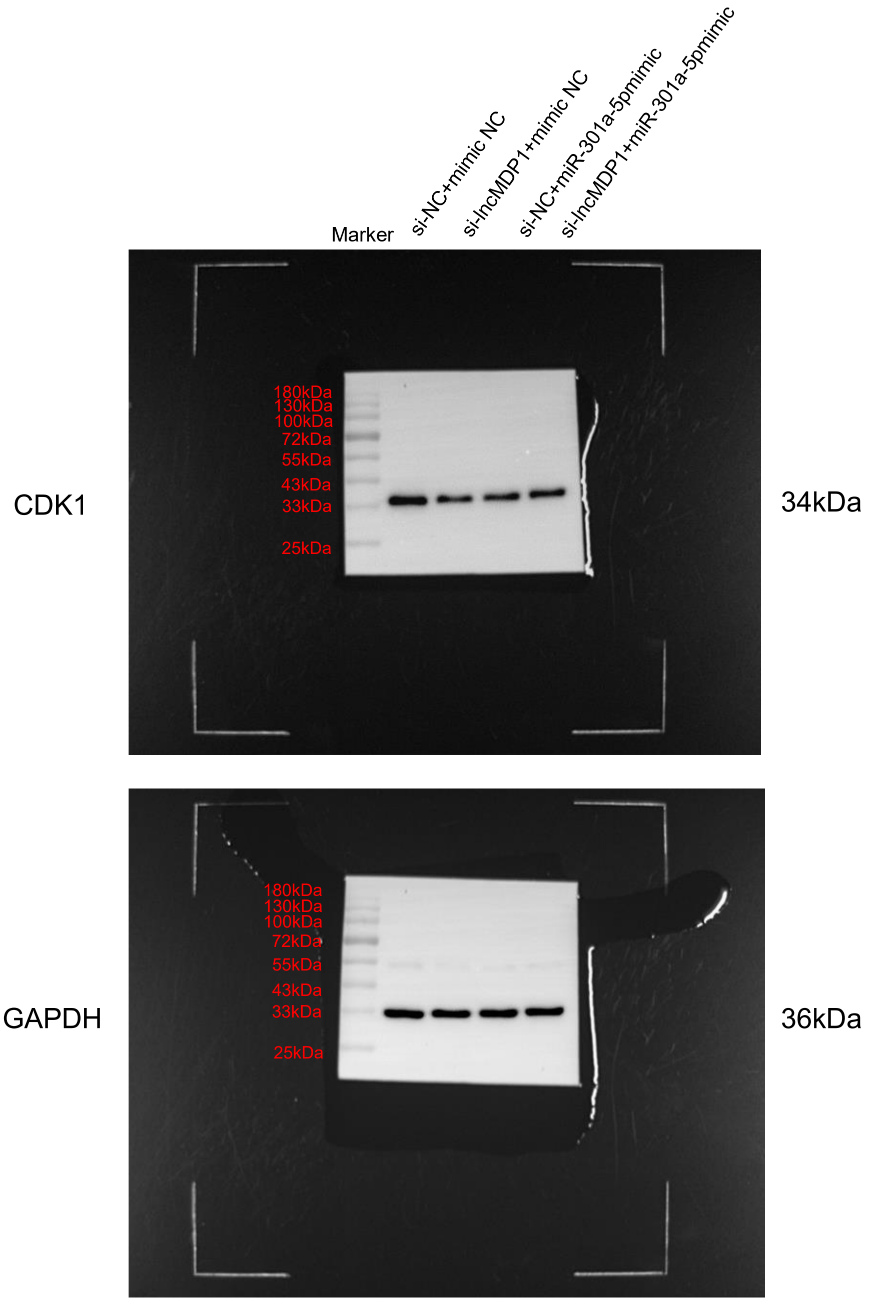

Supplementary Figure 36 The expression of CDK1 and GAPDH in si-*lncMDP1*, si-NC, miR-301a-5p mimic, and mimic NC. Following the editing requirements, use a load-controlled repeat western blot (a set of biological replicates), run the target protein and load-control control on the same gel and transfer them to the same membrane, and finally provide an uncropped membrane. So we firstly incubate the membrane with antibodies to one of the CDK1 and GAPDH proteins, then wash the membrane to strip the proteins, and finally incubate the other protein on the membrane.

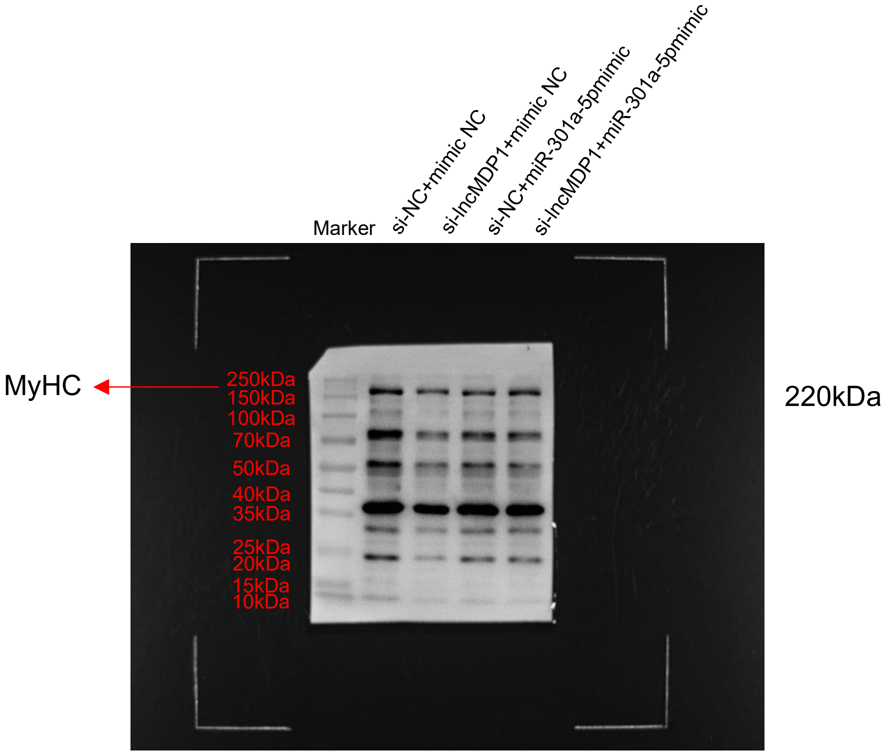

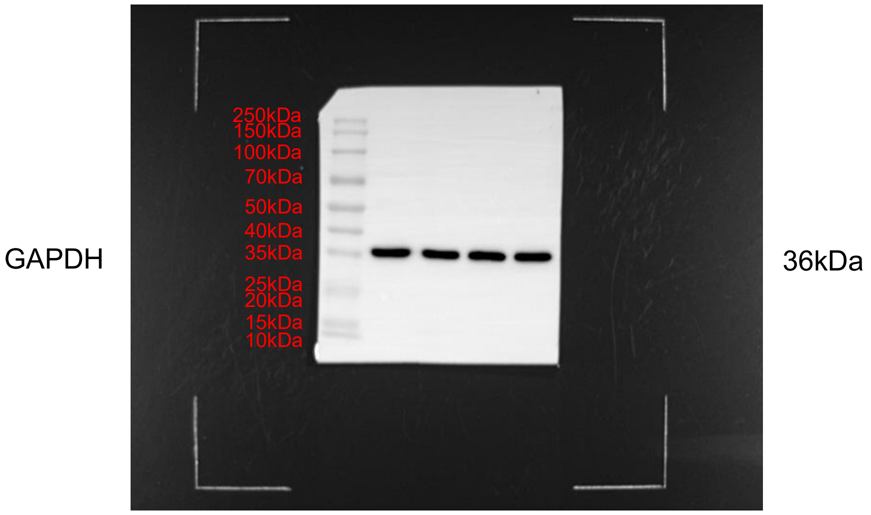

Supplementary Figure 37 The expression of MyHC and GAPDH in si-*lncMDP1*, si-NC, miR-301a-5p mimic, and mimic NC. Following the editing requirements, use a load-controlled repeat western blot (a set of biological replicates), run the target protein and load-control control on the same gel and transfer them to the same membrane, and finally provide an uncropped membrane. So we firstly incubate the membrane with antibodies to one of the MyHC and GAPDH proteins, then wash the membrane to strip the proteins, and finally incubate the other protein on the membrane.

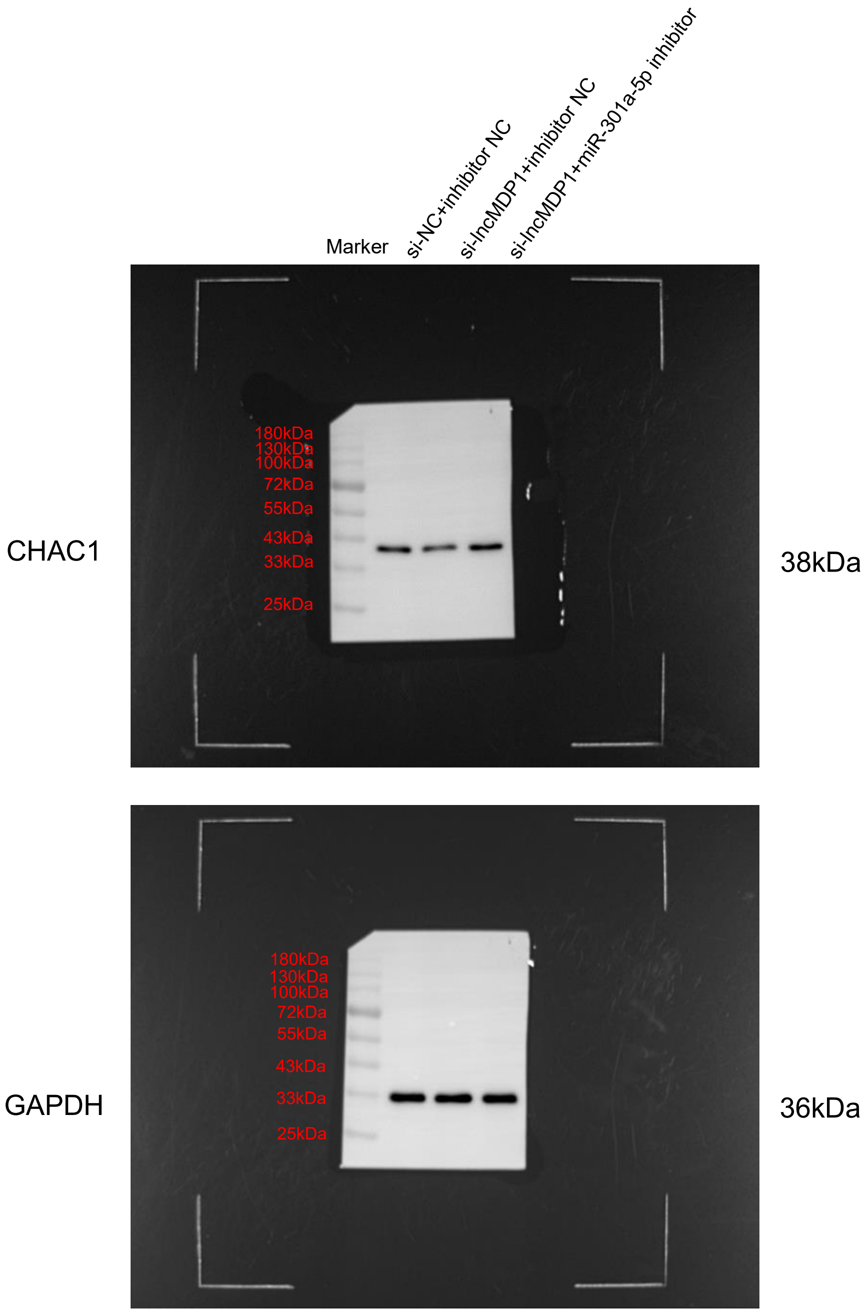

Supplementary Figure 38 The expression of CHAC1 and GAPDH in si-*lncMDP1*, si-NC, miR-301a-5p inhibitor, and inhibitor NC. Following the editing requirements, use a load-controlled repeat western blot (a set of biological replicates), run the target protein and load-control control on the same gel and transfer them to the same membrane, and finally provide an uncropped membrane. So we firstly incubate the membrane with antibodies to one of the CHAC1 and GAPDH proteins, then wash the membrane to strip the proteins, and finally incubate the other protein on the membrane.

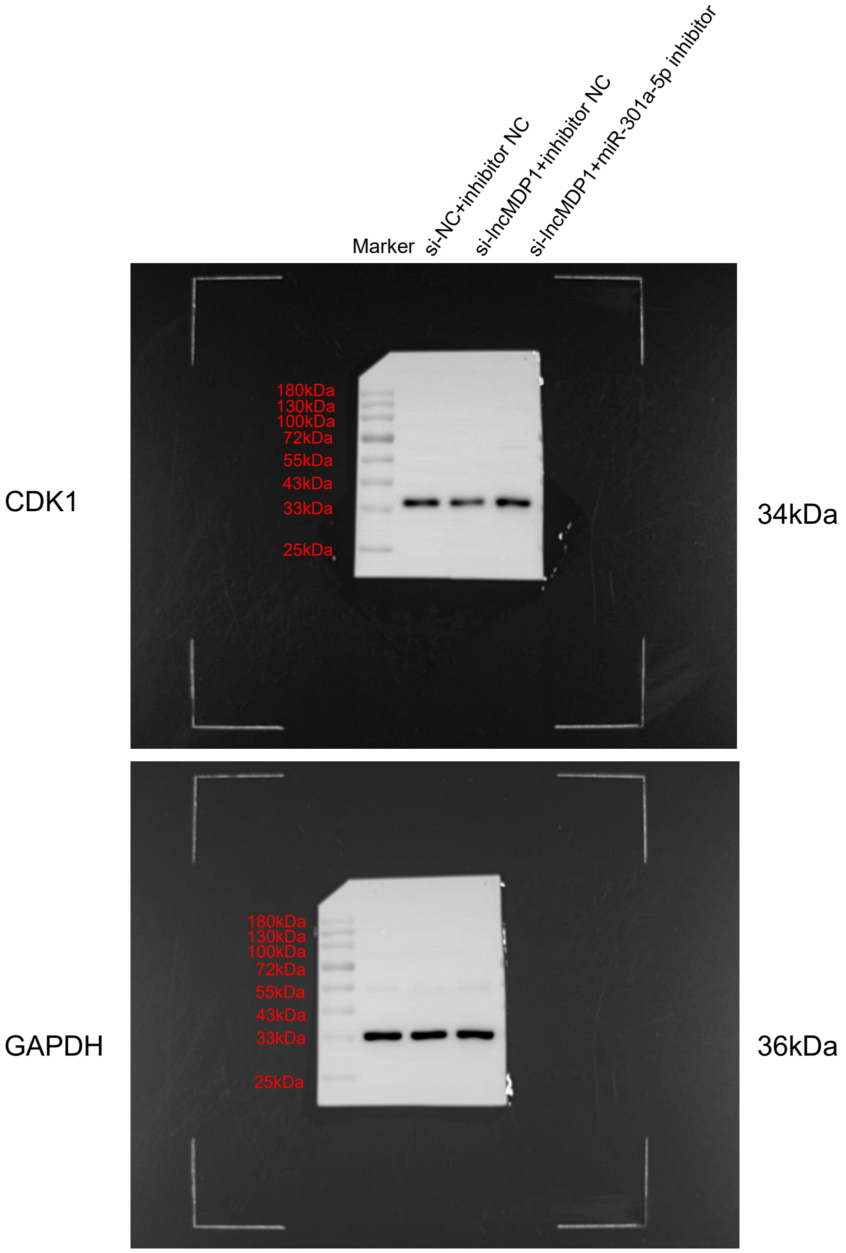

Supplementary Figure 39 The expression of CDK1 and GAPDH in si-*lncMDP1*, si-NC, miR-301a-5p inhibitor, and inhibitor NC. Following the editing requirements, use a load-controlled repeat western blot (a set of biological replicates), run the target protein and load-control control on the same gel and transfer them to the same membrane, and finally provide an uncropped membrane. So we firstly incubate the membrane with antibodies to one of the CDK1 and GAPDH proteins, then wash the membrane to strip the proteins, and finally incubate the other protein on the membrane.

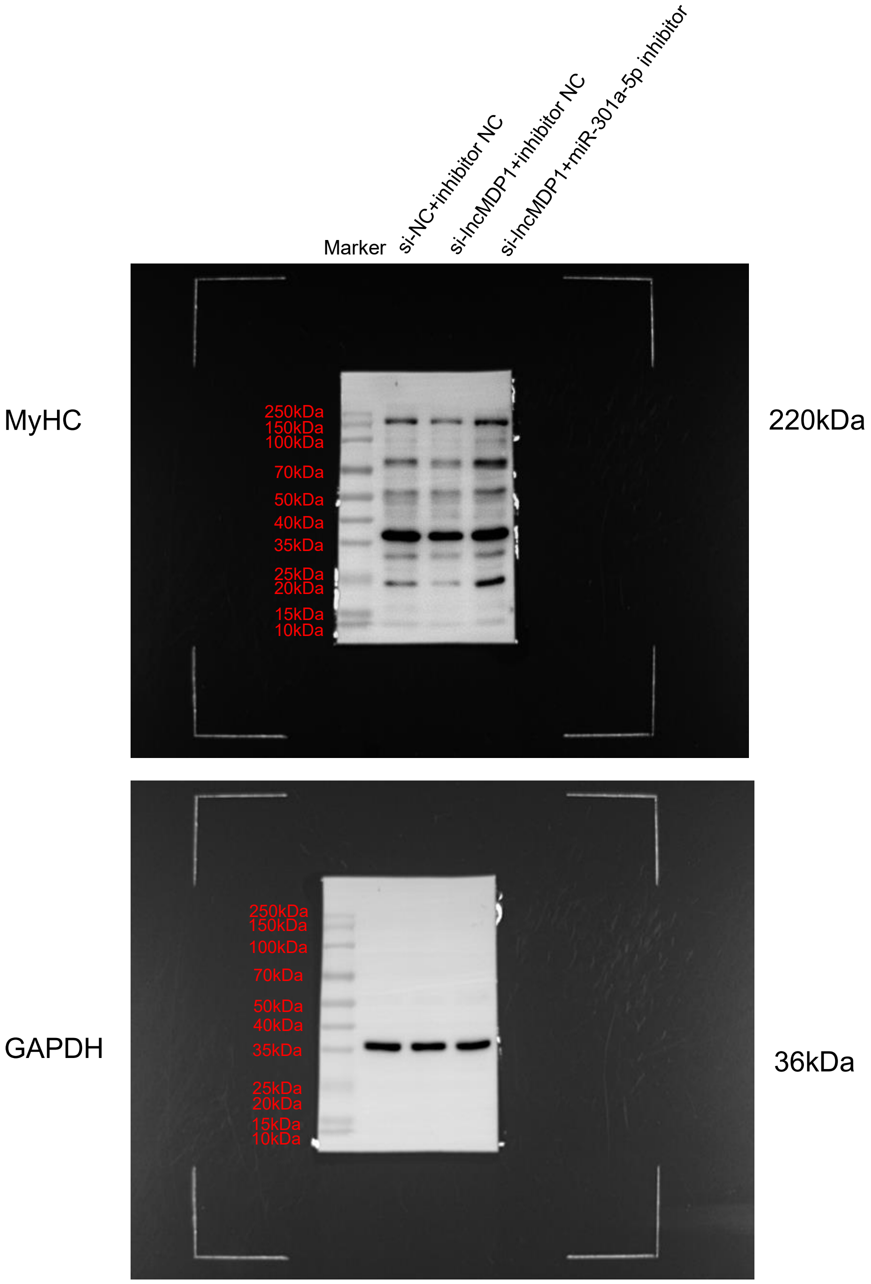

Supplementary Figure 40 The expression of MyHC and GAPDH in si-*lncMDP1*, si-NC, miR-301a-5p inhibitor, and inhibitor NC. Following the editing requirements, use a load-controlled repeat western blot (a set of biological replicates), run the target protein and load-control control on the same gel and transfer them to the same membrane, and finally provide an uncropped membrane. So we firstly incubate the membrane with antibodies to one of the MyHC and GAPDH proteins, then wash the membrane to strip the proteins, and finally incubate the other protein on the membrane.

Supplementary Table 1 DEmRANAs between 21 groups in the CPMs.

Period	Up-regulated genes	Down-regulated gene	Total
G1-vs-G2	393	655	1048
G1-vs-D1	668	1074	1742
G1-vs-D2	1289	1438	2727
G1-vs-D4	1656	1783	3439
G1-vs-D6	1653	1820	3473
G1-vs-D8	1704	1821	3525
G2-vs-D1	402	482	884
G2-vs-D2	1332	1107	2439
G2-vs-D4	1784	1555	3339
G2-vs-D6	1801	1644	3445
G2-vs-D8	1855	1670	3525
D1-vs-D2	712	486	1198
D1-vs-D4	1281	941	2222
D1-vs-D6	1356	1018	2374
D1-vs-D8	1464	1110	2574
D2-vs-D4	242	201	443
D2-vs-D6	435	474	909
D2-vs-D8	579	737	1316
D4-vs-D6	53	83	136
D4-vs-D8	227	361	588
D6-vs-D8	45	90	135

Supplementary Table 2 Primers list.

Target	Primer	Sequence（5'-3'）	AT(°C)
CHAC1 (qRT-PCR)	F	CTGGTGGACTTCATGCGCTA	60
	R	TCCTCCAGGGCTTTCTCTGA	
*LncMPD3(*qRT-PCR*)*	F	CTCCGAGACCCCAGGGATTA	60
	R	ATCATGGGGTTGCTGGTTGT	
GAPDH (qRT-PCR)	F	GAACATCATCCCAGCGTCCA	60
	R	CGGCAGGTCAGGTCAACAAC	
U6 (qRT-PCR)	RT	GTCGTATCCAGTGCAGGGTCCGAGGTATT CGCACTGGATACGACCGATACA	60
	F	GGGCCATGCTAATCTTCTCTGTATCG	
	R	GTGCAGGGTCCGAGGT	
p21 (qRT-PCR)	F	GAAGAGTTGTCCACGATAAGC	60
	R	TTCCAGTCCTCCTCAGTCC	
CDKN1A (qRT-PCR)	F	CCCGTAGACCACGAGCAGAT	60
	R	CGTCTCGGTCTCGAAGTTGA	
CDKN2B (qRT-PCR)	F	CACGGCTGCGGATGAACTAG	60
	R	TCCGACCGAAGGAGTTGACAG	
CCND1 (qRT-PCR)	F	CAGAAGTGCGAAGAGGAAGT	60
	R	CTGATGGAGTTGTCGGTGTA	
CDK1 (qRT-PCR)	F	TAATAGATGACAAAGGGGT	60
	R	GAGTGGAATACAGAGCAGA	
PCNA (qRT-PCR)	F	AGCACCAAATCAGGAAAAG	60
	R	GCACAGGAGATGACAACAG	
CCNB1 (qRT-PCR)	F	GAGGCGTGTTTGTGTAGAGC	60
	R	GATGCAGTCCGTTTTCTTGGG	
CCNB2 (qRT-PCR)	F	CCTCTTCCACTTCACTTCT	60
	R	CTTTGTACCCCACTTATCA	
CCNB3 (qRT-PCR)	F	TCACACAGGGTCTTCTTTG	60
	R	ATTTTGGTTTGTTTTTTCC	
MyoD (qRT-PCR)	F	GCTACTACACGGAATCACCAAAT	60
	R	CTGGGCTCCACTGTCACTCA	
MyoG (qRT-PCR)	F	CGGAGGCTGAAGAAGGTGAA	60
	R	CGGTCCTCTGCCTGGTCAT	
MyHC (qRT-PCR)	F	CTCCTCACGCTTTGGTAA	60
	R	TGATAGTCGTATGGGTTGGT	
Myomaker (qRT-PCR)	F	TGGGTGTCCCTGATGGC	60
	R	CCCGATGGGTCCTGAGTAG	
adult *MyHC* (qRT-PCR)	F	AGCATGAGCTGGAGGAA	60
	R	CGCTTGAGGCATCTAC	
eMyHC	F	ACTCCCAATAAACAGCG	60
	R	GATGACTTGGCATCAAAA	
Desmin	F	AACAACTTGGCTGCTTTCAGA	60
	R	GCTCACGGATTTCCTCTTCG	

Abbreviation: AT refers to the annealing temperature; F and R refer to the forward and reverse primers, respectively.
