# Supplementary material for: Whole transcriptome profiling reveals a lncMDP1 that regulates myogenesis by adsorbing miR-301a-5p targeting CHAC1
Source: Commun Biol. 2024 May 2;7:518. doi: 10.1038/s42003-024-06226-1 (PMC11066001; doi:10.1038/s42003-024-06226-1)
Supplement: Supplementary file 2 — Description of Additional Supplementary Files [file 42003_2024_6226_MOESM2_ESM.pdf]

Description of Additional Supplementary Files

File Name: Supplementary Data 1

Description: Source data behind the graphs in the figures.

File Name: Supplementary Data 2

Description: Statistical results of relevant data in the article.
