# Supplementary material for: Whole transcriptome profiling reveals a lncMDP1 that regulates myogenesis by adsorbing miR-301a-5p targeting CHAC1
Source: Commun Biol. 2024 May 2;7:518. doi: 10.1038/s42003-024-06226-1 (PMC11066001; doi:10.1038/s42003-024-06226-1)
Supplement: Supplementary file 4 — Supplementary Data 2 [file 42003_2024_6226_MOESM4_ESM.pdf]

Statistical results of relevant data in the article

Supplementary Table 3 Statistical results of RNA expression levels of *lncMDP1* in leg muscles at different time points.

Treatment	*LncMDP1*				
	Mean±S.E.M	95% CI		*t*	*p*-value
		Down	Up		
E10	1.000092±0.009572				
E12	0.8811±0.052810	-0.03004	0.26799	2.216756	0.091
E14	1.492160±0.065834	-0.767466	-0.216669	-7.396617	0.002
E16	1.387552±0.239525	-1.415734	0.640814	-1.616329	0.247016
E18	1.201521±0.109415	-0.506374	0.103517	-1.833955	0.140581
1d	1.151041±0.021766	-0.216967	-0.084931	-6.348268	0.003154
1w	0.163387±0.019334	0.774400	0.899010	34.520735	3.8371E-7
3w	0.060048±0.016817	0.884908	0.995180	43.827203	1.1673E-7
5w	0.040216±0.003168	0.937145	0.982606	108.551631	1.2581E-9

Supplementary Table 4 The statistical results of the relative expression levels of *CDK1*, *PCNA*, *CCND1*, *CCNB2*, *P21* after si-*lncMDP1* transfection of proliferating myoblasts for 48 h.

Index	Treatment	Mean±S.E.M	95% CI		*t*	*p*-value
			Down	Up		
CDK1	si-NC	1.009798±0.101737				
	si-*lncMDP1*	0.718604±0.082327	-0.072171	0.654560	2.224990	0.090111
PCNA	si-NC	1.000706±0.026445				
	si-*lncMDP1*	0.833462±0.011995	0.086621	0.247867	5.759427	0.004508
CCND1	si-NC	1.000243±0.015516				
	si-*lncMDP1*	0.742042±0.010976	0.205432	0.310970	13.585291	0.000170
CCNB2	si-NC	1.004870±0.069489				
	si-*lncMDP1*	0.818578±0.027958	-0.021669	0.394253	2.487143	0.067691
P21	si-NC	1.003410±0.059203				
	si-*lncMDP1*	1.612375±0.084878	-0.896286	-0.321642	-5.884535	0.004169

Supplementary Table 5 Statistical results of CDK1 protein expression level after *lncMDP1* interference

Treatment	CDK1				
	Mean±S.E.M	95% CI		*t*	*p*-value
		Down	Up		
si-NC	1.000000±0.032204				
si-*lncMDP1*	0.657061±0.016865	0.242007	0.443870	9.433607	0.000704

Supplementary Table 6 Statistical analysis of cell cycle after si-*lncMDP1* transfection into myoblasts

Index	Treatment	Mean±S.E.M	95% CI		t	p-value
			Down	Up		
G0/G1	si-NC	84.620000±0.265141				
	si-*lncMDP1*	83.660000±0.316596	-0.186552	2.106552	2.324698	0.080720
S	si-NC	11.913333±0.056667				
	si-*lncMDP1*	10.960000±0.299555	0.106884	1.799782	3.127037	0.035286
G2	si-NC	3.930000±0.240000				
	si-*lncMDP1*	5.383333±0.049103	-2.433166	-0.473501	-5.932659	0.022499

Supplementary Table 7 CCK-8 was used to detect the statistical results of the effect of interfering *lncMDP1* on the proliferation of myoblasts.

Index	Treatment	Mean±S.E.M	95% CI		*t*	*p*-value
			Down	Up		
12 h	si-NC	0.437833±0.002915				
	si-*lncMDP1*	0.417600±0.007534	-0.000301	0.040768	2.504738	0.052378
24 h	si-NC	0.370667±0.006702				
	si-*lncMDP1*	0.396800±0.012886	-0.057330	0.005063	-1.894998	0.090610
36 h	si-NC	0.492000±0.011349				
	si-*lncMDP1*	0.428400±0.013045	0.023728	0.103472	3.678329	0.006234
48 h	si-NC	0.543200±0.011901				
	si-*lncMDP1*	0.537600±0.005591	-0.024722	0.035922	0.425883	0.681419

Supplementary Table 8 Statistical analysis of EdU staining of myoblast proliferation after si-*lncMDP1* and si-NC transfection.

Index	Treatment	Mean±S.E.M	95% CI		*t*	*p*-value
			Down	Up		
EdU	si-NC	0.912605±0.089777				
	si-*lncMDP1*	0.548512±0.009644	0.155875	0.572311	4.032313	0.003776

Supplementary Table 9 Statistical analysis of RNA expression levels of myoblast differentiation marker genes *MyoD*, *MyoG*, *MyHC* and *Myomarker* by interfering with *lncMDP1*.

Index	Treatment	Mean±S.E.M	95% CI		*t*	*p*-value
			Down	Up		
MyHC	si-NC	1.001588±0.040381				
	si-*lncMDP1*	0.920529±0.106571	-0.235357	0.397475	0.711265	0.081059
MyoD	si-NC	1.000155±0.010151				
	si-*lncMDP1*	0.722228±0.033111	0.193186	0.362669	8.025160	0.000200
MyoG	si-NC	1.001109±0.032977				
	si-*lncMDP1*	0.790749±0.017052	0.122496	0.298224	6.154379	0.001647
Myomaker	si-NC	1.001264±0.035992				
	si-*lncMDP1*	0.681566±0.046854	0.155660	0.483738	5.411071	0.005650

Supplementary Table 10 Statistical analysis of MyHC immunofluorescence after interference with *lncMDP1* in myoblasts.

Index	Treatment	Mean±S.E.M	95% CI		*t*	*p*-value
			Down	Up		
Immunoflu-orescence	si-NC	1.000000±0.013680				
	si-*lncMDP1*	0.516066±0.035536	0.000221	0.589656	12.708913	0.000221

Supplementary Table 11 Statistical results of MyHC protein expression level after *lncMDP1* interference

Treatment	MyHC				
	Mean±S.E.M	95% CI		*t*	*p*-value
		Down	Up		
si-NC	3.642188±0.130895				
si-*lncMDP1*	2.526963±0.097109	0.662712	1.567739	6.842583	0.002387

Supplementary Table 12 Statistical results of RNA expression levels of miR-301a-5p in leg muscles at different time points.

Treatment	miR-301a-5p				
	Mean±S.E.M	95% CI		*t*	*p*-value
		Down	Up		
E10	1.000174±0.013154				
E12	0.702521±0.082966	0.064425	0.530881	3.543385	0.023940
E14	1.414344±0.068417	-0.695025	-0.133314	-5.944742	0.022903
E16	3.742565±0.407753	-4.039195	-1.445587	-6.722116	0.006676
E18	5.264081±0.146787	-4.673086	-3.854727	-28.932276	0.000008
1d	4.705536±0.325816	-4.741196	-2.669528	-11.363284	0.001440
1w	9.873181±0.252390	-9.574704	-8.171309	-35.108296	0.000004
3w	8.082730±0.527710	0.884908	-9.351116	-4.813995	0.005482

Supplementary Table 13 The statistical results of the binding of *lncMDP1* to miR-301a-5p in DF-1 cells were detected by dual luciferase reporter system.

Treatment	Mean±S.E.M	95% CI		t	p-value
		Down	Up		
LncMDP1 WT+ mimic NC	1.000000±0.027311				
LncMDP1 WT+miR-301a-5p mimic	0.734813±0.003049	0.149662	0.380712	9.650072	0.009747
LncMDP1 MT+ mimic NC	1.021045±0.027291				
LncMDP1 MT+miR-301a-5p mimic	0.985545±0.007209	-0.072806	0.143805	1.257652	0.322058

Supplementary Table 14 The statistical results of *lncMDP1* expression were detected after overexpression and interference of miR-301a-5p.

Index	Treatment	Mean±S.E.M	95% CI		*t*	*p*-value
			Down	Up		
LncMDP1	mimic NC	1.001801±0.043026				
	miR-301a-5p mimic	0.706626±0.060731	0.088530	0.501820	3.965914	0.016595
	inhibitor NC	1.001493±0.039013				
	miR-301a-5p inhibitor	1.134004±0.018136	-0.251961	-0.013061	-3.080034	0.036930

Supplementary Table 15 The statistical results of the relative expression levels of *CDK1, PCNA, CCND1, CCNB2, CCNB3, CDKN1A, CDKN2B* after transfection with miR-181a-5p mimics.

Index	Treatment	Mean±S.E.M	95% CI		*t*	*p*-value
			Down	Up		
CDK1	mimic NC	1.010190±0.104445				
	miR-301a-5p mimic	0.315686±0.021426	0.268526	1.120483	6.513845	0.018463
PCNA	mimic NC	1.000690±0.009010				
	miR-301a-5p mimic	0.761557±0.011995	0.162482	0.315784	8.661831	0.000977
CCND1	mimic NC	1.006098±0.017295				
	miR-301a-5p mimic	0.813379±0.028831	0.099374	0.286065	5.732178	0.004587
CCNB2	mimic NC	1.006873±0.084320				
	miR-301a-5p mimic	0.550787±0.021202	0.214688	0.697484	5.245683	0.006316
CCNB3	mimic NC	1.005073±0.072499				
	miR-301a-5p mimic	0.613152±0.045414	0.183614	0.600228	4.836454	0.004730
CDKN1A	mimic NC	1.001407±0.026819				
	miR-301a-5p mimic	1.138417±0.011107	-0.226730	-0.047290	-3.736642	0.009661
CDKN2B	mimic NC	1.001294±0.035601				
	miR-301a-5p mimic	1.516944±0.219515	-1.428120	0.396821	-2.318747	0.139938

Supplementary Table 16 The statistical results of the relative expression levels of *CDK1, PCNA, CCND1, CCNB2, CCNB3, P21, CDKN2B* after transfection with miR-181a-5p inhibitor.

Index	Treatment	Mean±S.E.M	95% CI		*t*	*p*-value
			Down	Up		
CDK1	inhibitor NC	1.000439±0.021101				
	miR-301a-5p inhibitor	1.383185±0.040562	-0.509691	-0.255801	-8.371114	0.001114
PCNA	inhibitor NC	1.000069±0.008306				
	miR-301a-5p inhibitor	1.153444±0.004796	-0.180005	-0.126745	-15.990987	0.000089
CCND1	inhibitor NC	1.003908±0.063669				
	miR-301a-5p inhibitor	1.287718±0.051586	-0.511325	-0.056295	-3.463433	0.025737
CCNB2	inhibitor NC	1.010311±0.105192				
	miR-301a-5p inhibitor	1.354387±0.054628	-0.673171	-0.014981	-2.902836	0.043993
CCNB3	inhibitor NC	1.000526±0.023113				
	miR-301a-5p inhibitor	1.235362±0.045414	-0.394631	-0.075040	-4.080262	0.015095
P21	inhibitor NC	1.002655±0.050857				
	miR-301a-5p inhibitor	0.629129±0.027307	0.213257	0.533794	6.470856	0.002939
CDKN1A	inhibitor NC	1.001538±0.039040				
	miR-301a-5p inhibitor	0.705554±0.038379	0.143986	0.447982	5.406528	0.005668

Supplementary Table 17 Statistical results of CDK1 protein expression level after miR-301a-5p mimic and inhibitor.

Treatment	CDK1				
	Mean±S.E.M	95% CI		*t*	*p*-value
		Down	Up		
mimic NC	1.000000±0.091676				
miR-301a-5p mimic	0.524391±0.141971	0.006397	0.944822	2.814296	0.048106
inhibitor NC	1.000000±0.104406				
miR-301a-5p inhibitor	1.272773±0.191273	-0.877797	0.332251	-1.251751	0.278864

Supplementary Table 18 CCK-8 was used to detect the statistical results of the effect of miR-301a-5p mimic on the proliferation of myoblasts.

Index	Treatment	Mean±S.E.M	95% CI		*t*	*p*-value
			Down	Up		
12 h	mimic NC	0.719571±0.021764				
	miR-301a-5p mimic	0.560333±0.023998	0.088056	0.230420	4.923726	0.000454
24 h	mimic NC	1.012667±0.021316				
	miR-301a-5p mimic	0.791500±0.079011	0.028718	0.304329	2.805234	0.023885
36 h	mimic NC	1.111400±0.057434				
	miR-301a-5p mimic	0.934600±0.014109	0.040419	0.313181	2.989439	0.017349
48 h	mimic NC	1.344000±0.018955				
	miR-301a-5p mimic	1.199500±0.027866	0.072165	0.216835	4.396768	0.001069

Supplementary Table 19 CCK-8 was used to detect the statistical results of the effect of miR-301a-5p inhibitor on the proliferation of myoblasts.

Index	Treatment	Mean±S.E.M	95% CI		*t*	*p*-value
			Down	Up		
12 h	inhibitor NC	0.619750±0.025519				
	miR-301a-5p inhibitor	0.646000±0.010103	-0.081136	0.028636	-1.102885	0.302146
24 h	inhibitor NC	0.730750±0.028990				
	miR-301a-5p inhibitor	0.875667±0.029146	-0.244172	-0.045662	-3.366871	0.009831
36 h	inhibitor NC	0.892500±0.032615				
	miR-301a-5p inhibitor	1.015250±0.070390	-0.312579	0.067079	-1.582261	0.164677
48 h	inhibitor NC	0.765000±0.089634				
	miR-301a-5p inhibitor	0.932600±0.009605	-0.547225	0.212025	-1.859173	0.201234

Supplementary Table 20 Statistical analysis of EdU staining of myoblast proliferation after miR-301a-5p mimic and inhibitor transfection.

Index	Treatment	Mean±S.E.M	95% CI		*t*	*p*-value
			Down	Up		
EdU	mimic NC	1.000000±0.017600				
	miR-301a-5p mimic	0.823405±0.020022	0.107893	0.245297	6.607534	0.001194
	inhibitor NC	1.000000±0.059430				
	miR-301a-5p inhibitor	1.128640±0.009697	-0.375580	0.118299	-2.136331	0.159685

Supplementary Table 21 Statistical analysis of cell cycle after miR-310a-5p mimic transfection into myoblasts

Index	Treatment	Mean±S.E.M	95% CI		*t*	*p*-value
			Down	Up		
G0/G1	mimic NC	82.576667±0.204803				
	miR-301a-5p mimic	85.593333±0.486632	-4.482555	-1.550778	-5.713675	0.004641
S	mimic NC	13.696667±0.155063				
	miR-301a-5p mimic	10.960000±0.036056	2.294658	3.178675	17.190184	0.000067
G2	mimic NC	3.726667±0.258607				
	miR-301a-5p mimic	3.446667±0.519658	-1.331588	1.891588	0.482384	0.654742

Supplementary Table 22 Statistical analysis of cell cycle after miR-310a-5p inhibitor transfection into myoblasts.

Index	Treatment	Mean±S.E.M	95% CI		*t*	*p*-value
			Down	Up		
G0/G1	inhibitor NC	56.553333±0.512651				
	miR-301a-5p inhibitor	56.826667±1.022258	-3.448477	2.901811	-0.239011	0.822844
S	inhibitor NC	26.546667±0.290421				
	miR-301a-5p inhibitor	29.603333±0.210739	-4.052925	-2.060408	-8.518539	0.001042
G2	inhibitor NC	3.726667±0.258607				
	miR-301a-5p inhibitor	3.446667±0.519658	-1.331588	1.891588	0.482384	0.654742

Supplementary Table 23 Statistical analysis of MyHC immunofluorescence after transfection of miR-301a-5p mimic and inhibitor in myoblasts.

Index	Treatment	Mean±S.E.M	95% CI		*t*	*p*-value
			Down	Up		
Immunofluorescence	mimic NC	12.315573±0.398387	3.033201	6.319026	11.548720	0.005860
	miR-301a-5p mimic	7.639460±0.072352				
	inhibitor NC	4.717860±0.687528	-7.490764	-2.248796	-5.158626	0.006704
	miR-301a-5p inhibitor	9.587640±0.646881				

Supplementary Table 24 Statistical analysis of RNA expression levels of myoblast differentiation marker genes *MyoD, MyoG* and *MyHC* transfected with miR-301a-5p mimic.

Index	Treatment	Mean±S.E.M	95% CI		*t*	*p*-value
			Down	Up		
MyHC	mimic NC	1.004530±0.055653				
	miR-301a-5p mimic	0.739678±0.036955	0.101386	0.428317	3.964557	0.007413
MyoD	mimic NC	1.000214±0.011923				
	miR-301a-5p mimic	1.040623±0.032526	-0.119203	0.038385	-1.318303	0.244561
MyoG	mimic NC	1.002133±0.046755				
	miR-301a-5p mimic	0.685555±0.032052	0.159193	0.473964	5.584780	0.005042

Supplementary Table 25 Statistical analysis of RNA expression levels of myoblast differentiation marker genes *MyoD, MyoG* and *MyHC* transfected with miR-301a-5p inbibitor.

Index	Treatment	Mean±S.E.M	95% CI		*t*	*p*-value
			Down	Up		
MyHC	mimic NC	1.000557±0.019400				
	miR-301a-5p mimic	1.300120±0.064633	-0.497021	-0.102104	-4.439198	0.014926
MyoD	mimic NC	1.000965±0.024976				
	miR-301a-5p mimic	1.090729±0.009324	-0.168333	-0.011195	-2.936841	0.032375
MyoG	mimic NC	1.000249±0.015866				
	miR-301a-5p mimic	1.150698±0.051669	-0.300517	-0.000381	-2.783490	0.049641

Supplementary Table 26 Statistical results of MyHC protein expression level after miR-301a-5p mimic and inhibitor.

Treatment	MyHC				
	Mean±S.E.M	95% CI		*t*	*p*-value
		Down	Up		
mimic NC	1.000000±0.152809				
miR-301a-5p mimic	0.483231±0.093633	0.019190	1.014348	2.883521	0.044854
inhibitor NC	1.000000±0.097345				
miR-301a-5p inhibitor	2.066079±0.178650	-1.630948	-0.501210	-5.239992	0.006340

Supplementary Table 27 Statistical results of RNA expression levels of *CHAC1* in leg muscles at different time points.

Treatment	*CHAC1*				
	Mean±S.E.M	95% CI		*t*	*p*-value
		Down	Up		
E10	1.010976±0.106338				
E12	0.614669±0.065793	0.092416	0.700197	3.352323	0.020277
E14	0.407486±0.013072	0.306025	0.900954	5.632790	0.004888
E16	0.050607±0.015518	0.661999	1.258738	8.936606	0.000867
E18	0.301549±0.081464	0.337505	1.081348	5.295962	0.006104
1d	1.465742±0.275742	-1.275306	0.365774	-1.538782	0.198688
1w	0.351471±0.054234	0.328080	0.990928	5.524879	0.005242
3w	0.545338±0.076740	0.101543	0.829732	3.550776	0.023782
5w	0.475656±0.225310	-0.156413	1.227053	2.148640	0.098131

Supplementary Table 28 The statistical results of the binding of *CHAC1* to miR-301a-5p in DF-1 cells were detected by dual luciferase reporter system.

Treatment	Mean±S.E.M	95% CI		t	p-value
		Down	Up		
CHAC1-3'UTR WT + mimic NC	0.991872±0.009540				
CHAC1-3'UTR WT+ miR-301a-5p mimic	0.817715±0.025130	-0.235501	-0.112814	-7.298002	0.000756
CHAC1-3'UTR MT + mimic NC	0.977884±0.021326				
CHAC1-3'UTR MT +miR-301a-5p mimic	1.000000±0.031114	-0.126846	0.082615	-0.586298	0.589165

Supplementary Table 29 The statistical results of *CHAC1* expression were detected after overexpression and interference of miR-301a-5p.

Index	Treatment	Mean±S.E.M	95% CI		*t*	*p*-value
			Down	Up		
CHAC1	mimic NC	1.008449±0.093606				
	miR-301a-5p mimic	0.598631±0.044753	0.166801	0.652836	4.334958	0.007464
	inhibitor NC	1.004602±0.068438				
	miR-301a-5p inhibitor	1.385886±0.033758	-0.560748	-0.201820	-5.461370	0.002800

Supplementary Table 30 The statistical results of the relative expression levels of *CDK1*, *PCNA*, *CCNB1*, *CCNB2*, *CCND1*, *P21*, *CDKN1A* after transfection with pcDNA3.1-*CHAC1*-3xFlag and pcDNA3.1-3xFlag.

Index	Treatment	Mean±S.E.M	95% CI		*t*	*p*-value
			Down	Up		
CDK1	pcDNA3.1-3xFlag	1.021574±0.152579				
	pcDNA3.1-*CHAC1*-3xFlag	3.396621±0.463148	-3.728934	-1.021161	-4.870563	0.008216
PCNA	pcDNA3.1-3xFlag	1.007327±0.084584				
	pcDNA3.1-*CHAC1*-3xFlag	1.570163±0.160442	-1.066408	-0.059264	-3.103196	0.036109
CCNB1	pcDNA3.1-3xFlag	1.001212±0.028850				
	pcDNA3.1-*CHAC1*-3xFlag	2.255199±0.225417	-1.964411	-0.543561	-5.517958	0.010763
CCNB2	pcDNA3.1-3xFlag	1.000579±0.019503				
	pcDNA3.1-*CHAC1*-3xFlag	1.371699±0.250343	-0.912792	0.170553	-1.761201	0.138509
CCND1	pcDNA3.1-3xFlag	1.001219±0.034634				
	pcDNA3.1-*CHAC1*-3xFlag	1.230241±0.069495	-0.444604	-0.013439	-2.949529	0.041989
P21	pcDNA3.1-3xFlag	1.000295±0.017260				
	pcDNA3.1-*CHAC1*-3xFlag	0.920311±.013283	0.019516	0.140452	3.672523	0.021344
CDKN1A	pcDNA3.1-3xFlag	1.003872±0.061415				
	pcDNA3.1-*CHAC1*-3xFlag	0.515156±0.080460	0.207684	0.769748	4.828253	0.008472

Supplementary Table 31 The statistical results of the relative expression levels of *CDK1*, *PCNA*, *CCNB1*, *CCNB2*, *CCND1*, *P21*, *CDKN1A* after transfection with si-*CHAC1* and si-NC.

Index	Treatment	Mean±S.E.M	95% CI		*t*	*p*-value
			Down	Up		
CDK1	si-NC	1.008153±0.091238				
	si-*CHAC1*	0.816149±0.072182	-0.131003	0.515011	1.650395	0.174207
PCNA	si-NC	1.000260±0.016186				
	si-*CHAC1*	0.786632±0.061480	-0.030464	0.457721	3.360270	0.065371
CCND1	si-NC	1.003698±0.060315				
	si-*CHAC1*	0.782340±0.042936	0.015799	0.426916	2.989836	0.040345
CCNB2	si-NC	1.000467±0.021464				
	si-*CHAC1*	0.776805±0.001742	0.132149	0.315174	10.385992	0.008744
CCNB3	si-NC	1.000142±0.011905				
	si-*CHAC1*	0.825892±0.019211	0.111499	0.237001	7.709780	0.001523
P21	si-NC	1.003773±0.060418				
	si-*CHAC1*	1.308000±0.088328	-0.601348	-0.007106	-2.842845	0.046733
CDKN1A	si-NC	1.006632±0.079749				
	si-*CHAC1*	1.307066±0.084705	-0.623444	0.022575	-2.582397	0.061172

Supplementary Table 32 The statistical results of CHAC1 and CDK1 protein expression levels were detected after overexpression and interference of *CHAC1*.

Index	Treatment	Mean±S.E.M	95% CI		*t*	*p*-value
			Down	Up		
CHAC1	pcDNA3.1-3xFlag	1.000000±0.357970				
	pcDNA3.1-*CHAC1*-3xFlag	2.467916±0.298863	-2.762650	-0.173181	-3.147816	0.034587
	si-NC	1.000000±0.202447				
	si-*CHAC1*	0.338458±0.120626	0.007247	1.315837	2.807198	0.048455
CDK1	pcDNA3.1-3xFlag	1.000000±0.098927				
	pcDNA3.1-*CHAC1*-3xFlag	1.463781±0.100278	-0.854878	-0.072683	-3.292431	0.030147
	si-NC	1.000000±0.142806				
	si-*CHAC1*	0.540649±0.066741	0.021696	0.897007	2.914079	0.043500

Supplementary Table 33 Statistical analysis of cell cycle after overexpression of *CHAC1* in myoblasts.

Index	Treatment	Mean±S.E.M	95% CI		*t*	*p*-value
			Down	Up		
G0/G1	si-NC	78.185000±0.675679				
	si-*CHAC1*	80.035000±0.257051	-3.888252	0.188252	-2.559058	0.065068
S	si-NC	13.957500±0.067623				
	si-*CHAC1*	15.107500±0.149687	-1.551913	-0.748087	-7.001379	0.000423
G2	si-NC	7.857500±0.730735				
	si-*CHAC1*	4.855000±0.287098	1.081405	4.923595	3.824304	0.008717

Supplementary Table 34 Statistical analysis of cell viability detected by CCK8 after overexpression of *CHAC1* in myoblasts.

Index	Treatment	Mean±S.E.M	95% CI		*t*	*p*-value
			Down	Up		
12 h	pcDNA3.1-3xFlag	0.510167±0.018721				
	pcDNA3.1-*CHAC1*-3xFlag	0.682286±0.030513	-0.254360	-0.089878	-4.606356	0.000757
24 h	pcDNA3.1-3xFlag	0.571833±0.013612				
	pcDNA3.1-*CHAC1*-3xFlag	0.845250±0.022930	-0.332169	-0.214664	-10.253413	6.1803E-7
36 h	pcDNA3.1-3xFlag	0.641500±0.012902				
	pcDNA3.1-*CHAC1*-3xFlag	0.741286±0.012118	-0.138791	-0.060781	-5.630766	0.000153
48 h	pcDNA3.1-3xFlag	0.484286±0.016202				
	pcDNA3.1-*CHAC1*-3xFlag	0.627875±0.010794	-0.184671	-0.102507	-7.550918	0.000004

Supplementary Table 35 Statistical analysis of cell viability detected by CCK8 after interfering with *CHAC1* in myoblasts.

Index	Treatment	Mean±S.E.M	95% CI		t	p-value
			Down	Up		
12 h	si-NC	0.508400±0.012532				
	si-*CHAC1*	0.485800±0.002518	-0.011871	0.057071	1.768001	0.146424
24 h	si-NC	0.563600± 0.008897				
	si-*CHAC1*	0.458400±0.013640	0.067645	0.142755	6.459704	0.000196
36 h	si-NC	0.750800±0.037598				
	si-*CHAC1*	0.638800±0.068932	-0.078734	0.302734	1.426401	0.202209
48 h	si-NC	0 0.914200±0.008429				
	si-*CHAC1*	0.797600± 0.024055	0.057822	0.175378	4.574484	0.001815

Supplementary Table 36 Statistical analysis of cell proliferation detected by EdU after overexpression and interference of *CHAC1* in myoblasts.

Index	Treatment	Mean±S.E.M	95% CI		*t*	*p*-value
			Down	Up		
EdU	pcDNA3.1-3xFlag	1.000000±0.034597				
	pcDNA3.1-*CHAC1*-3xFlag	1.452188±0.058392	-0.616541	-0.287834	-6.662361	0.000460
	si-NC	1.000000±0.016103				
	si-*CHAC1*	0.782511±0.004489	0.171076	0.263903	13.010179	0.000201

Supplementary Table 37 Statistical analysis of MyHC immunofluorescence after overexpression and interference of *CHAC1* in myoblasts.

Index	Treatment	Mean±S.E.M	95% CI		*t*	*p*-value
			Down	Up		
Immunofluorescence	pcDNA3.1-3xFlag	6.215515±0.378626				
	pcDNA3.1-*CHAC1*-3xFlag	8.080818±0.287505	-3.185259	-0.545346	-3.923547	0.017196
	si-NC	11.457343±0.131948				
	si-*CHAC1*	7.551422±0.091522	3.460076	4.351767	24.323617	0.000017

Supplementary Table 38 Statistical analysis of RNA expression levels of differentiation marker genes *MyoD*, *MyoG* and *MyHC* were detected after overexpression of *CHAC1* in myoblasts.

Index	Treatment	Mean±S.E.M	95% CI		*t*	*p*-value
			Down	Up		
MyoG	pcDNA3.1-3xFlag	1.001090±0.023463				
	pcDNA3.1-*CHAC1*-3xFlag	1.335116±0.009649	-0.398595	-0.269455	-13.166243	0.000036
MyoD	pcDNA3.1-3xFlag	1.000150±0.009967				
	pcDNA3.1-*CHAC1*-3xFlag	1.501753±0.043487	-0.599920	-0.403287	-13.114883	0.000046
MyHC	pcDNA3.1-3xFlag	1.003810±0.050959				
	pcDNA3.1-*CHAC1*-3xFlag	1.847724±0.062577	-1.041383	-0.646444	-10.457230	0.000045
Myomaker	pcDNA3.1-3xFlag	1.000203±0.011559				
	pcDNA3.1-*CHAC1*-3xFlag	1.366444±0.100220	-0.682644	-0.049838	-3.630316	0.034457

Supplementary Table 39 Statistical analysis of RNA expression levels of differentiation marker genes *MyoD, MyoG* and *MyHC* were detected after interfering with *CHAC1* in myoblasts.

Index	Treatment	Mean±S.E.M	95% CI		*t*	*p*-value
			Down	Up		
MyHC	si-NC	1.002857±0.053663				
	si-*CHAC1*	0.652382±0.072100	0.100933	0.600018	3.899440	0.017550
MyoG	si-NC	1.000178±0.013390				
	si-*CHAC1*	0.796148±0.003322	0.165728	0.242333	-13.114883	0.000122
MyoD	si-NC	1.000015±0.003871				
	si-*CHAC1*	0.768881±0.047492	0.028669	0.433599	4.850699	0.038934
Myomaker	si-NC	1.000301±0.017414				
	si-*CHAC1*	0.402463±0.016287	0.531637	0.664038	25.073332	0.000015

Supplementary Table 40 The statistical results of MyHC protein expression level after overexpression and interference of *CHAC1* in myoblasts.

Treatment	MyHC				
	Mean±S.E.M	95% CI		*t*	*p*-value
		Down	Up		
pcDNA3.1-3xFlag	1.000000±0.199195				
pcDNA3.1-*CHAC1*-3xFlag	3.342836±0.282298	-3.302099	-1.383572	-6.780987	0.002469
si-NC	1.000000±0.131869				
si-*CHAC1*	0.557421±0.062876	0.036964	0.848193	3.029467	0.038801

Supplementary Table 41 Statistical results of GAS fiber diameter.

Index	Treatment	Mean±S.E.M	95% CI		*t*	*p*-value
			Down	Up		
1d	Control	14.052933±0.096524				
	CHAC1	18.524573±0.107894	-4.873583	-4.069697	-30.888128	0.000007
3d	Control	20.728970±0.195283				
	CHAC1	25.196567±0.078711	-5.052174	-3.883019	-21.218818	0.000029
5d	Control	26.968860±0.364151				
	CHAC1	32.512960±0.275611	-6.812080	-4.276120	-12.139693	0.000264
7d	Control	33.255283±0.434962				
	CHAC1	37.337197±0.190807	-5.400648	-2.763179	-8.594002	0.001007

Supplementary Table 42 The statistical results of GAS muscle fiber CSA.

Index	Treatment	Mean ± S.E.M	95% CI		*t*	*p*-value
			Down	Up		
1d	Control	166.287033 ± 2.262520				
	CHAC1	279.833300 ± 2.852374	-123.654601	-103.437933	-31.187630	0.000006
3d	Control	351.342600 ± 5.873685				
	CHAC1	522.092600 ± 3.410616	-189.607862	-151.892138	-25.139541	0.000015
5d	Control	599.138867 ± 14.330294				
	CHAC1	876.611100 ± 20.523332	-346.970180	-207.974287	-11.085024	0.000377
7d	Control	912.287067 ± 25.990372				
	CHAC1	1179.537333 ± 6.464311	-341.609600	0.971758	-9.978649	0.000567

Supplementary Table 43 Statistical results of *CHAC1* mRNA expression levels.

Index	Treatment	Mean ± S.E.M	95% CI		*t*	*p*-value
			Down	Up		
1d	Control	1.004487 ± 0.068184				
	CHAC1	4.773897 ± 0.379710	-4.840516	-2.698304	-9.770801	0.000615
3d	Control	2.320190 ± 0.163627				
	CHAC1	18.567063 ± 1.175392	-19.541755	-12.951992	-13.690494	0.000165
5d	Control	2.265352 ± 0.356827				
	CHAC1	9.170909 ± 1.871700	-14.594446	0.783331	-3.624185	0.061552
7d	Control	0.984503 ± 0.096584				
	CHAC1	4.008078 ± 1.435765	-7.018907	0.971758	-2.101149	0.103519

Supplementary Table 44 Statistical results of mRNA expression level of muscle regeneration marker gene *adult MyHC*.

Index	Treatment	Mean±S.E.M	95% CI		*t*	*p*-value
			Down	Up		
1d	Control	1.032288±0.186904				
	CHAC1	3.347948±0.405762	-3.556007	-1.075314	-5.183475	0.006591
3d	Control	2.536293±0.420356				
	CHAC1	11.214431±2.951556	-16.955662	-0.400613	-2.910819	0.043642
5d	Control	2.991153±0.756796				
	CHAC1	9.480198±3.462267	-20.533820	7.555729	-1.830988	0.197449
7d	Control	1.669116±0.143899				
	CHAC1	4.663037±1.010370	-7.225082	1.237240	-2.933589	0.094673

Supplementary Table 45 Statistical results of mRNA expression level of muscle regeneration marker gene *eMyHC*.

Index	Treatment	Mean±S.E.M	95% CI		*t*	*p*-value
			Down	Up		
1d	Control	1.015170±0.128509				
	CHAC1	2.623569±0.793312	-4.906250	1.689453	-2.001359	0.176929
3d	Control	1.428196±0.065645				
	CHAC1	4.038873±0.117447	-2.984241	-2.237113	-19.403372	0.000042
5d	Control	2.170250±0.442591				
	CHAC1	7.255456±1.553187	-9.569210	-0.601203	-3.148703	0.034557
7d	Control	0.985700±0.085006				
	CHAC1	1.321130±0.081241	-0.661897	-0.008963	-2.852669	0.046271

Supplementary Table 46 Statistical results of mRNA expression level of muscle regeneration marker gene *Desmin*.

Index	Treatment	Mean±S.E.M	95% CI		*t*	*p*-value
			Down	Up		
1d	Control	1.022136±0.142413				
	CHAC1	3.271753±0.370356	-3.351293	-1.147942	-5.669493	0.004774
3d	Control	1.388628±0.204832				
	CHAC1	4.038873±0.117447	-13.844475	1.963472	-3.162084	0.084515
5d	Control	2.713278±0.512136				
	CHAC1	17.374030±2.629343	-22.098169	-7.223335	-5.472972	0.005424
7d	Control	1.045211±0.131187				
	CHAC1	3.754099±1.235725	-7.944300	2.526523	-2.179896	0.158271

Supplementary Table 47 Statistical results of CHAC1 protein expression levels.

Index	Treatment	Mean±S.E.M	95% CI		*t*	*p*-value
			Down	Up		
1d	Control	1.000000±0.117221				
	CHAC1	3.794247±0.959994	-5.479413	-0.109081	-2.889234	0.044597
3d	Control	0.895808±0.185533				
	CHAC1	13.614607±0.235162	-13.550452	-11.887145	-42.461239	0.000002
5d	Control	0.833233±0.101024				
	CHAC1	10.202378±0.663678	-11.233036	-7.505255	-13.956248	0.000153
7d	Control	1.045211±0.131187				
	CHAC1	3.754099±1.235725	-11.426140	-2.289924	-6.407507	0.023010

Supplementary Table 48 Statistical results of Desmin protein expression levels.

Index	Treatment	Mean±S.E.M	95% CI		*t*	*p*-value
			Down	Up		
1d	Control	1.000000±0.089454				
	CHAC1	8.410085±1.489342	-11.552612	-3.267558	-4.966460	0.007671
3d	Control	1.046700±0.044607				
	CHAC1	13.614607±0.235162	-16.157274	-5.516388	-5.655143	0.004818
5d	Control	5.944857±2.099266				
	CHAC1	21.424717±1.706115	-22.990514	-7.969206	-5.722402	0.004615
7d	Control	1.214242±0.134983				
	CHAC1	4.241297±1.558207	-7.369534	1.315424	-1.935404	0.125036

Supplementary Table 49 Statistical results of *CHAC1* mRNA expression levels after co-transfection of si-*lncMDP1*, si-NC, miR-301a-5p mimic, and mimic NC.

Treatment	*CHAC1*				
	Mean±S.E.M	95% CI Down	95% CI Up	*t*	*p*-value
si-NC + mimic NC	1.017863±0.029465				
si-*lncMDP1* + mimic NC	0.644985±0.027279	0.261392	0.484364	9.286128	0.000748
si-NC + miR-301a-5p mimic	0.807115±0.051709	0.045507	0.375988	3.541074	0.023990
si-*lncMDP1* + miR-301a-5p mimic	0.829186±0.053137	0.019980	0.357374	3.105273	0.036036
si-NC + miR-301a-5p mimic	0.807115±0.051709				
si-*lncMDP1* + miR-301a-5p mimic	0.829186±0.053137	-0.227929	0.183788	-0.297666	0.780778

Supplementary Table 50 Statistical results of *CDK1* mRNA expression levels after co-transfection of si-*lncMDP1*, si-NC, miR-301a-5p mimic, and mimic NC.

Treatment	*CDK1*				
	Mean±S.E.M	95% CI		*t*	*p*-value
		Down	Up		
si-NC + mimic NC	1.001025±0.032322				
si-*lncMDP1* + mimic NC	0.690327±0.055697	0.131906	0.489489	4.824813	0.008493
si-NC + miR-301a-5p mimic	0.753091±0.026967	0.131061	0.364807	5.889955	0.004155
si-*lncMDP1* + miR-301a-5p mimic	0.736449±0.044505	0.111863	0.417289	4.810190	0.008584
si-NC + miR-301a-5p mimic	0.753091±0.026967				
si-*lncMDP1* + miR-301a-5p mimic	0.736449±0.044505	-0.127837	0.161121	0.319808	0.765121

Supplementary Table 51 Statistical results of *PCNA* mRNA expression levels after co-transfection of si-*lncMDP1*, si-NC, miR-301a-5p mimic, and mimic NC.

Treatment	*PCNA*				
	Mean±S.E.M	95% CI		*t*	*p*-value
		Down	Up		
si-NC + mimic NC	1.000281±0.016688				
si-*lncMDP1* + mimic NC	0.761997±0.042998	0.110226	0.366343	5.166262	0.006669
si-NC + miR-301a-5p mimic	0.829884±0.056768	0.006116	0.334678	2.879808	0.045022
si-*lncMDP1* + miR-301a-5p mimic	0.830744±0.031361	0.070905	0.268169	4.772383	0.008825
si-NC + miR-301a-5p mimic	0.829884±0.056768				
si-*lncMDP1* + miR-301a-5p mimic	0.830744±0.031361	-0.180924	0.179204	-0.013260	0.990055

Supplementary Table 52 Statistical results of *CCND1* mRNA expression levels after co-transfection of si-*lncMDP1*, si-NC, miR-301a-5p mimic, and mimic NC.

Treatment	*CCND1*				
	Mean±S.E.M	95% CI		*t*	*p*-value
		Down	Up		
si-NC + mimic NC	1.000642±0.025433				
si-*lncMDP1* + mimic NC	0.804934±0.065133	0.001573	0.389844	2.798937	0.048864
si-NC + miR-301a-5p mimic	0.850962±0.030907	0.009347	0.260810	3.739594	0.020128
si-*lncMDP1* + miR-301a-5p mimic	0.873664±0.027173	0.023644	0.230312	3.411728	0.026984
si-NC + miR-301a-5p mimic	0.829884±0.056768				
si-*lncMDP1* + miR-301a-5p mimic	0.830744±0.031361	-0.136962	0.091557	-0.551661	0.610549

Supplementary Table 53 Statistical results of *CCNB1* mRNA expression levels after co-transfection of si-*lncMDP1*, si-NC, miR-301a-5p mimic, and mimic NC.

Treatment	*CCNB1*				
	Mean±S.E.M	95% CI		*t*	*p*-value
		Down	Up		
si-NC + mimic NC	1.001749±0.042366				
si-*lncMDP1* + mimic NC	0.589592±0.021329	0.280465	0.543850	8.689436	0.000966
si-NC + miR-301a-5p mimic	0.693456±0.038246	0.149826	0.466760	5.401493	0.005687
si-*lncMDP1* + miR-301a-5p mimic	0.806358±0.033343	0.045704	0.345078	3.624183	0.022274
si-NC + miR-301a-5p mimic	0.693456±0.038246				
si-*lncMDP1* + miR-301a-5p mimic	0.806358±0.033343	-0.253777	0.027973	-2.225138	0.090096

Supplementary Table 54 Statistical results of *P21* mRNA expression levels after co-transfection of si-*lncMDP1*, si-NC, miR-301a-5p mimic, and mimic NC.

Treatment	*P21*				
	Mean±S.E.M	95% CI Down	95% CI Up	*t*	*p*-value
si-NC + mimic NC	1.002254±0.047679				
si-*lncMDP1* + mimic NC	1.317394±0.016934	-0.455621	-0.174659	-6.228378	0.003384
si-NC + miR-301a-5p mimic	1.319840±0.026152	-0.468570	-0.166601	-5.840063	0.004286
si-*lncMDP1* + miR-301a-5p mimic	1.222144±0.060800	-0.434413	-0.005366	-2.845890	0.046589
si-NC + miR-301a-5p mimic	0.693456±0.038246				
si-*lncMDP1* + miR-301a-5p mimic	0.806358±0.033343	-0.086065	0.281457	1.476095	0.213960

Supplementary Table 55 Statistical results of *MyHC* mRNA expression levels after co-transfection of si-*lncMDP1*, si-NC, miR-301a-5p mimic, and mimic NC.

Treatment	*MyHC*				
	Mean±S.E.M	95% CI		*t*	*p*-value
		Down	Up		
si-NC + mimic NC	1.000063±0.007938				
si-*lncMDP1* + mimic NC	0.711093±0.010721	0.251931	0.326007	21.661742	0.000027
si-NC + miR-301a-5p mimic	0.746592±0.070256	0.057168	0.449774	3.585019	0.023064
si-*lncMDP1* + miR-301a-5p mimic	0.775387±0.020006	0.164918	0.284434	10.438727	0.000476
si-NC + miR-301a-5p mimic	0.746592±0.070256				
si-*lncMDP1* + miR-301a-5p mimic	0.775387±0.020006	-0.231611	0.174021	-0.394189	0.713554

Supplementary Table 56 Statistical results of *MyoG* mRNA expression levels after co-transfection of si-*lncMDP1*, si-NC, miR-301a-5p mimic, and mimic NC.

Treatment	*MyoG*				
	Mean±S.E.M	95% CI		*t*	*p*-value
		Down	Up		
si-NC + mimic NC	1.000543±0.023133				
si-*lncMDP1* + mimic NC	0.718349±0.089646	0.025143	0.539246	3.048022	0.038102
si-NC + miR-301a-5p mimic	0.827736±0.022672	0.082876	0.262739	5.335076	0.005945
si-*lncMDP1* + miR-301a-5p mimic	0.878833±0.014433	0.046006	0.197415	4.463711	0.011128
si-NC + miR-301a-5p mimic	0.827736±0.022672				
si-*lncMDP1* + miR-301a-5p mimic	0.878833±0.014433	-0.125717	0.023523	-1.901198	0.130059

Supplementary Table 57 Statistical results of *Myomaker* mRNA expression levels after co-transfection of si-*lncMDP1*, si-NC, miR-301a-5p mimic, and mimic NC.

Treatment	*Myomaker*				
	Mean±S.E.M	95% CI		*t*	*p*-value
		Down	Up		
si-NC + mimic NC	1.001624±0.039983				
si-*lncMDP1* + mimic NC	0.506291±0.005774	0.383170	0.607497	12.261289	0.000254
si-NC + miR-301a-5p mimic	0.545216±0.060880	0.254183	0.658633	6.266253	0.003309
si-*lncMDP1* + miR-301a-5p mimic	0.666665±0.105494	0.021730	0.648189	2.969058	0.041183
si-NC + miR-301a-5p mimic	0.545216±0.060880				
si-*lncMDP1* + miR-301a-5p mimic	0.666665±0.105494	-0.459621	0.216724	-0.997112	0.375143

Supplementary Table 58 Statistical results of CHAC1 protein expression levels after co-transfection of si-*lncMDP1*, si-NC, miR-301a-5p mimic, and mimic NC.

Treatment	CHAC1				
	Mean±S.E.M	95% CI		*t*	*p*-value
		Down	Up		
si-NC + mimic NC	1.000000±0.070665				
si-*lncMDP1* + mimic NC	0.379125±0.099047	0.283064	0.958686	5.102922	0.006968
si-NC + miR-301a-5p mimic	0.554756±0.038093	0.222356	0.668131	5.546268	0.005169
si-*lncMDP1* + miR-301a-5p mimic	0.675187±0.038659	0.101176	0.548450	4.032537	0.015701
si-NC + miR-301a-5p mimic	0.554756±0.038093				
si-*lncMDP1* + miR-301a-5p mimic	0.675187±0.038659	-0.271117	0.030255	-2.218983	0.090715

Supplementary Table 59 Statistical results of CDK1 protein expression levels after co-transfection of si-*lncMDP1*, si-NC, miR-301a-5p mimic, and mimic NC.

Treatment	CDK1				
	Mean ± S.E.M	95% CI		*t*	*p*-value
		Down	Up		
si-NC + mimic NC	1.000000 ± 0.073657				
si-*lncMDP1* + mimic NC	0.344970 ± 0.063539	0.384949	0.925113	6.733717	0.002534
si-NC + miR-301a-5p mimic	0.370893 ± 0.065007	0.356346	0.901869	6.403697	0.003054
si-*lncMDP1* + miR-301a-5p mimic	0.625729 ± 0.107859	0.011640	0.736902	2.865567	0.045672
si-NC + miR-301a-5p mimic	0.370893 ± 0.065007				
si-*lncMDP1* + miR-301a-5p mimic	0.625729 ± 0.107859	-0.604486	0.094813	-2.023566	0.113038

Supplementary Table 60 Statistical results of MyHC protein expression levels after co-transfection of si-*lncMDP1*, si-NC, miR-301a-5p mimic, and mimic NC.

Treatment	MyHC				
	Mean±S.E.M	95% CI		*t*	*p*-value
		Down	Up		
si-NC + mimic NC	1.000000±0.115609				
si-*lncMDP1* + mimic NC	0.265091±0.082596	0.340424	1.129395	5.172394	0.006641
si-NC + miR-301a-5p mimic	0.348944±0.071387	0.273811	1.028301	4.791633	0.008701
si-*lncMDP1* + miR-301a-5p mimic	0.552048±0.108654	0.007457	0.888447	2.823447	0.047661
si-NC + miR-301a-5p mimic	0.348944±0.071387				
si-*lncMDP1* + miR-301a-5p mimic	0.552048±0.108654	-0.564059	0.157852	-1.562258	0.193259

Supplementary Table 61 Statistical results of *CHAC1* mRNA expression levels after co-transfection of si-*lncMDP1*, si-NC, miR-301a-5p inhibitor, and inhibitor NC.

Treatment	*CHAC1*				
	Mean±S.E.M	95% CI		*t*	*p*-value
		Down	Up		
si-NC + inhibitor NC	1.001132±0.034027	0.023057	0.385793	3.129408	0.035205
si-*lncMDP1* + inhibitor NC	0.796707±0.055762				
si-*lncMDP1* + inhibitor NC	0.796707±0.055762	-1.209138	-0.528244	-7.084433	0.002096
si-*lncMDP1* + miR-301a-5p inhibitor	1.665398±0.109207				

Supplementary Table 62 Statistical results of *CDK1* mRNA expression levels after co-transfection of si-*lncMDP1*, si-NC, miR-301a-5p inhibitor, and inhibitor NC.

Treatment	*CHAC1*				
	Mean±S.E.M	95% CI		*t*	*p*-value
		Down	Up		
si-NC + inhibitor NC	1.000460±0.021401				
si-*lncMDP1* + inhibitor NC	0.557913±0.076198	0.222801	0.662293	5.591484	0.005020
si-*lncMDP1* + inhibitor NC	0.557913±0.076198				
si-*lncMDP1* + miR-301a-5p inhibitor	2.238742±0.018312	-1.898412	-1.463246	-21.447998	0.000028

Supplementary Table 63 Statistical results of *PCNA* mRNA expression levels after co-transfection of si-*lncMDP1*, si-NC, miR-301a-5p inhibitor, and inhibitor NC.

Treatment	*PCNA*				
	Mean ± S.E.M	95% CI		*t*	*p*-value
		Down	Up		
si-NC + inhibitor NC	1.001632 ± 0.040278	0.047236	0.422348	3.475699	0.025451
si-*lncMDP1* + inhibitor NC	0.766840 ± 0.054231				
si-*lncMDP1* + inhibitor NC	0.766840 ± 0.054231	-0.807742	-0.292260	-5.924729	0.004066
si-*lncMDP1* + miR-301a-5p inhibitor	1.316841 ± 0.075344				

Supplementary Table 64 Statistical results of *CCNB2* mRNA expression levels after co-transfection of si-*lncMDP1*, si-NC, miR-301a-5p inhibitor, and inhibitor NC.

Treatment	*CCNB2*				
	Mean±S.E.M	95% CI		*t*	*p*-value
		Down	Up		
si-NC + inhibitor NC	1.000525±0.022902	0.194319	0.397354	8.090942	0.001268
si-*lncMDP1* + inhibitor NC	0.704688±0.028503				
si-*lncMDP1* + inhibitor NC	0.704688±0.028503	-1.026050	-0.343412	-5.569920	0.005090
si-*lncMDP1* + miR-301a-5p inhibitor	1.389419±0.119584				

Supplementary Table 65 Statistical results of *CCNB3* mRNA expression levels after co-transfection of si-*lncMDP1*, si-NC, miR-301a-5p inhibitor, and inhibitor NC.

Treatment	*CCNB3*				
	Mean±S.E.M	95% CI		*t*	*p*-value
		Down	Up		
si-NC + inhibitor NC	1.000547±0.023209				
si-*lncMDP1* + inhibitor NC	0.483369±0.043822	0.379498	0.654857	10.429383	0.000477
si-*lncMDP1* + inhibitor NC	0.704688±0.028503				
si-*lncMDP1* + miR-301a-5p inhibitor	1.591889±0.208601	-1.700331	-0.516709	-5.200551	0.006514

Supplementary Table 66 Statistical results of *P21* mRNA expression levels after co-transfection of si-*lncMDP1*, si-NC, miR-301a-5p inhibitor, and inhibitor NC.

Treatment	*P21*				
	Mean±S.E.M	95% CI		*t*	*p*-value
		Down	Up		
si-NC + inhibitor NC	1.000178±0.013385	-1.573646	0.142692	-3.557090	0.069837
si-*lncMDP1* + inhibitor NC	1.715655±0.200695				
si-*lncMDP1* + inhibitor NC	1.715655±0.200695	0.339546	1.538152	4.349492	0.012162
si-*lncMDP1* + miR-301a-5p inhibitor	0.776806±0.079459				

Supplementary Table 67 Statistical results of *MyHC* mRNA expression levels after co-transfection of si-*lncMDP1*, si-NC, miR-301a-5p inhibitor, and inhibitor NC.

Treatment	*MyHC*				
	Mean±S.E.M	95% CI		*t*	*p*-value
		Down	Up		
si-NC + inhibitor NC	1.000189±0.013815				
si-*lncMDP1* + inhibitor NC	0.874753±0.038361	0.012234	0.238639	3.076496	0.037058
si-*lncMDP1* + inhibitor NC	0.874753±0.038361				
si-*lncMDP1* + miR-301a-5p inhibitor	2.044926±0.072589	-1.398125	-0.942223	-14.252724	0.000141

Supplementary Table 68 Statistical results of *MyoD* mRNA expression levels after co-transfection of si-*lncMDP1*, si-NC, miR-301a-5p inhibitor, and inhibitor NC.

Treatment	*MyoD*				
	Mean±S.E.M	95% CI		*t*	*p*-value
		Down	Up		
si-NC + inhibitor NC	1.001189±0.034750				
si-*lncMDP1* + inhibitor NC	0.701350±0.032846	0.167080	0.432597	6.270674	0.003301
si-*lncMDP1* + inhibitor NC	0.874753±0.038361				
si-*lncMDP1* + miR-301a-5p inhibitor	1.625724±0.088814	-1.187284	-0.661463	-9.761762	0.000617

Supplementary Table 69 Statistical results of *MyoG* mRNA expression levels after co-transfection of si-*lncMDP1*, si-NC, miR-301a-5p inhibitor, and inhibitor NC.

Treatment	*MyoG*				
	Mean±S.E.M	95% CI		*t*	*p*-value
		Down	Up		
si-NC + inhibitor NC	1.000295±0.017262	0.035029	0.270505	3.602494	0.022708
si-*lncMDP1* + inhibitor NC	0.847527±0.038734				
si-*lncMDP1* + inhibitor NC	0.874753±0.038361	-0.744490	-0.477784	-12.724039	0.000220
si-*lncMDP1* + miR-301a-5p inhibitor	1.458664±0.028401				

Supplementary Table 70 Statistical results of *Myomaker* mRNA expression levels after co-transfection of si-*lncMDP1*, si-NC, miR-301a-5p inhibitor, and inhibitor NC.

Treatment	*Myomaker*				
	Mean±S.E.M	95% CI		*t*	*p*-value
		Down	Up		
si-NC + inhibitor NC	1.000276±0.016623	0.043463	0.377040	3.499951	0.024897
si-*lncMDP1* + inhibitor NC	0.790024±0.057727				
si-*lncMDP1* + inhibitor NC	0.790024±0.057727	-0.653706	-0.317788	-8.029649	0.001305
si-*lncMDP1* + miR-301a-5p inhibitor	1.275772±0.018088				

Supplementary Table 71 Statistical results of CHAC1 protein expression levels after co-transfection of si-*lncMDP1*, si-NC, miR-301a-5p inhibitor, and inhibitor NC.

Treatment	CHAC1				
	Mean±S.E.M	95% CI		*t*	*p*-value
		Down	Up		
si-NC + inhibitor NC	1.000000±0.078534				
si-*lncMDP1* + inhibitor NC	0.484249±0.146198	0.054983	0.976519	3.107753	0.035950
si-*lncMDP1* + inhibitor NC	0.484249±0.146198				
si-*lncMDP1* + miR-301a-5p inhibitor	1.447517±0.066498	-1.409195	-0.517341	-5.997530	0.003888

Supplementary Table 72 Statistical results of CDK1 protein expression levels after co-transfection of si-*lncMDP1*, si-NC, miR-301a-5p inhibitor, and inhibitor NC.

Treatment	CDK1				
	Mean±S.E.M	95% CI		*t*	*p*-value
		Down	Up		
si-NC + inhibitor NC	1.000000±0.073788				
si-*lncMDP1* + inhibitor NC	0.381704±0.020546	0.405634	0.830959	8.072265	0.001279
si-*lncMDP1* + inhibitor NC	0.484249±0.146198				
si-*lncMDP1* + miR-301a-5p inhibitor	1.322154±0.127490	-1.298986	-0.581914	-7.282696	0.001889

Supplementary Table 73 Statistical results of MyHC protein expression levels after co-transfection of si-*lncMDP1*, si-NC, miR-301a-5p inhibitor, and inhibitor NC.

Treatment	MyHC				
	Mean±S.E.M	95% CI Down	95% CI Up	*t*	*p*-value
si-NC + inhibitor NC	1.000000±0.067038	0.079907	0.480720	3.883483	0.017789
si-*lncMDP1* + inhibitor NC	0.719687±0.026757				
si-*lncMDP1* + inhibitor NC	0.484249±0.146198	-1.078543	-0.600990	-9.764628	0.000616
si-*lncMDP1* + miR-301a-5p inhibitor	1.559453±0.081733				
