# Supplementary material for: Whole transcriptome profiling reveals a lncMDP1 that regulates myogenesis by adsorbing miR-301a-5p targeting CHAC1
Source: Commun Biol. 2024 May 2;7:518. doi: 10.1038/s42003-024-06226-1 (PMC11066001; doi:10.1038/s42003-024-06226-1)
Supplement: Supplementary file 5 — Reporting Summary [file 42003_2024_6226_MOESM5_ESM.pdf]

nature portfolio

Corresponding author(s): DAPR and COMMSBIO-23-3400B Zhuanjian Li

Last updated by author(s): Jan 24, 2024

Reporting Summary

Nature Portfolio wishes to improve the reproducibility of the work that we publish. This form provides structure for consistency and transparency in reporting. For further information on Nature Portfolio policies, see our Editorial Policies and the Editorial Policy Checklist.

Statistics

For all statistical analyses, confirm that the following items are present in the figure legend, table legend, main text, or Methods section.

n/a	Confirmed	
☐	☒	The exact sample size (*n*) for each experimental group/condition, given as a discrete number and unit of measurement
☐	☒	A statement on whether measurements were taken from distinct samples or whether the same sample was measured repeatedly
☐	☒	The statistical test(s) used AND whether they are one- or two-sided<br>*Only common tests should be described solely by name; describe more complex techniques in the Methods section.*
☒	☐	A description of all covariates tested
☒	☐	A description of any assumptions or corrections, such as tests of normality and adjustment for multiple comparisons
☐	☒	A full description of the statistical parameters including central tendency (e.g. means) or other basic estimates (e.g. regression coefficient) AND variation (e.g. standard deviation) or associated estimates of uncertainty (e.g. confidence intervals)
☐	☒	For null hypothesis testing, the test statistic (e.g. *F*, *t*, *r*) with confidence intervals, effect sizes, degrees of freedom and *P* value noted<br>*Give P values as exact values whenever suitable.*
☒	☐	For Bayesian analysis, information on the choice of priors and Markov chain Monte Carlo settings
☒	☐	For hierarchical and complex designs, identification of the appropriate level for tests and full reporting of outcomes
☐	☒	Estimates of effect sizes (e.g. Cohen's *d*, Pearson's *r*), indicating how they were calculated

Our web collection on statistics for biologists contains articles on many of the points above.

Software and code

Policy information about availability of computer code

Data collection	Illumina HiSeqTM 4000
Data analysis	DESeq2; DESeq2; Cytoscape; SPSS

For manuscripts utilizing custom algorithms or software that are central to the research but not yet described in published literature, software must be made available to editors and reviewers. We strongly encourage code deposition in a community repository (e.g. GitHub). See the Nature Portfolio guidelines for submitting code & software for further information.

Data

Policy information about availability of data

All manuscripts must include a data availability statement. This statement should provide the following information, where applicable:

- Accession codes, unique identifiers, or web links for publicly available datasets
- A description of any restrictions on data availability
- For clinical datasets or third party data, please ensure that the statement adheres to our policy

All study data results are available from the text. Whole transcriptome sequencing results have been uploaded to the NCBI Database Sequence Read Archive under the login numbers PRJNA909444 and PRJNA908949.

Research involving human participants, their data, or biological material

Policy information about studies with human participants or human data. See also policy information about sex, gender (identity/presentation), and sexual orientation and race, ethnicity and racism.

Reporting on sex and gender	not applicable
Reporting on race, ethnicity, or other socially relevant groupings	not applicable
Population characteristics	not applicable
Recruitment	not applicable
Ethics oversight	not applicable

Note that full information on the approval of the study protocol must also be provided in the manuscript.

Field-specific reporting

Please select the one below that is the best fit for your research. If you are not sure, read the appropriate sections before making your selection.

☒ Life sciences ☐ Behavioural & social sciences ☐ Ecological, evolutionary & environmental sciences

For a reference copy of the document with all sections, see nature.com/documents/nr-reporting-summary-flat.pdf

Life sciences study design

All studies must disclose on these points even when the disclosure is negative.

Sample size	This study provides a sample size of three to increase the confidence of the experimental results. This is because three and more biological replicates above are biologically significant.
Data exclusions	This study has excluded data in CCK-8 as well as qPCR because of outliers in individual data
Replication	In this study, in cellular assays, firstly, we tested the overexpression and interference efficiency of genes as well as non-coding RNAs several times and the opposite series of results after overexpression and interference also confirmed the reproducibility of the assays.
Randomization	The cell experiments in this study used the same equipment specifications, the same cell growth, the same reagents, and the same number of groups. For the in vivo experiments, 21-week-old roosters with similar body weight and the same health condition were selected and randomly grouped in equal numbers.
Blinding	All participants in this study were informed during data collection and analysis.

Reporting for specific materials, systems and methods

We require information from authors about some types of materials, experimental systems and methods used in many studies. Here, indicate whether each material, system or method listed is relevant to your study. If you are not sure if a list item applies to your research, read the appropriate section before selecting a response.

Materials & experimental systems

n/a	Involved in the study	
☐	☒	Antibodies
☐	☒	Eukaryotic cell lines
☒	☐	Palaeontology and archaeology
☐	☒	Animals and other organisms
☒	☐	Clinical data
☒	☐	Dual use research of concern
☒	☐	Plants

Methods

n/a	Involved in the study	
☒	☐	ChIP-seq
☐	☒	Flow cytometry
☒	☐	MRI-based neuroimaging

Antibodies

Antibodies used	MYHC (1:100, B103, DSHB, Iowa City, USA); CDK1 (19532-1-AP, 1:1000; Proteintech); GAPDH (60004-1-Ig, 1:50000; Proteintech); Desmin (16520-1-AP; 1:5000; Proteintech); CHAC1 (15207-1-AP; 1:2000; Proteintech)
Validation	MYHC comes from the article "MiR-29b-1-5p regulates the proliferation and differentiation of chicken primary myoblasts and analysis of its effective targets". CDK1, GAPDH and CHAC1 data were obtained from the official website of Wuhan Sanying Biotechnology Co., Ltd "https://ptgcn.com/products/".

Eukaryotic cell lines

Policy information about cell lines and Sex and Gender in Research

Cell line source(s)	Chicken embryo fibroblasts (UMNSAH / DF-1), chicken primary myoblasts derived from Arbor Acres broiler embryo eggs.
Authentication	The cells used are authenticated within the subject group.
Mycoplasma contamination	The cells were not tested for mycoplasma contamination, and all cells in the study were observed under the microscope to be free of contamination and to have normal cell morphology without impurities.
Commonly misidentified lines (See ICLAC register)	not applicable

Animals and other research organisms

Policy information about studies involving animals; ARRIVE guidelines recommended for reporting animal research, and Sex and Gender in Research

Laboratory animals	11-embryo-old breeding eggs and 3-week-old roosters were AA broilers.
Wild animals	This study does not involve wild animals.
Reporting on sex	This study was not analysed for gender, except that the experiment used the same gender to ensure a consistent sample.
Field-collected samples	This study does not involve samples taken from the field.
Ethics oversight	The Animal Care and Use Committee (IACUC) of Henan Agricultural University

Note that full information on the approval of the study protocol must also be provided in the manuscript.

Flow Cytometry

Plots

Confirm that:

☐ The axis labels state the marker and fluorochrome used (e.g. CD4-FITC).

☐ The axis scales are clearly visible. Include numbers along axes only for bottom left plot of group (a 'group' is an analysis of identical markers).

☐ All plots are contour plots with outliers or pseudocolor plots.

☒ A numerical value for number of cells or percentage (with statistics) is provided.

Methodology

Sample preparation	After trypsin digestion, the cells were first added with 70 % ethanol and placed at – 20 ° C overnight. After centrifugation, PBS was hydrated, and then the cells were incubated with propidium iodide staining solution. Finally, the cell cycle was detected by flow cytometry.
Instrument	Beckman (CytoFLEX)
Software	Modfit
Cell population abundance	We used Modfit software analysis, the above does not show cell purity, CV value is about 4%.
Gating strategy	Modfit software analysis method, FSC-A/SSC-A gate to delineate the target cell group, then FSC-A/FSC-H to remove adhesion, PE-A/PE-H to delineate the target cell group, and PE-A/Count to display the peak map.

☒ Tick this box to confirm that a figure exemplifying the gating strategy is provided in the Supplementary Information.
